# Mechanoadaptation via Myosin Cytoplasmic Redistribution Protects Circulating Tumor Cells From Shear‐induced Death During Hematogenous Dissemination

**DOI:** 10.1002/advs.202523112

**Published:** 2026-03-30

**Authors:** Cunyu Zhang, Qianchun Wang, Keming Li, Guanshuo Hu, Ying Xin, Kai Tang, Bing Hu, Pengyu Du, Renwei Mao, Baohua Ji, Youhua Tan

**Affiliations:** ^1^ The Hong Kong Polytechnic University Shenzhen Research Institute Shenzhen China; ^2^ Research Institute of Smart Ageing The Hong Kong Polytechnic University Hong Kong China; ^3^ Department of Biomedical Engineering The Hong Kong Polytechnic University Hong Kong China; ^4^ Wenzhou Institute University of Chinese Academy of Sciences Wenzhou China; ^5^ Medical Engineering & Engineering Medicine Innovation Center Hangzhou International Innovation Institute Beihang University Hangzhou China; ^6^ Institute of Biomechanics and Applications Department of Engineering Mechanics Zhejiang University Hangzhou China; ^7^ Eye Center School of Medicine The Second Affiliated Hospital Zhejiang University Hangzhou China

**Keywords:** circulating tumor cell, fluid shear stress, force transmission, mechanoadaptation, mechanobiology, mechanotransduction

## Abstract

To initiate distant metastasis via hematogenous dissemination, circulating tumor cells (CTCs) must survive shear‐induced destruction in vasculature. However, how CTCs withstand such mechanical interrogation remains poorly understood. Using both patient‐derived primary cells and cancer cell lines, this study reports that non‐adherent tumor cells mechanically adapt to increasing fluid shear stress (FSS) through re‐distribution of activated myosin into cytoplasm. Cytoplasmic but not cortical myosin attenuates force transmission from cell surface into chromatin by disrupting the binding of myosin with actin, which is recapitulated by a cytoskeletal fluidization‐based model. Under high FSS, Lamin A/C‐mediated nuclear mechanosensing elevates nuclear envelop tension and triggers calcium release from endoplasmic reticulum, which redistributes myosin into cytoplasm through Rho‐associated protein kinase. Targeting cytoplasmic myosin‐mediated mechanoadaptation restores mechanoresponses and re‐sensitizes CTCs to shear‐induced death, which eventually reduces tumor metastasis. In summary, these results unveil the reduction of force transmission of CTCs in response to harsh shearing via cytoplasmic myosin accumulation, which potentiates mechanoadaptation and protects them from shear‐induced apoptosis during hematogenous metastasis.

## Graphical abstract

This study investigates how CTCs survive varying shear stress during hematogenous metastasis. We uncover a self‐protection mechanism, by which non‐adherent CTCs adapt to high shearing milieu through accumulated cytoplasmic myosin‐mediated disruption of myosin‐actin binding, attenuating force transmission into chromatin to protect CTCs from shear‐induced death. This study unveils mechanoadaptation as the target for development of mechanotherapeutic strategies against metastasis.

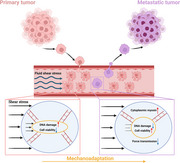

## 1 Introduction

Tumor cells metastasize to distant organs mainly through hematogenous dissemination and the number of circulating tumor cells (CTCs) is correlated with patient survival [1]. The majority of CTCs become apoptotic within vasculature, while a tiny subpopulation can persist and eventually grow into metastatic lesions [1, 2], which account for over 90% of cancer deaths. Therefore, unraveling the critical mechanisms underlying the survival of CTCs during hematogenous dissemination is essential for targeting their vulnerability in order to prevent metastasis.

After the escape from the primary lesion and entry into vasculature, CTCs become vulnerable to a variety of deteriorated microenvironmental cues, such as anoikis, immune surveillance, and cytokines [3]. Except these biochemical challenges, CTCs experience varying levels of fluid shear stress (FSS) in vasculature, which is a key limiting factor of their survival [4]. For example, the viability of non‐adherent colon tumor cells depends on the magnitudes of FSS and circulating time [5]. Shear stress decreases the viability of non‐adherent prostate cancer cells possibly via cell membrane damage and reduced membrane repair [6]. High FSS induces significant apoptosis but facilitates the migration and extravasation of CTCs [7, 8]. Increasing FSS disaggregates CTC clusters and reduces their viability [9]. Nevertheless, a subpopulation of tumor cells exhibit resistance to shear‐induced destruction. For example, non‐adherent breast cancer cells resist shear‐induced death in a Lamin A/C‐dependent manner [10]. RhoA activity enables prostate tumor cells to evade shear‐induced plasma membrane damage [11]. FSS upregulates desmocollin‐2 and plakophilin‐1 in breast and lung cancer cells, which facilitate CTC cluster formation and tumor metastasis [12]. Laminar FSS upregulates atonal bHLH transcription factor 8 in colorectal cancer cells, which promotes the survival and metastasis of CTCs via HK2‐mediated glycolysis [13]. These findings suggest a possibility that some CTCs may hold the ability to adapt themselves to varying levels of FSS and eventually survive this harsh mechanical interrogation, which is yet to be validated. Even if it is presumably possible, how this mechanoadaptation mechanism protects CTCs from shear‐induced apoptosis remains unclear.

Exogenous mechanical forces can be perceived and propagated into cell interior through cytoskeleton‐associated machinery, which modulates the interaction of actin filaments with non‐muscle myosin II to generate contractile forces [14]. Cytoskeletal tension stabilizes F‐actin network and facilitates focal adhesion maturation, and thus crucially orchestrates mechanotransduction [15]. For example, substrate rigidity promotes YAP activation and stem cell differentiation, which can be abolished by the inhibition of actomyosin tension [16]. Low cytoskeletal contractility mediates local niche softness‐induced brain metastasis via the activation of histone deacetylase 3 due to the repressed mechanotransduction [17]. α‐actinin crosslinks F‐actin into solid‐like stress fibers to generate contractile forces, which enable efficient force transmission and matrix rigidity sensing [18]. Interestingly, the subcellular distribution of myosin II is altered in certain cellular processes, which affects force generation and cellular functions. For example, during mitosis, myosin is redistributed into the cleavage furrow, leading to furrow stiffening, which is critical in cell division [19]. Spatial confinements deform the nucleus and recruit myosin II from cytoplasm to cortex, which increases cellular contractile force that enables cells to migrate through restrictive 3D microenvironments [20, 21]. Myosin II and Talin A are mainly distributed in the rear of migrating cells and colocalized, which favors force transmission to retract the rear and direct cell migration [22]. These results implicate the important but possibly distinct roles of subcellular myosin in mechano‐perception and force propagation. However, whether non‐adherent CTCs respond to varying levels of FSS via the redistribution of subcellular myosin remains unexplored.

In this study, we systematically investigated the survival and mechanoresponses of non‐adherent tumor cells to different levels of FSS. To dissect the underlying mechanism, the effect of varying FSS on subcellular distribution of activated myosin was examined. The force transmission from cell membrane to chromatin was evaluated by histone 2B or H2B displacement and force transmission efficiency (FTE). The influences of cytoplasmic and cortical myosin on FTE and the interaction of actin with myosin were elucidated, which was further explained by theoretical modeling of actin‐myosin interaction. Further, nuclear mechanosensing was demonstrated to mediate calcium response from endoplasmic reticulum and shear‐induced myosin redistribution. These mechanoadaptive responses were also observed in patient‐derived primary cells. Finally, targeting cytoplasmic myosin‐mediated mechanoadaptation re‐sensitized CTCs to shear‐induced apoptosis and suppressed tumor metastasis in vivo, suggesting the potential therapeutic roles of this unappreciated survival mechanism.

## 2 Results

### 2.1 Non‐Adherent Tumor Cells Adapt to High FSS by Reducing Mechanoresponses and Force Transmission

**FIGURE 1:**
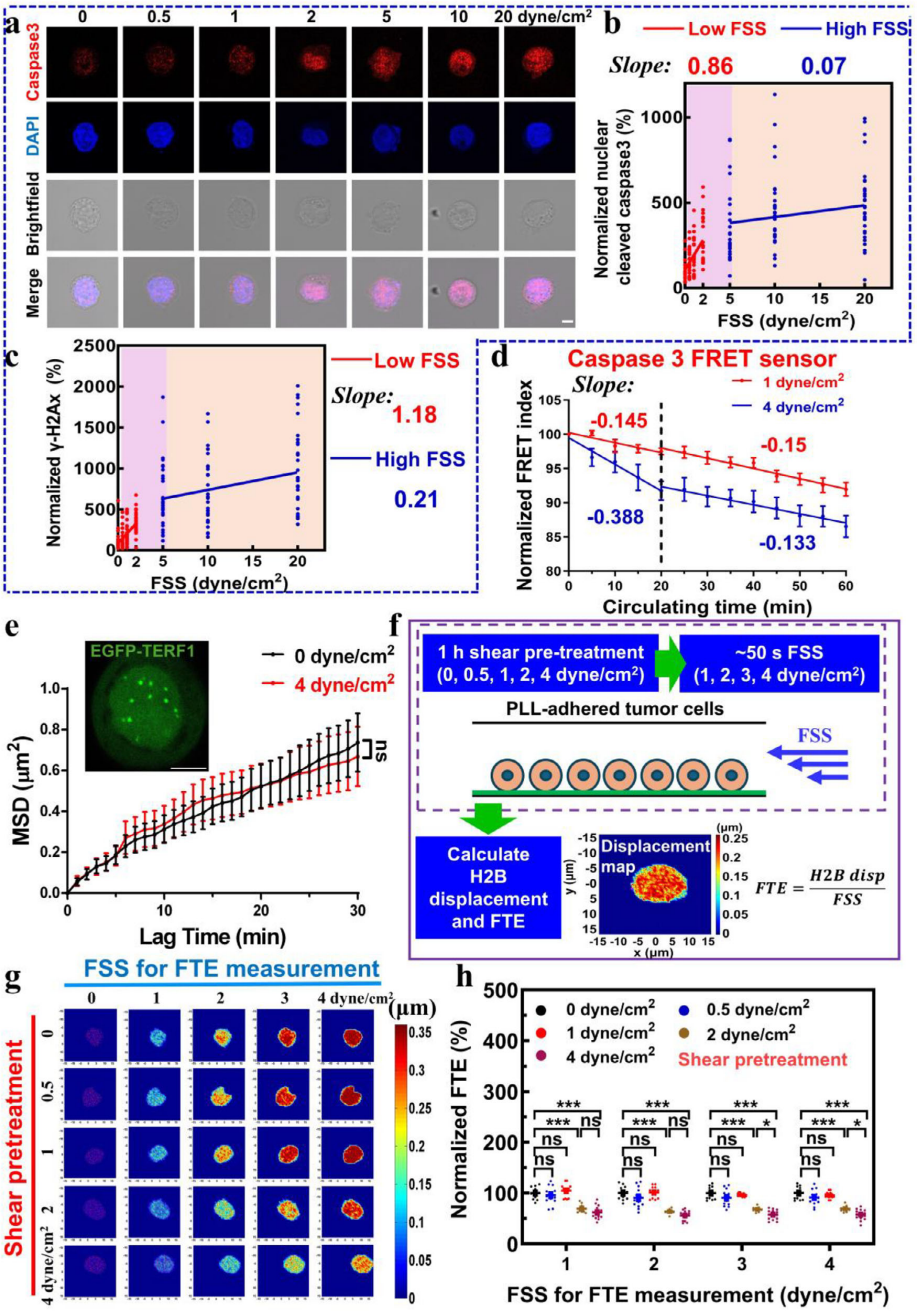
Non‐adherent tumor cells exhibit reduced mechanoresponses and force transmission to increasing FSS. Representative immunofluorescence images (a) and quantification (b) of nuclear cleaved caspase 3 after the treatment under varying levels of FSS. Non‐adherent MCF‐7 cells were circulated under 0, 0.5, 1, 2, 5, 10, and 20 dyne/cm^2^ FSS for an hour in the in vitro microfluidic system. The red and blue lines represent the linear regression of data within the ranges of low and high FSS. n = 24, 26, 28, 22, 24, 31, and 31 cells for 0, 0.5, 1, 2, 5, 10, and 20 dyne/cm^2^, respectively. Scale bar, 5 µm. (c) Quantification of γ‐H2Ax after the treatment under varying levels of FSS. Non‐adherent MCF‐7 cells were treated similarly as in (a). n = 30, 37, 33, 31, 30, 32, and 33 cells for 0, 0.5, 1, 2, 5, 10, and 20 dyne/cm^2^, respectively. (d) Quantification of caspase 3 FRET index under 1 or 4 dyne/cm^2^ FSS. MCF‐7 cells were transfected with caspase 3 FRET biosensor, trypsinized and further attached to PLL‐coated microfluidic chip. These cells were then treated under 1 or 4 dyne/cm^2^ FSS. n = 22 and 20 cells for 1 and 4 dyne/cm^2^ FSS, respectively. (e) Quantification of spontaneous mean square displacement (MSD) of EGFP‐TERF1 after the treatment of 0 and 4 dyne/cm^2^ FSS. Non‐adherent MCF‐7 cells were transfected with EGFP‐TERF1, attached to PLL‐coated microfluid chip and subjected to 0 and 4 dyne/cm^2^ FSS for an hour. MSD of EGFP‐TERF1 was measured after the FSS treatment. n = 20 and 16 cells for 0 and 4 dyne/cm^2^ FSS, respectively. Scale bar, 5 µm. (f) Schematic of the measurement of force transmission efficiency (FTE) under FSS. (g) Representative displacement maps of histone 2B (H2B) of non‐adherent tumor cells after the pre‐treatment of 0, 0.5, 1, 2, and 4 dyne/cm^2^ FSS for an hour. (h) Normalized FTE of non‐adherent tumor cells after the pre‐treatment of 0, 0.5, 1, 2, and 4 dyne/cm^2^ FSS for an hour. n = 11, 13, 14, 9, and 14 cells for 0, 0.5, 1, 2, and 4 dyne/cm^2^ FSS pre‐treatment, respectively. Data were summarized from 3 biological repeats and presented as mean ± SEM. Data acquired from the circulatory system were highlighted with blue dash line. Mann–Whitney U‐test was adopted for comparison in (e). Kruskal–Wallis one‐way ANOVA followed by Mann–Whitney U‐test with Bonferroni correction was adopted for comparison in (h). ns, no significant difference; *, p < 0.05; ***, p < 0.001.

To generate overt metastases, disseminated tumor cells must survive all the rate‐limiting factors, including FSS during hematogenous metastasis. Our previous studies have shown that high FSS (8–20 dyne/cm^2^ in capillary and artery) efficiently eliminates the majority of CTCs in a magnitude‐dependent manner [23]. However, a small subpopulation still persists even under high FSS [23], implicating the possibility that unknown mechanisms enable the resilience to and protect them from high shear‐mediated death. To test this idea, we adopted non‐adherent breast cancer cells as a model to study their responses to varying levels of FSS. The half lifetime of CTCs in vivo is ∼2 h [24]. To mimic such shearing milieu in blood circulation, non‐adherent tumor cells were circulated under 0.5 to 20 dyne/cm^2^ FSS for 1 h in a previously developed microfluidic system [23]. All the FSS reported in this study represented wall shear stress. Since the majority of tumor cells are viable under such shearing milieu [23], the apoptosis‐associated markers but not viability, such as cell death‐related nuclear cleaved caspase 3 and DNA damage marker γ‐H2AX, were adopted to represent the survival responses. As expected, the level of nuclear cleaved caspase 3 increased in a force‐dependent manner (Figure 1a,b). Remarkably, this response increased much more rapidly (the slope of the fitted line: 0.86 vs 0.07 arbitrary unit or AU per dyne/cm^2^) under low FSS (0.5–2 dyne/cm^2^) compared with high FSS (5–20 dyne/cm^2^) (Figure 1b), possibly suggesting the reduced mechanoresponses along with the increase of FSS. To confirm this finding, the alteration of γ‐H2AX was characterized. The slope of γ‐H2AX response under low FSS was much steeper than that under high FSS (Figure 1c; the slope of the fitted line: 1.18 vs 0.21 AU per dyne/cm^2^), reminiscent of the findings of nuclear cleaved caspase 3.

The actual shear stress experienced by individual tumor cells varies and depends on their radial positions in the tubing [23, 25]. To minimize the stress variation and for the convenience of dynamic tracking, non‐adherent tumor cells were attached to the microfluidic chip coated with poly‐L‐lysine (PLL) through non‐specific interaction (Figure S1a), in which the stress exerted on PLL‐attached tumor cells was close to the adopted wall shear stress. Therefore, the ranges of low and high FSS were different between the circulatory system and PLL‐coated microfluidic chips (Figure S1a,b). Similar to non‐adherent tumor cells in the tubing, PLL‐attached cells exhibited similar morphology (round shape and low spreading area) and vinculin expression (Figure S1c–f), and displayed much faster increase in both nuclear cleaved caspase 3 (1.38 vs 0.19 AU per dyne/cm^2^) and γ‐H2AX (1.63 vs 0.15 AU per dyne/cm^2^) under low FSS (0.25–1 dyne/cm^2^) compared to high FSS (2–8 dyne/cm^2^) (Figure S1g,h). Further, the dynamic alterations of nuclear cleaved caspase 3 in individual PLL‐attached cells under different levels of FSS were characterized using pMXs‐IP‐SCAT3.2 fluorescence resonance energy transfer (FRET) based caspase 3 biosensor [26], in which high and low FRET index represented low and high caspase 3 activity, respectively. Starting from 20 min after shearing, the increase rate of nuclear cleaved caspase 3 tended to decline only under high but not low FSS (Figure 1d), which was further supported by the mechanoadaptive responses of caspase 3 using immunofluorescent staining (Figure S1i,j). These results further support the reduced mechanoresponses of non‐adherent tumor cells under high FSS.

The mechanosensitivity largely depends on the transmission of the externally exerted force from cell membrane to chromatin [21], which influences chromatin structure, accessibility and gene transcription. However, it is technically challenging to directly measure the force within chromatin. The microrheology analysis showed that high FSS had no obvious influence on the mean square displacement (MSD) of EGFP‐labeled TERF1 (telomeric repeat‐binding factor 1) (Figure 1e) [27]. Furthermore, the loss modulus and dynamic viscosity calculated from the MSD of TERF1 through local power‐law approximation remained nearly unchanged before and after the treatment of shear stress (Figure S2a,b) [28], suggesting that the global chromatin mechanics remained unchanged and that delayed viscoelastic relaxation did not play a major role in H2B displacement. Therefore, we adopted chromatin displacement as an alternative indicator to represent the amount of force that was transmitted into chromatin. To measure this displacement, the chromatins of tumor cells were labeled with H2B‐RFP. These cells were then attached to the PLL‐coated microfluidic chip and treated under varying levels of FSS. The H2B displacement was calculated from fifty images during the course of FSS application according to a reported method by computing the maximum H2B displacements between each two images [29]. It is known that cell detachment mediates substantial chromatin remodeling and deformation, which was recapitulated by significant H2B displacement with time (Figure S2c). As expected, larger FSS applied on the cell surface led to higher H2B displacement (Figure S2d,e), indicating the increase in the total amount of force transmitted into chromatin when the chromatin mechanics remained unchanged. To quantify how effective the mechanotransduction process was, we defined a parameter namely FTE as the ratio of resultant H2B displacement and the level of FSS applied on the surface of shear‐pretreated cells, which was then normalized to the FTE of tumor cells without shear pretreatment (normalized FTE; Figure S2f). The resultant alteration of mechanoresponses was evaluated by the normalized FTE when a short‐term FSS (<60 s) was applied (Figure 1f; see **Materials and Methods**). Notably, the pre‐treatment with high (2 dyne/cm^2^) but not low FSS (0.5 and 1 dyne/cm^2^) significantly reduced the normalized FTE by 35% compared to the untreated control. The increase of the pretreatment FSS from 2 to 4 dyne/cm^2^ further decreased the FTE (Figure 1g,h). Tumor cells without shear pretreatment exhibited similar FTE under different levels of FSS (Figure S2g,h). The unchanged FTE under low FSS and reduced FTE under high FSS were independent of nuclear and cell size (Figure S3). These results implicate the reduction of force transmission from cell surface to chromatin after high shear pretreatment.

In summary, all these findings suggest that the pretreatment with high FSS reduces force transmission in non‐adherent tumor cells, which could decrease the mechanoresponses of non‐adherent tumor cells under FSS.

### 2.2 Re‐Localization of Activated Myosin to Cytoplasm Under High FSS Attenuates Force Transmission

**FIGURE 2:**
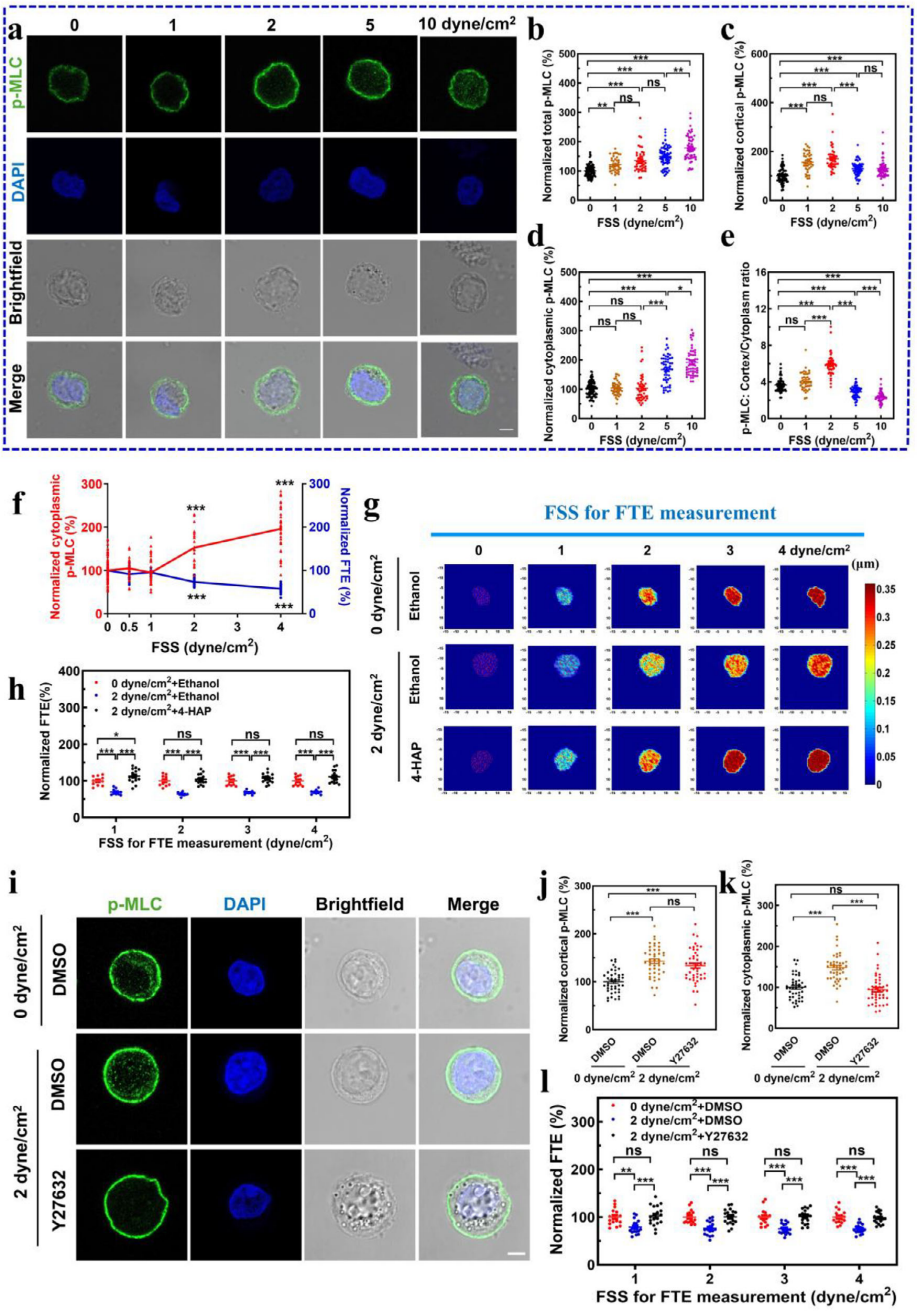
The re‐localization of activated myosin to cytoplasm but not cortex under high FSS attenuates force transmission and mechanoresponses. Representative immunofluorescence images (a) and quantification of phosphorylated myosin light chain (p‐MLC) in whole cells (b), cortex (c) and cytoplasm (d), and of p‐MLC cortical/cytoplasmic ratio (e) after the treatment of 0, 1, 2, 5, and 10 dyne/cm^2^ FSS for an hour. Non‐adherent tumor cells were treated similarly as in Figure 1a. n = 89, 40, 45, 51, and 54 cells for 0, 1, 2, 5, and 10 dyne/cm^2^, respectively. Scale bar, 5 µm. (f) Correlation between normalized FTE and cytoplasmic p‐MLC under varying levels of FSS. n = 105, 29, 39, 22, and 41 cells for cytoplasmic p‐MLC under 0, 0.5, 1, 2, and 4 dyne/cm^2^, respectively. n = 12, 13, 14, 19, and 14 cells for FTE under 0, 0.5, 1, 2, and 4 dyne/cm^2^, respectively. Representative H2B displacement map (g) and normalized FTE (h) after the pre‐treatment under 0 and 2 dyne/cm^2^ FSS with or without 4‐HAP for an hour. Non‐adherent tumor cells were pre‐treated with 4‐HAP under 0 and 2 dyne/cm^2^ FSS for an hour, and then subjected to short‐term FSS (1–4 dyne/cm^2^) for FTE measurement. n = 11, 9, and 15 cells for 0 dyne/cm^2^ with DMSO, 2 dyne/cm^2^ with DMSO, and 2 dyne/cm^2^ with 4‐HAP, respectively. Representative immunofluorescence images (i) and quantification of p‐MLC in the cortex (j) and cytoplasm (k) after the treatment of FSS and Y27632. Non‐adherent tumor cells were treated under 0 and 2 dyne/cm^2^ FSS for an hour and then with Y27632 for 10 min. The subcellular distribution of p‐MLC was measured. n = 43, 44, and 45 cells for 0 dyne/cm^2^ with DMSO, 2 dyne/cm^2^ with DMSO and 2 dyne/cm^2^ with Y27632, respectively. Scale bar, 5 µm. (l) Normalized FTE of non‐adherent tumor cells after the pre‐treatment in (i). n = 17, 17, and 19 cells for 0 dyne/cm^2^ with DMSO, 2 dyne/cm^2^ with DMSO, and 2 dyne/cm^2^ with Y27632, respectively. Data were summarized from 3 biological repeats and presented as mean ± SEM. Data acquired from the circulatory system were highlighted with blue dash line. The one‐way ANOVA followed by Tukey's post‐hoc test was adopted for comparison in (j). Kruskal–Wallis one‐way ANOVA followed by Mann–Whitney U‐test with Bonferroni correction was adopted for comparison in (b–e), (f), (h), and (k,l). ns, no significant difference; *, p < 0.05; **, p < 0.01, and ***, p < 0.001.

We next explored how the pretreatment under high FSS reduced force transmission. Mechanosensitive proteins on cell surface perceive and transmit external forces into chromatin through cytoskeleton and the associated proteins, of which non‐muscle myosin II plays a crucial role [14]. We then tested the influence of varying FSS on myosin activity. The total amount of phosphorylated myosin light chain (p‐MLC), representative of activated non‐muscle myosin II, progressively elevated along with the increase of FSS (Figure 2a,b). Recent work shows rapid myosin redistribution from cytoplasm to cortex under spatial confinement, triggering the increase of contractility and enabling the passage through the confined microenvironment [20], implicating distinct functions of myosin in different subcellular regions. We further analyzed the subcellular distribution of activated myosin in cytoplasm and cortex, which were compartmented based on a reported method [30]. The cortex thickness of non‐adherent breast cancer cells was ∼450 nm, which did not change after shear treatment (Figure S2i). Surprisingly, after the treatment under low FSS for 1 h, the increased p‐MLC was predominantly localized in cell cortex and the p‐MLC cortex/cytoplasm ratio gradually increased along with FSS (Figure 2c–e). Low FSS had minimal influence on cytoplasmic p‐MLC (Figure 2d). In contrast, under high FSS, p‐MLC was re‐localized to cytoplasm and the p‐MLC cortex/cytoplasm ratio progressively decreased (Figure 2c–e). Similar findings were also observed in PLL‐attached tumor cells (Figure S4a–e). With no shear treatment, PLL‐mediated attachment did not influence myosin II activity and subcellular localization (Figure S4f–h). These results suggest that under low FSS, there were subtle changes in both cytoplasmic p‐MLC and FTE (Figure 2f), while cortical p‐MLC significantly increased (Figure S4i). In comparison, under high FSS, cytoplasmic but not cortical p‐MLC and FTE progressively increased and decreased, respectively (Figure 2f). Further, cytoplasmic p‐MLC began to increase at 20 min after shearing when FTE started to decrease (Figure S4j–l). When non‐adherent tumor cells were circulated in the tubing of the microfluidic system, cytoplasmic p‐MLC and FTE progressively increased and decreased, respectively, and reached plateaus at 2 h after high shear treatment (Figure S5). These results suggest the correlation between cytoplasmic myosin and force transmission under high FSS.

We then explored the potential roles of cytoplasmic and cortical myosin in force transmission and mechanosensitivity. Consistent with previous reports [31], 4‐hydroxyacetophenone (4‐HAP), an activator of cortical myosin, enhanced p‐MLC only in cortex but not cytoplasm in the absence of FSS (Figure S6a–e). Interestingly, under high FSS (4 dyne/cm^2^), 4‐HAP redistributed activated myosin from cytoplasm to cortex while keeping the total p‐MLC constant (Figure S6a–e). Importantly, 4‐HAP restored the reduced FTE after high FSS pretreatment to the level of control cells (Figures 2g,h and S6f,g), indicating that myosin subcellular distribution might be essential in regulating force transmission. To selectively modulate cytoplasmic and cortical myosin, Y27632, a Rho‐associated protein kinase (ROCK) inhibitor, was adopted to treat tumor cells for 10 min after the pre‐treatment under high FSS (2 dyne/cm^2^), which significantly reduced cytoplasmic but not cortical p‐MLC and rescued the FTE to the control level (Figure 2i–l). When tumor cells were pre‐treated with both high FSS and Y27632 or with high FSS and the siRNAs of myosin IIB or IIA for 1 h, myosin activity in both cortex and cytoplasm was reduced and the FTE was also restored to the level of control cells or even higher (Figures S6h–k and S7). In contrast, Blebbistatin (Bleb), an inhibitor of myosin II ATPase activity, reduced cortical not cytoplasmic myosin activity but failed to rescue the reduced FTE (Figure S8a–d). In addition, ML7, an inhibitor of myosin light chain kinase (MLCK), specifically suppressed high shear‐induced cortical but not cytoplasmic p‐MLC, but had no effect on force transmission (Figure S8e–h). Similar findings were observed when *MLCK* was silenced using siRNAs (Figure S8i–k). All the pharmacologic treatments did not influence cell viability, excluding the potential role of drug cytotoxicity in force transmission (Figure S8l). All these findings conclude that high shear‐induced cytoplasmic but not cortical p‐MLC reduces force transmission.

Further, we examined whether the effect of cytoplasmic myosin activity on force transmission and mechanosensitivity depended on high FSS. Pre‐treatment of non‐adherent tumor cells with Y27632 but no FSS decreased cytoplasmic but not cortical p‐MLC and notably elevated FTE (Figure S9a–c). Silencing myosin IIB inhibited cytoplasmic p‐MLC but spared cortical myosin, while inhibiting myosin IIA suppressed both cortical and cytoplasmic myosin activity (Figure S7a–d, h–k). The inhibition of myosin IIA or IIB promoted force transmission (Figure S7g,n). In addition, in the absence of shear pre‐treatment, calyculin A, a protein phosphatase inhibitor, upregulated myosin II activity in cytoplasm but not cortex and attenuated force transmission (Figure S9d–f). These results suggest that the regulation of force transmission by cytoplasmic myosin is independent of FSS and could be an intrinsic property.

To further validate the role of cytoplasmic myosin in force transmission, FRET Nesprin‐based tension sensor was employed to measure the force transmitted from actin to Nesprin in the linker of nucleoskeleton and cytoskeleton (LINC) complex [32]. The sensor was verified in adherent cells, where inhibition of actomyosin contractility increased the FRET index, representing lower tension across LINC complex, consistent with previous findings [32] (Figure S10a,b). Varying shear forces progressively decreased the FRET index, indicating that increased level of force was transmitted from cytoskeleton to LINC complex (Figure S10c). Silencing myosin IIB inhibited cytoplasmic p‐MLC (Figure S7d) and reduced the FRET index under FSS, suggesting the elevated force transmission (Figure S10d–g), consistent with the increase of FTE (Figure S7g).

Except myosin, cytoskeletal elements, such as F‐actin and microtubule, are also essential in contractility generation and force transmission. FSS enhanced F‐actin in both cortex and cytoplasm (Figure S11a–c). Inhibition of actin polymerization with cytochalasin D both in control cells (Figure S11d) and in non‐adherent tumor cells under high shear pretreatment (Figure S11e) decreased FTE, suggesting a distinct role of F‐actin in force transmission compared with cytoplasmic myosin. Y27632 treatment had no obvious influence on shear‐mediated actin polymerization (Figure S11f,g), excluding the potential contribution of F‐actin to myosin‐dependent force transmission. Furthermore, shear treatment also upregulated α‐tubulin (Figure S11h,i). However, inhibition of microtubule polymerization by nocodazole seemed not to disrupt force transmission (Figure S11j). Recent studies show that DNA damage mediates nuclear softening, which promotes DNA repair and protects cells from further accumulation of DNA damage [33]. FSS induced considerable level of DNA damage in non‐adherent tumor cells (Figure 1c and Figure S1h), which may potentially affect force transmission and survival. To explore this possibility, we utilized the short‐term treatment of tumor cells with low dose of doxorubicin to induce DNA damage, which did not impact their survival (Figure S12a–d). Interestingly, the induced DNA damage did not affect subcellular distribution of p‐MLC or FTE (Figure S12e–h). Furthermore, cell culture in low serum medium, which induces cell cycle arrest, did not influence cytoplasmic myosin II activation and force transmission under FSS (Figure S12i–l). In addition, inhibition of E‐cadherin did not influence myosin subcellular localization, force transmission, or cell viability under shear stress (Figure S13), suggesting that cell‐cell adhesion‐mediated signaling pathway did not play major roles in the response of non‐adherent tumor cells to FSS. These results exclude the possible roles of shear‐induced DNA damage, cell cycle arrest, and cell‐cell adhesion in force transmission.

### 2.3 Shear‐Induced Cytoplasmic Myosin Disrupts the Binding With Actin

**FIGURE 3:**
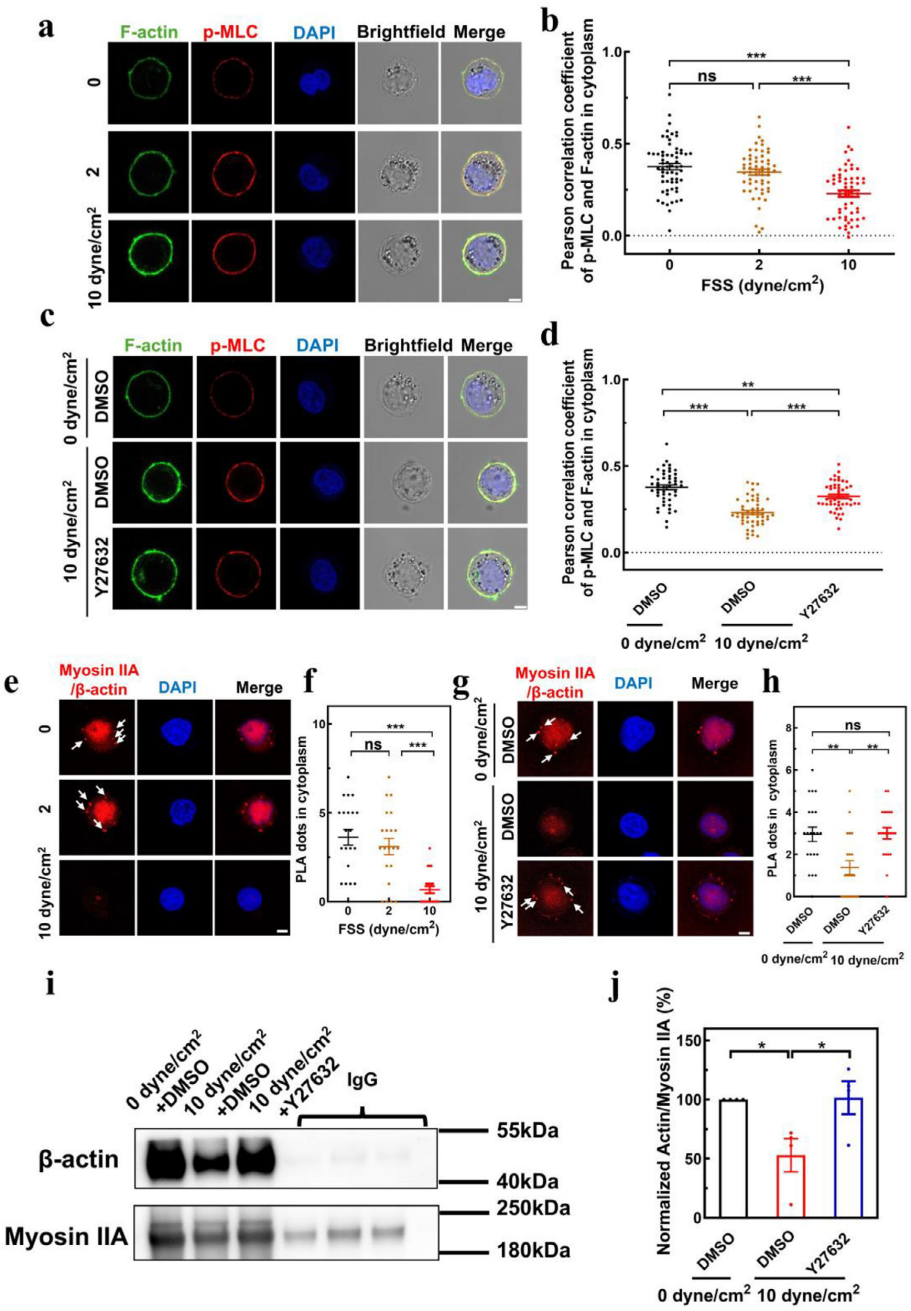
Shear‐induced re‐localization of activated myosin to cytoplasm disrupts the binding of myosin with actin. Representative immunofluorescence images (a) and the co‐localization (b) of p‐MLC with F‐actin in non‐adherent tumor cells after the treatment under 0, 2, and 10 dyne/cm^2^ FSS for an hour. The co‐localization of p‐MLC with F‐actin in cytoplasm was analyzed by calculating the Pearson correlation coefficient. n = 62, 58, and 59 cells for 0, 2, and 10 dyne/cm^2^ FSS, respectively. Scale bar, 5 µm. Representative immunofluorescence images (c) and the co‐localization (d) of p‐MLC with F‐actin after the treatment of FSS and Y27632. Non‐adherent tumor cells were treated under 0 and 10 dyne/cm^2^ FSS for an hour and then with Y27632 for 10 min. The co‐localization of p‐MLC with F‐actin in cytoplasm was measured. n = 48, 49, and 48 cells for 0 dyne/cm^2^ with DMSO, 10 dyne/cm^2^ with DMSO and 10 dyne/cm^2^ with Y27632, respectively. Scale bar, 5 µm. Representative proximity ligation assay (PLA) images (e) and the quantification (f) of PLA dots in cytoplasm after the treatment under 0, 2, and 10 dyne/cm^2^ FSS for an hour. The interaction between myosin IIA and β‐actin was detected by PLA assay. White arrows indicated typical PLA dots. n = 21 cells for each condition. Scale bar, 5 µm. Representative PLA images (g) and the quantification (h) of PLA dots in cytoplasm after the treatment of FSS and Y27632. Non‐adherent tumor cells were treated under 0 and 10 dyne/cm^2^ FSS for an hour and then with Y27632 for 10 min. The interaction between myosin IIA and β‐actin was detected. White arrows indicated typical PLA dots. n = 21 cells for each condition. Scale bar, 5 µm. Representative images (i) and quantification (j) of co‐immunoprecipitation between myosin IIA and β‐actin after the treatment of FSS and Y27632. Non‐adherent tumor cells were treated under 0 and 10 dyne/cm^2^ FSS for an hour and then with Y27632 for 10 min. Data were summarized from 4 biological repeats and presented as mean ± SEM. The one‐way ANOVA followed by Tukey's post‐hoc test was adopted for comparison in (b), (d), (f), (h), and (j). ns, no significant difference; *, p < 0.05; **, p < 0.01, and ***, p < 0.001.

We next addressed how activated myosin in cytoplasm reduced force transmission. For adherent cells, external mechanical forces are usually propagated from cell surface into chromatin via cytoskeleton [34]. The association of F‐actin with myosin stabilizes the cytoskeletal network that serves as the structural basis for force transmission [14]. Therefore, it was possible that shear‐induced cytoplasmic myosin might influence the interaction of F‐actin with myosin, which further impacted force transmission. After shear pretreatment, the colocalization between p‐MLC and F‐actin in cytoplasm was assessed by calculating the Pearson correlation coefficient. Remarkably, high FSS significantly decreased this colocalization, which could be rescued by the inhibition of cytoplasmic myosin with Y‐27632 (Figure 3a–d and Figure S14a–d). In contrast, low FSS had no obvious influence on the colocalization between p‐MLC and F‐actin (Figure 3a,b and Figure S14a,b). Further, proximity ligation assay (PLA) was conducted to examine the physical interaction between these two proteins. The results showed that high FSS reduced the binding of myosin IIA with actin in cytoplasm, which was restored to the similar level of control cells by the inhibition of cytoplasmic but not cortical myosin (Figure 3e–h and Figure S14e,f). This result was further supported by co‐immunoprecipitation analysis (Figure 3i,j). It was noteworthy that key linkers of lamina‐chromatin such as HP1 and BAF remained unchanged under shear stress, indicating that the lamina‐chromatin linkage might not contribute to the reduced force transmission under high FSS (Figure S15) [35]. Therefore, these findings suggest that high but not low FSS mediates the redistribution of activated myosin into cytoplasm, which disrupts the binding of actin with myosin II, thereby leading to inefficient force transmission [18].

**FIGURE 4:**
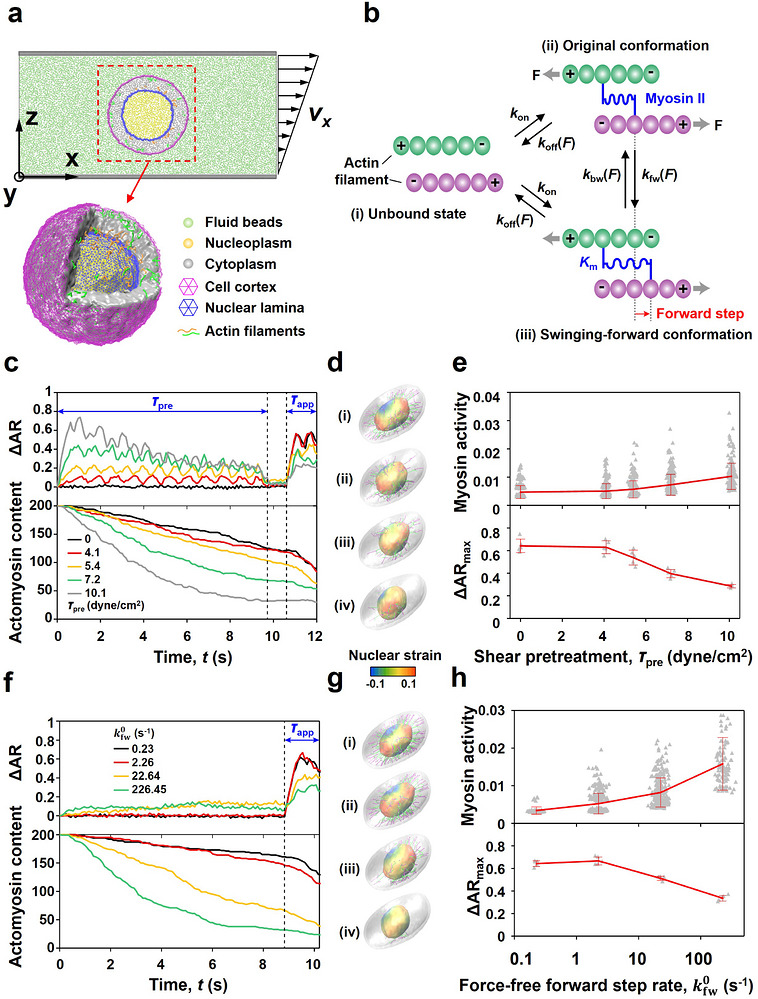
Myosin II is detached from actin to reduce force transmission in the dissipative particle dynamics (DPD) model of non‐adherent tumor cells under FSS. (a) Schematic of whole‐cell level DPD model of non‐adherent tumor cells under FSS. The DPD model of a non‐adherent tumor cell consisted of cell cortex, actin filament, nuclear lamina, cytoplasm, and nucleoplasm. Shear flow with a uniform velocity gradient $\frac{dv_{x}}{dz}$ (i.e., shear rate) was generated to apply FSS on the non‐adherent tumor cell. (b) A mechano‐chemical model for capturing the actin‐myosin kinetics. Myosin cycle within three conformational states: two bound states (original conformation and swinging‐forward conformation) and one unbound state, with these transitions being modeled by force‐dependent first‐order reactions. For visual simplicity, only 6 beads of each actin filament were drawn. (c) Effects of pretreatment FSS τ_pre_ on nuclear deformation (top) and actomyosin content (bottom). Nuclear deformation and actomyosin content were quantified by the variation of aspect ratio (ΔAR) of the ellipsoid‐fitted nucleus and the number of actin‐myosin bonds, respectively. Shear stress application scheme: pretreatment FSS (τ = τ_pre_) during 0 s < *t* ≤ 9.7 s; relaxation (τ = 0 dyne/cm^2^) during 9.7 s < *t* < 10.6 s; and FSS (τ = τ_app_ = 10.1 dyne/cm^2^) during 10.6 s ≤ *t* ≤ 12.0 s. (d) Snapshots of non‐adherent tumor cells after the pre‐treatment under different shear stresses at *t* = 11.04 s. (i): τ_pre_ = 0 dyne/cm^2^; (ii): τ_pre_ = 5.4 dyne/cm^2^; (iii): τ_pre_ = 7.2 dyne/cm^2^; (iv): τ_pre_ = 10.1 dyne/cm^2^. Color denoted nuclear strain. (e) Effects of pretreatment FSS τ_pre_ on myosin activity (top) and maximum nuclear aspect ratio (ΔAR_max_) during shear stress application (bottom). Myosin activity was quantified as the ratio of the number of forward reactions to the number of actin‐myosin bonds in a time interval. 5 independent runs, with each run involving statistics over 4.42 s < *t* < 8.83 s. (f) Effects of myosin force‐free forward rate $k_{\mathrm{fw}}^{0}$ on nucleus ΔAR (top) and actomyosin content (bottom). Shear stress application scheme: varying $k_{\mathrm{fw}}^{0}\;$ without shear stress application during 0 s < *t* < 8.8 s; FSS (τ = τ_app_ = 10.1 dyne/cm^2^) during 8.8 s ≤ *t* ≤ 10.2 s. (g) Snapshots of non‐adherent tumor cells with different $k_{\mathrm{fw}}^{0}$ at *t* = 9.27 s. (i): $k_{\mathrm{fw}}^{0}=0.23s^{-1}$; (ii): $k_{\mathrm{fw}}^{0}=2.26s^{-1}$; (iii): $k_{\mathrm{fw}}^{0}=22.64s^{-1}$; (iv): $k_{\mathrm{fw}}^{0}=226.45s^{-1}$. Color denoted nuclear strain. (h) Effects of myosin force‐free forward rate $k_{\mathrm{fw}}^{0}$ on myosin activity (top) and maximum nuclear aspect ratio (ΔAR_max_) during shear stress application (bottom). 5 independent runs, with each run involving statistics over 4.42 s < *t* < 8.83 s. Data were presented as mean ± SD.

To explore the mechanisms underlying the reduction of force transmission into chromatin under high FSS (Figures 1g and 3), we hypothesized in our simulations that high but not low FSS caused the stretch of actin‐ and myosin‐based cytoskeleton in the direction of flow, which led to the mechanosensitive detachment of myosin from actin, known as cytoskeletal fluidization [18, 36, 37], ultimately attenuating the transmission of forces into chromatin. To test this hypothesis, we developed a dissipative particle dynamics (DPD) model using a mesoscopic particle‐based coarse‐graining method to simulate the dynamics of non‐adherent tumor cells under FSS. Based on the DPD model of red blood cells [38, 39], we constructed a non‐adherent tumor cell model at the whole‐cell level by explicitly considering the tension‐dependent kinetics of the major force‐bearing cellular components, such as myosin II and actin filaments (Figure 4a,b; see more details in the Materials and Methods).

Similar to the experimental shearing conditions, the cells were first subjected to the pretreatment under FSS of different magnitudes ($\tau =\tau_{\mathrm{pre}},t=0∼9.7\;s$). After a brief relaxation ($\tau =0\;\mathrm{dyne}/cm^{2},t=9.7∼10.6\;s$), FSS with short duration ($\tau =\tau_{\mathrm{app}},t=10.6∼12\;s$) was exerted to measure the FTE of shear‐pretreated cells (Figure 4c). Actomyosin content (i.e., the number of actin‐myosin bonds) was adopted to evaluate the extent of the myosin detachment from actin filament, and the nuclear deformation was represented by the variation of aspect ratio (ΔAR) of the ellipsoid‐fitted nucleus (i.e., the ratio of the longest axis to the shortest axis). The higher the pretreatment FSS τ_pre_, the larger the initial ΔAR of the nucleus, which then led to a rapid decrease in actomyosin content (Figure 4c,d). The detachment of myosin from cytoskeleton induced by the pretreatment shear stress protected the cells from drastic nuclear deformation under the subsequent FSS application (see the grey line in Figure 4c).

We then analyzed myosin activity during FSS pretreatment (τ_pre_) as well as the maximum aspect ratio (ΔAR_max_) of nucleus under the subsequent FSS (τ_app_). Myosin activity was quantified as the ratio of the number of forward reactions to the number of actin‐myosin bonds within a time interval. Our modeling results showed that myosin activity increased while nuclear ΔAR_max_ decreased with increasing pretreatment FSS (τ_pre_; Figure 4e), which recapitulated the reduction of nuclear deformation in the experimental results (Figure 1f,g). These results suggest that FSS pretreatment leads to force‐dependent myosin detachment from actin, which also elevates myosin activity by shifting the equilibrium of actin‐myosin reactions.

To further explore the effects of myosin activity, we set different $k_{\mathrm{fw}}^{0}$ so that myosin had different movement capacity toward the plus end of the actin filament (i.e., actomyosin contractility). A larger $k_{\mathrm{fw}}^{0}$ represented a higher myosin activity, as well as stronger contractility. We tested the equilibrium kinetics of cells at different $k_{\mathrm{fw}}^{0}$ (t=0∼8.8s) and then applied FSS τ_app_ (t=8.8∼10.2s) (Figure 4f). Compared with Figure 4c–e, which varied the pretreatment FSS τ_pre_, increasing myosin activity had similar effects on actomyosin content and nuclear deformation (Figure 4f–h), consistent with the experimental results (Figure S9d–f). These results suggest that elevated cytoplasmic myosin activity causes active fluidization of the cytoskeleton to reduce force transmission [36].

Our experimental findings together with the simulation indicate that high FSS increases the tension within the myosin‐actin complex, leading to the mechanosensitive disassembly and ultimately disrupting force transmission to the chromatin.

### 2.4 Lamin A/C‐Mediated Nuclear Mechanosensing Orchestrates Shear‐Induced Calcium Release From ER and Cytoplasmic Myosin Redistribution

**FIGURE 5:**
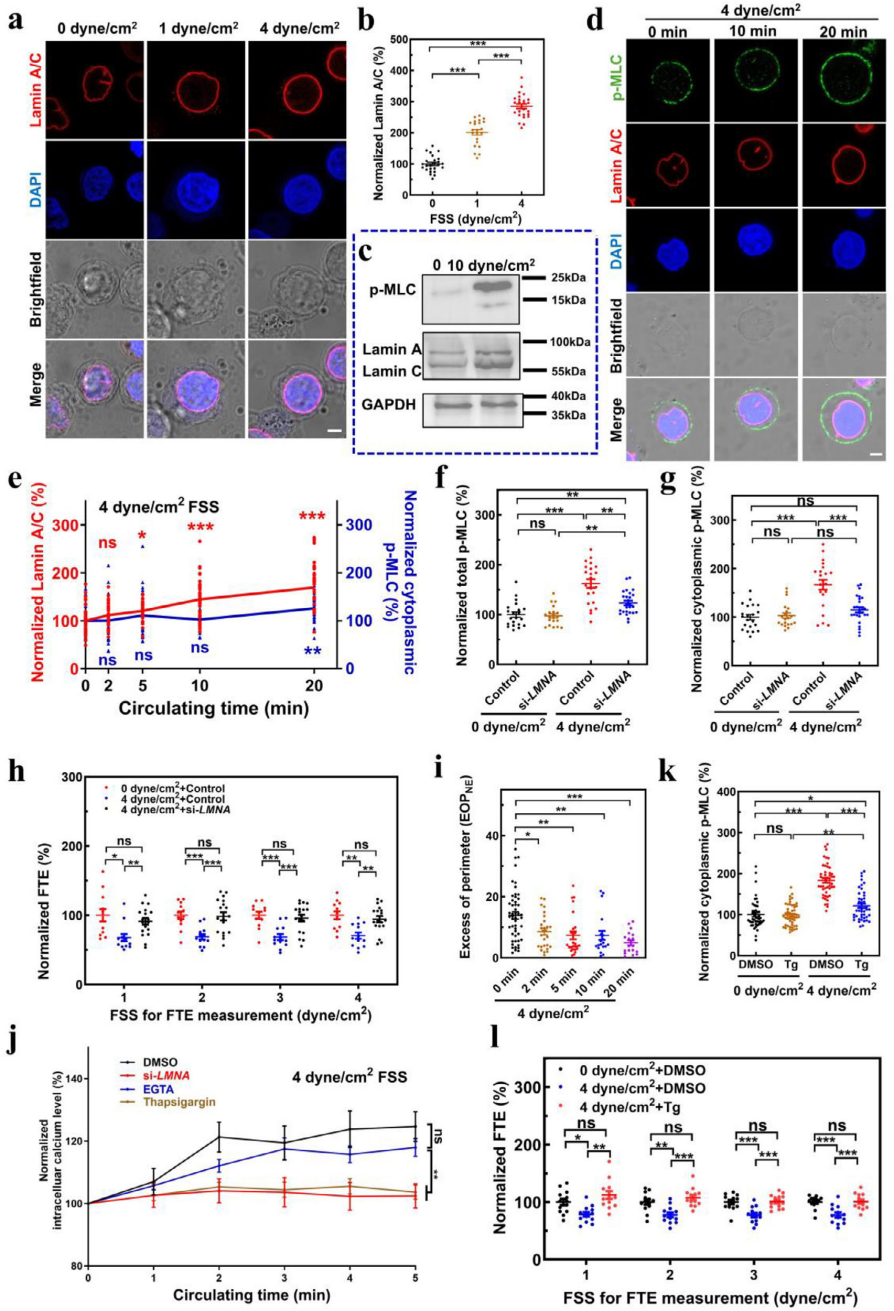
Lamin A/C‐mediated nuclear mechanosensing orchestrates shear‐induced calcium release from ER and cytoplasmic myosin redistribution. Representative immunofluorescence images (a) and quantification (b) of Lamin A/C after the treatment under 0, 1, and 4 dyne/cm^2^ FSS for an hour. n = 25, 22, and 26 cells for 0, 1, and 4 dyne/cm^2^, respectively. Scale bar, 5 µm. (c) Immunoblotting of Lamin A/C and p‐MLC after the treatment under 0 and 10 dyne/cm^2^ FSS for an hour. (d) Representative immunofluorescence images of p‐MLC and Lamin A/C after the treatment under 4 dyne/cm^2^ FSS for 0, 10, and 20 min, respectively. Scale bar, 5 µm. (e) The dynamic changes of cytoplasmic p‐MLC and Lamin A/C under FSS. n = 40, 27, 26, 40, and 40 cells for 0, 2, 5, 10, and 20 min under 4 dyne/cm^2^ FSS, respectively. The levels of p‐MLC in whole cells (f) and cytoplasm (g) of non‐adherent tumor cells after shear treatment and Lamin A/C inhibition. Non‐adherent tumor cells were transfected with Lamin A/C siRNA (si‐*LMNA*) prior to the treatment under FSS. n = 21, 20, 22, and 26 cells for 0 dyne/cm^2^ with Control, 0 dyne/cm^2^ with si‐*LMNA*, 4 dyne/cm^2^ with Control and 4 dyne/cm^2^ with si‐*LMNA*, respectively. (h) Normalized FTE of non‐adherent tumor cells after shear pre‐treatment and Lamin A/C inhibition. Non‐adherent tumor cells were treated similarly as in (f). n = 12, 13, and 18 cells for 0 dyne/cm^2^ with Control, 4 dyne/cm^2^ with Control and 4 dyne/cm^2^ with si‐*LMNA* respectively. (i) Excess of perimeter of nuclear envelop (EOP_NE_) after the treatment of 4 dyne/cm^2^ FSS for 0, 2, 5, 10, and 20 min, respectively. n = 51, 27, 26, 20, and 19 cells for 0, 2, 5, 10, and 20 min, respectively. (j) Intracellular calcium level measured by GCaMP6f calcium sensor under 4 dyne/cm^2^ FSS. Non‐adherent tumor cells were transfected with GCaMP6f calcium sensor, treated with DMSO, si‐*LMNA* or Thapsigargin (Tg), and then subjected to 4 dyne/cm^2^ FSS. Medium was pretreated with 1 mM EGTA to chelate extracellular calcium before FSS. n = 14, 16, 18, and 16 cells for DMSO, si‐*LMNA*, EGTA and Thapsigargin, respectively. Quantification of p‐MLC in cytoplasm (k) after the treatment of FSS and Tg. Non‐adherent tumor cells were pre‐treated with Tg for 2 days and then subjected to 0 or 4 dyne/cm^2^ FSS for an hour. n = 47, 50, 48, and 46 cells for 0 dyne/cm^2^ with DMSO, 0 dyne/cm^2^ with Tg, 4 dyne/cm^2^ with DMSO and 4 dyne/cm^2^ with Tg, respectively. Scale bar, 5 µm. (l) Normalized FTE of non‐adherent tumor cells after shear pre‐treatment and release of ER calcium. Non‐adherent tumor cells were treated similarly as (k). n = 13 cells for each condition. Data were summarized from 3 biological repeats and presented as mean ± SEM. Data acquired from the circulatory system were highlighted with blue dash line. The one‐way ANOVA followed by Tukey's post‐hoc test was adopted for comparison in (b), (e–g) and (j). Kruskal–Wallis one‐way ANOVA followed by Mann–Whitney U‐test with Bonferroni correction was adopted for comparison in (h,i) and (k,l). ns, no significant difference; *, p < 0.05; **, p < 0.01, and ***, p < 0.001.

We next dissected the mechanisms underlying high shear‐induced myosin subcellular redistribution. Cell nucleus has emerged as a mechanosensitive organelle that senses and responds to mechanical stimuli, and FSS induces considerable levels of chromatin displacement (Figure 1g), indicating the potential role of nuclear mechanosensing in the mechanoadaptive responses of non‐adherent tumor cells. We then examined the role of Lamin A/C, an essential component of nuclear envelop [40], in cytoplasmic myosin redistribution and mechanotransduction. FSS upregulated Lamin A/C expression in a force‐dependent manner (Figure 5a–c; Figure S17a,b). Importantly, Lamin A/C started to increase at 5 min after high FSS treatment, preceding the cytoplasmic redistribution of p‐MLC (20 min) (Figure 5d,e; Figures S16a–d and S17). The rapid upregulation of Lamin A/C was mediated by protein degradation rather than protein translation (Figure S18a,b). In addition to protein expression, post‐translational modification of Lamin A/C, such as phosphorylation at Ser22, regulates its degradation, rapidly responds to nuclear deformation [41], and plays a critical role in Lamin A/C activity. Phosphorylated Lamin A/C at Ser22 decreased as early as 2 min after high FSS treatment when its total amount was kept unchanged, thereby decreasing the ratio of Lamin A/C phosphorylation (Figure S16e–h), which represented elevated Lamin A/C activity. Furthermore, phosphorylated Lamin A/C at Ser22 reached a plateau at 10 min, suggesting the rapid response of Lamin A/C dephosphorylation to FSS (Figure S16i,j). Importantly, silencing Lamin A/C reduced high shear‐induced total and cytoplasmic p‐MLC (Figure 5f,g and Figure S18c) and restored the reduced FTE to the level of control cells (Figure 5h). In comparison, the inhibition of Lamin A/C had little influence on cytoplasmic p‐MLC and force transmission of control cells after no (Figures 5f,g; Figure S19f) or low (Figure S19g–j) shear pretreatment. Interestingly, overexpression of Lamin A/C in control cells activated myosin II, enhanced both cortical and cytoplasmic p‐MLC, and decreased FTE without FSS pretreatment (Figures S18c and S19a–e). These results suggest that Lamin A/C‐mediated nuclear mechanosensing regulates high shear‐induced cytoplasmic myosin redistribution and force transmission.

Lamin A/C critically impacts nuclear mechanosensing through the effect on nuclear envelop tension. We thus further compared nuclear envelop tension of non‐adherent tumor cells after shear treatment, represented by EOP_NE_, the excess of perimeter (EOP) of nuclear envelope (NE). Consistent with the change of Lamin A/C dephosphorylation, EOP_NE_ started to decrease at 2 min after high FSS treatment (Figure 5i), indicating the increase of nuclear envelop tension. The increased nuclear envelop tension was further confirmed by higher ER‐flipper TR lifetime after high FSS treatment (Figure S16k,l). The unfolding of nuclear envelop and nuclear tension can regulate calcium release from endoplasmic reticulum (ER) [20], which may further influence p‐MLC intracellular distribution. To test this idea, we employed GCaMP6 calcium sensor to dynamically measure calcium response in non‐adherent tumor cells under FSS. Yoda1, a Piezo1 agonist, effectively elevated the calcium signal (Figure S20a,b), validating the reliability of this sensor. 1‐h high FSS treatment (4 dyne/cm^2^) increased intracellular calcium concentration (Figure S20c,d). Single cell tracking showed that high FSS enhanced intracellular calcium level starting from 2 min after shear treatment (Figure 5j), which was similar to the onset of Lamin A/C dephosphorylation. This calcium response was abolished by silencing Lamin A/C (Figure 5j). Further, inhibition of the ER Ca^2+^ ATPase pump by Thapsigargin (Tg), instead of chelation of extracellular calcium by EGTA, rescued shear‐induced calcium response (Figure 5j and Figure S20e), suggesting that Lamin A/C‐mediated nuclear mechanosensing regulates NE tension and calcium release from ER. We then explored whether calcium release from ER was responsible for cytoplasmic myosin redistribution under FSS. Inhibition of calcium release from ER but not extracellular calcium influx reduced shear‐induced cytoplasmic p‐MLC and rescued the decreased FTE to the similar level of control cells (Figure 5k,l; Figure S20f–k). Consistently, inhibition of calcium release from ER rescued low FSS induced cortical myosin activation but had no influence on cytoplasmic myosin (Figure S21a,b), high shear‐induced Lamin A/C activity or NE tension (Figure S21c–f). Furthermore, Tg treatment rescued the influence of Lamin A/C overexpression on subcellular myosin activation and decreased FTE to the level of control cells (Figure S21g–k). To further confirm the role of nuclear mechanosensing, we impeded the transmission of externally exerted FSS to nucleus through the expression of dominant negative KASH (DN‐KASH) [42]. DN‐KASH significantly decreased FTE of non‐adherent tumor cells (Figure S22a), indicating the impaired nuclear mechanotransduction. Importantly, DN‐KASH abolished the shear‐induced mechano‐responses, including Lamin A/C phosphorylation, EOP, calcium response and cytoplasmic p‐MLC (Figure S22b–j), suggesting the indispensable role of nuclear mechanosensing in non‐adherent tumor cells’ responses to shear stress. Taken together, these results suggest that Lamin A/C‐mediated nuclear mechanosensing triggers calcium release from ER and redistributes activated myosin into cytoplasm, leading to decreased force transmission into chromatin in non‐adherent tumor cells under high shear stress. This conclusion was also applicable to another breast cancer cells MDA‐MB‐231 cells (Figures S23 and 24). Interestingly, MDA‐MB‐231 exhibited higher level of cytoplasmic p‐MLC both with and without shear pretreatment than MCF‐7, leading to lower FTE (Figure S23a–e), which might explain the better survival of MDA‐MB‐231 cells under high FSS [43].

We further elucidated the molecular mechanism underlying shear‐induced subcellular redistribution of activated myosin. Myosin II is activated through ROCK and MLCK [44], which could have distinct functions. Specifically, MLCK and ROCK are responsible for myosin activation and stress fiber formation in cell periphery and centre, respectively [45, 46, 47]. We thus hypothesized that MLCK and ROCK might respond distinctly to different levels of FSS, further leading to the subcellular redistribution of activated myosin. To test this idea, we first examined the effects of varying FSS on the subcellular localization of MLCK and phosphorylated ROCK. FSS gradually elevated the total and cortical amount of MLCK (Figures S25a–e and S26a–e), while there was no difference in cytoplasmic MLCK between low and high FSS (Figures S25d and S26d). In contrast, high but not low FSS enhanced the phosphorylated ROCK in both cytoplasm and cortex (Figures S25f–j and S26f–j). In line with previous findings, silencing Lamin A/C diminished high shear‐induced MLCK and phosphorylated ROCK in both cytoplasm and cortex (Figure S27a–h). The inhibition of calcium release from ER showed no significant influence on MLCK but notably abolished shear‐induced ROCK activation (Figure S27i–p). Further, inhibition of ROCK downregulated p‐MLC mainly in cytoplasm (Figure 2i–k) and rescued FTE under high FSS (Figure 2l), while inhibition of MLCK reduced activated myosin in the cortex but had no effect on force transmission (Figure S8e–k). These results indicate that high FSS exhibits different effects on MLCK and ROCK, which may lead to distinct subcellular redistribution of activated myosin, thereby attenuating force transmission from cell membrane to chromatin.

### 2.5 Inhibition of Cytoplasmic Myosin Re‐Sensitizes CSCs and Primary Tumor Cells to Shear‐Induced Destruction

**FIGURE 6:**
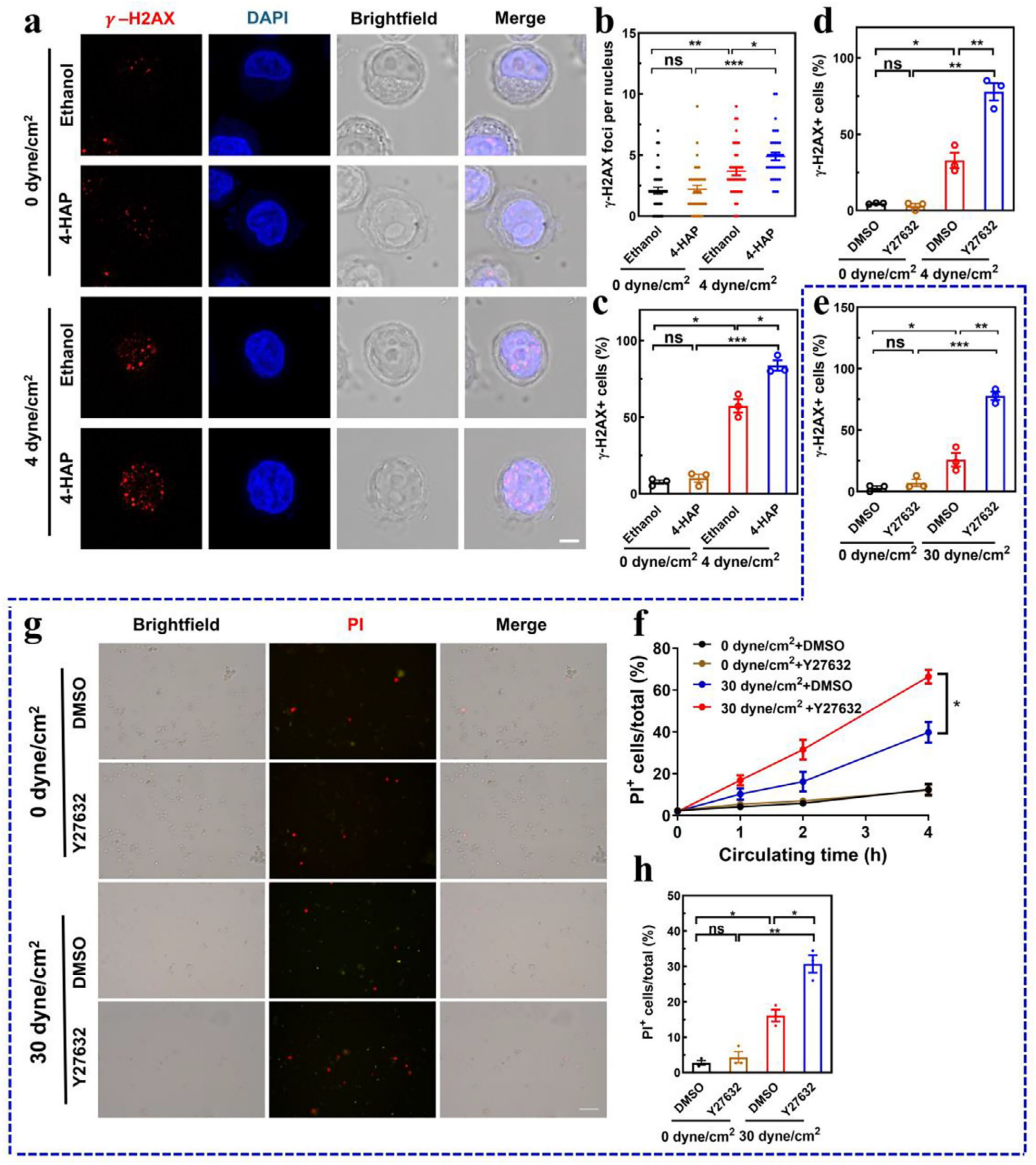
Inhibition of cytoplasmic myosin re‐sensitizes non‐adherent tumor cells and CSCs to shear‐induced DNA damage and apoptosis. Representative immunofluorescence images (a) and quantification of γ‐H2AX foci per nucleus (b) and the percentage of γ‐H2AX+ cells (c) after shear and 4‐HAP treatments. Non‐adherent tumor cells were treated with 4‐HAP under 0 or 4 dyne/cm^2^ FSS for an hour. n = 40, 35, 40, and 36 cells from 3 biological repeats for 0 dyne/cm^2^ with ethanol, 0 dyne/cm^2^ with 4‐HAP, 4 dyne/cm^2^ with ethanol and 4 dyne/cm^2^ with 4‐HAP, respectively. Scale bar, 5 µm. n = 3 biological repeats for each condition in (c). (d) The percentage of γ‐H2AX+ cells after shear and Y27632 treatment. PLL‐attached tumor cells were treated with Y27632 under 0 or 4 dyne/cm^2^ FSS for an hour. n = 3 biological repeats for each condition. (e) The percentage of γ‐H2AX+ cells after shear and Y27632 treatment. Non‐adherent tumor cells were treated with Y27632 under 0 or 30 dyne/cm^2^ FSS in the tubing for 4 h. n = 3 biological repeats for each condition. (f) The percentage of apoptotic cells after shear and pharmacologic treatments. Non‐adherent tumor cells were treated with Y27632 under 0 or 30 dyne/cm^2^ FSS for various durations. Cell survival was analyzed by PI apoptosis assay. n = 3 biological repeats for each condition. Representative images (g) and quantification (h) of the percentage of PI^+^ cells after shear and Y27632 treatment in CSCs. CSCs were treated with Y27632 under 30 dyne/cm^2^ FSS for 4 h. n = 3 biological repeats for each condition. Scale bar, 100 µm. Data were presented as mean ± SEM. Data acquired from the circulatory system were highlighted with blue dash line. The one‐way ANOVA followed by Tukey's post‐hoc test was adopted for comparison in (b–f) and (h). ns, no significant difference; *, p < 0.05; **, p < 0.01, and ***, p < 0.001.

Our results have demonstrated that non‐adherent tumor cells mechanically adapt to high FSS by reducing force transmission and mechanoresponses through cytoplasmic myosin‐mediated disruption of the binding of myosin with actin, which could serve as a self‐protection mechanism for the survival of CTCs. Therefore, it might be possible that targeting this survival signaling could re‐sensitize CTCs to shear‐induced death. To test this idea, both pharmacologic and genetic approaches were adopted to inhibit cytoplasmic p‐MLC. The treatment with 4‐HAP or Y27632 and silencing *MYH9* or *MYH10* reduced cytoplasmic p‐MLC and elevated FTE to the similar or even higher level of control cells under high FSS (Figures S6 and S7), thereby increasing DNA damage (Figure 6a–d and Figure S28a–d). This finding was also observed when non‐adherent tumor cells were circulated under high FSS (Figure 6e). Further, the inhibition of cytoplasmic p‐MLC significantly enhanced cell apoptosis under high shear stress (Figure 6f and Figure S28e,f). All the pharmacologic and genetic treatments had no obvious effect on DNA damage and apoptosis without FSS. These results suggest that cytoplasmic myosin mediates mechanoadaptation of non‐adherent tumor cells, which protects them from high shear‐induced DNA damage and apoptosis.

Cancer stem cells (CSCs) have been proposed to play essential roles in driving tumor metastasis, indicating that these malignant cells must hold survival advantage under all rate‐limiting factors, including blood shear stress during hematogenous dissemination. Indeed, our previous research has shown that CSCs are more resistant to shear‐induced apoptosis than non‐CSCs [23, 48], while the underlying mechanism remains poorly understood. Notably, CSCs showed higher level of activated myosin in the whole cell and cytoplasm than non‐CSCs in both presence and absence of FSS (Figures S29 and S30a–c). The enhanced cytoplastic p‐MLC could mediate lower FTE in CSCs, which was elevated to the similar level of non‐CSCs by the inhibition of cytoplasmic myosin (Figure S30d). Importantly, the increase of FTE in CSCs through Y27632 led to much higher DNA damage and death under high shear stress (Figure 6g,h and Figure S30e), indicating that CSCs acquire the survival advantage at least partially through cytoplasmic myosin‐mediated attenuation of FTE. We further investigated whether this mechanoadaptation could be a generalized survival mechanism for other non‐adherent cells under shear stress. Consistent with previous studies [7], Jurkat, leukemic human T lymphocyte, exhibited much higher resistance to shear‐induced destruction than breast CTCs (Figure 6f and Figure S31h). Remarkably, Jurkat cells showed much higher myosin activity in cytoplasm but not cortex (Figure S31a–c) and considerably lower FTE compared with breast cancer cells (Figure S31f). The inhibition of cytoplasmic myosin with Y27632 elevated the FTE of Jurkat cells to the similar level of MCF‐7 (Figure S31d–f). This increase of FTE sensitized Jurkat cells to shear‐induced destruction and notably enhanced cell death under FSS (Figure S31g,h), suggesting that cytoplasmic myosin‐mediated reduction of force transmission underpins T lymphocyte's survival under blood shear stress. In addition, human pancreatic cancer cells BxPC3 and human lung cancer cells A549 also exhibited as similar mechanoresponses, myosin subcellular localization and FTE as breast cancer cells under different levels of shear stress (Figures S32 and S33). These findings suggest that the observed accumulation of cytoplasmic p‐MLC and the reduction of FTE under high FSS are not cell type dependent but instead applicable to multiple cancer types, such as breast, pancreatic and lung cancer, as well as leukemia.

**FIGURE 7:**
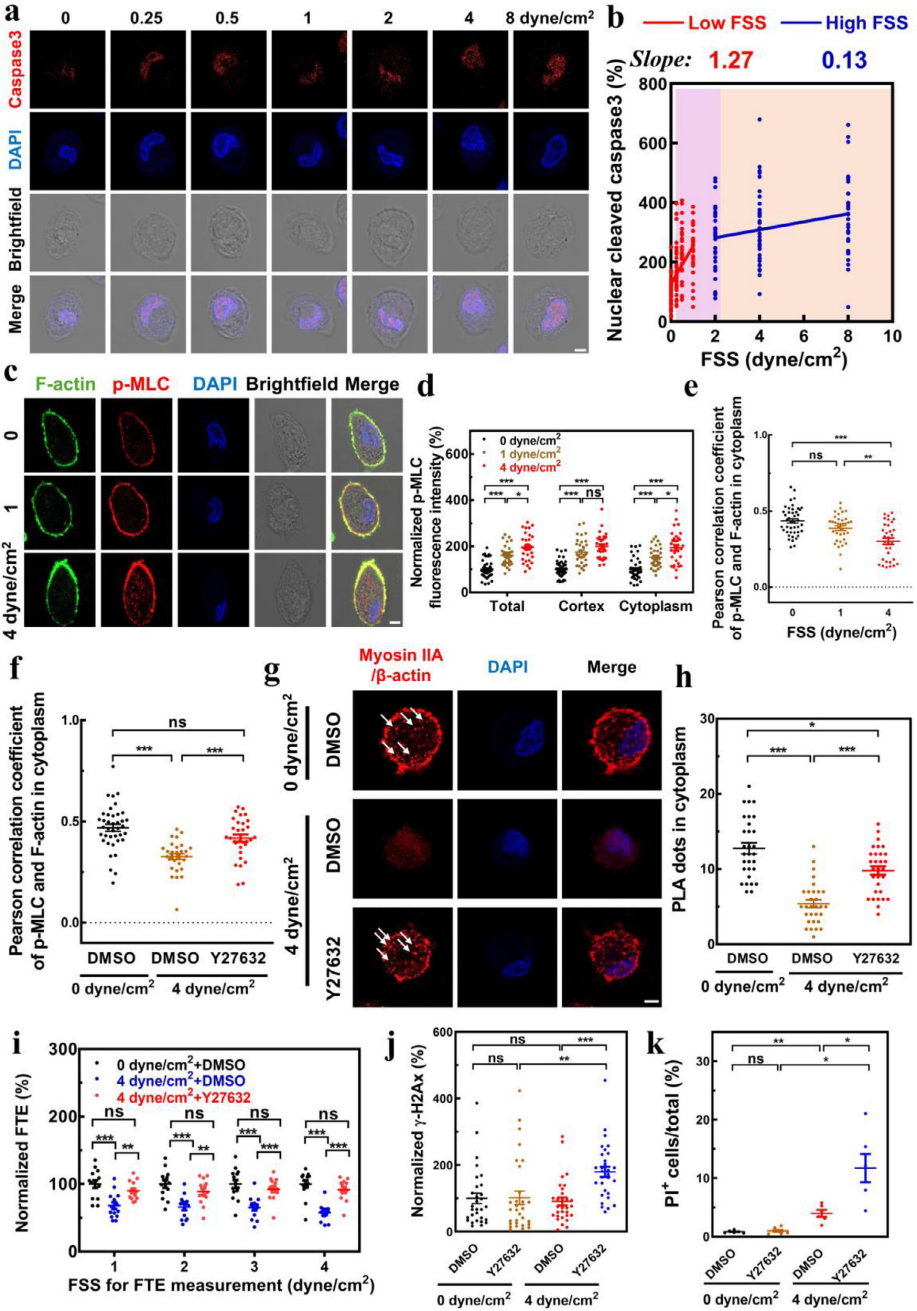
Patient‐derived primary tumor cells exhibit similar cytoplasmic myosin‐mediated mechanoadaptation under high FSS. Representative immunofluorescence images (a) and quantification (b) of nuclear cleaved caspase 3 after the treatment under 0, 0.25, 0.5, 1, 2, 4, and 8 dyne/cm^2^ FSS for an hour. Primary tumor cells were attached to PLL‐coated microfluidic chips and treated under varying levels of FSS for 1 h. The red and blue lines represented the linear regression of data within the ranges of low and high FSS. n = 30, 33, 30, 33, 32, 36, and 30 cells from 3 biological repeats for 0, 0.25, 0.5, 1, 2, 4, and 8 dyne/cm^2^, respectively. Scale bar, 5 µm. Representative immunofluorescence images (c), the quantification of p‐MLC in cytoplasm and cortex (d), and the co‐localization of p‐MLC with F‐actin (e) in primary tumor cells after the treatment under 0, 1, and 4 dyne/cm^2^ FSS for an hour. Primary tumor cells were treated similarly as in (a). The co‐localization of p‐MLC with F‐actin in cytoplasm was analyzed by calculating the Pearson correlation coefficient. n = 43, 35, and 32 cells from 3 biological repeats for 0, 1, and 4 dyne/cm^2^ FSS, respectively. Scale bar, 5 µm. (f) Co‐localization of p‐MLC with F‐actin after shear and Y27632 treatment. Primary tumor cells were attached to a PLL‐coated microfluidic chip and treated under 0 and 4 dyne/cm^2^ FSS for an hour and then with Y27632 for 10 min. The co‐localization of p‐MLC with F‐actin in cytoplasm was analyzed by calculating the Pearson correlation coefficient. n = 39, 31, and 31 cells from 3 biological repeats for 0 dyne/cm^2^ with DMSO, 4 dyne/cm^2^ with DMSO and 4 dyne/cm^2^ with Y27632, respectively. Representative PLA images of myosin IIA and β‐actin (g) and the quantification (h) of PLA dots in cytoplasm after shear and Y27632 treatment. Primary tumor cells were treated similarly as in (f). The interaction between myosin IIA and β‐actin was detected using PLA assay. White arrows indicated typical PLA dots. n = 30 cells from 3 biological repeats for each condition. Scale bar, 5 µm. (i) Normalized FTE of primary tumor cells after the shear and Y27632 treatment. Primary tumor cells were treated similarly as in (f). n = 14 cells from 3 biological repeats for each condition. (j) Normalized γ‐H2Ax of primary tumor cells after the shear and Y27632 treatment. Primary tumor cells were treated by DMSO or Y27632 during FSS. n = 30 cells from 3 biological repeats for each condition. (k) Percentage of PI^+^ primary tumor cells after shear and Y27632 treatment. Primary tumor cells were treated similarly as in (j). n = 6 biological repeats for each condition. Data were presented as mean ± SEM. The one‐way ANOVA followed by Tukey's post‐hoc test was adopted for comparison in (d), (h), and (k). Kruskal–Wallis one‐way ANOVA followed by Mann–Whitney U‐test with Bonferroni correction was adopted for comparison in (e,f) and (i,j). ns, no significant difference; *, p < 0.05; **, p < 0.01, and ***, p < 0.001.

To test whether the findings of mechanoadaptation and the underlying mechanisms could be extended to clinical samples, we retrieved primary tumor cells from breast cancer patients for shear treatment. Primary breast tumor cells exhibited much more rapid increase of nuclear cleaved caspase 3 under low FSS compared with high FSS (Figure 7a,b; the slope of the fitted line: 1.27 vs 0.13 AU per dyne/cm^2^) and reduced force transmission after high shear pre‐treatment (Figure 7i), suggesting the reduced mechanoresponses under high FSS. Furthermore, high FSS induced cytoplasmic myosin activation and decreased the colocalization and physical interaction between myosin and actin (Figure 7c–h). The inhibition of cytoplasmic, but not cortical myosin rescued myosin‐actin binding and low FTE in primary breast tumor cells to the similar levels of control cells under high FSS (Figure 7f–i and Figure S34). Importantly, pharmacologic inhibition of cytoplasmic myosin sensitized primary breast cancer cells to shear‐induced DNA damage and enhanced cell apoptosis under high FSS (Figure 7j,k). All these results demonstrate that not only cancer cell lines but also primary tumor cells harness this mechanoadaptation mechanism to evade shear‐induced destruction through the reduction of force transmission via cytoplasmic myosin‐mediated disruption of myosin with actin.

Taken together, these findings suggest that cytoplasmic myosin‐mediated mechanoadaptation may be a generalized self‐protection and survival mechanism of multiple non‐adherent cells under high FSS.

### 2.6 Transiently Targeting Cytoplasmic Myosin‐Mediated Mechanoadaptation During Hematogenous Dissemination Enhances CTC Apoptosis and Suppresses Tumor Metastasis

Our findings have demonstrated that high shear‐induced cytoplasmic p‐MLC reduces force transmission and mechanoresponses of non‐adherent tumor cells. This prompted us to investigate whether it was possible to eliminate more CTCs in blood circulation and thus suppress tumor metastasis by targeting this mechanoadaptive survival mechanism. To test this possibility, we adopted pNLuc plasmid in the Matador assay to measure the apoptosis of CTCs during hematogenous dissemination [49], in which the luciferase was only released from dying and dead cells but retained in alive cells. The number of dead cells that stably expressed pNLuc (231‐pNLuc) was linearly associated with the bioluminescence signal intensity (Figure S35a,b). Therefore, the bioluminescence intensity of peripheral blood could be utilized to represent the level of CTC death. 231‐pNLuc cells were pretreated with Y27632 or DMSO for an hour and inoculated into mice through tail vein injection, 12 h after which the whole blood was collected for bioluminescence imaging. The results showed that Y27632 enhanced the death of tumor cells during in vivo blood circulation (Figure S35c,d), which was not due to the cytotoxicity of the pharmacologic treatment (Figure S8l). These results suggest that targeting cytoplasmic myosin‐mediated mechanoadaptation effectively eliminates more CTCs.

**FIGURE 8:**
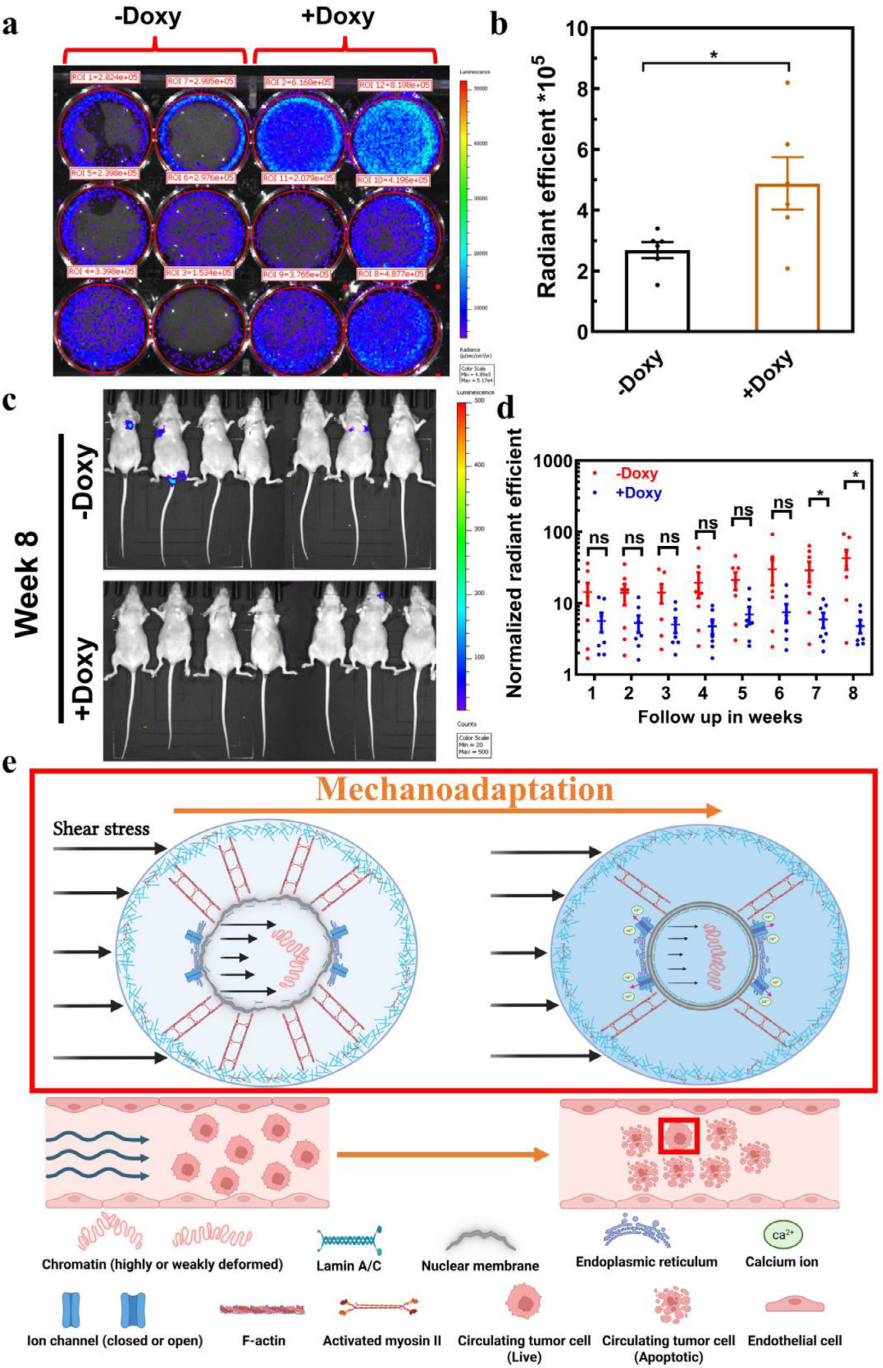
Transiently targeting cytoplasmic myosin‐mediated mechanoadaptation during hematogenous dissemination enhances CTC apoptosis and suppresses tumor metastasis. Bioluminescence images (a) and quantification of radiant efficient (b) of whole blood retrieved from mice. Doxy‐shROCK2 cells were treated with DMSO (‐Doxy) or doxycycline (+Doxy) for 2 days before the inoculation into mice through tail vein injection. After 12 h, the whole blood of mice was collected for bioluminescence imaging after the reaction with coelenterazine. n = 6 mice for each condition. Bioluminescence images (c) and quantification (d) of normalized radiant efficient of mice. Doxy‐shROCK2 cells were treated with DMSO or Doxy for 2 days before tail vein injection. The mice were not administrated with Doxy. Luminescence imaging in (c) was conducted using the exposure time of 3 min, and the radiant efficient at the indicated time points was quantified in (d) based on the signals of the whole mice. For each mouse, the radiant efficient was normalized to week 0. n = 7 mice for both ‐Doxy and +Doxy. Mann–Whitney U‐test was adopted for comparison in (b) and (d). ns, no significant difference; *, p < 0.05. (e) Schematic of cytoplasmic myosin‐mediated mechanoadaptation of non‐adherent tumor cells under high FSS. Under high shear stress, Lamin A/C‐mediated nuclear mechanosensing increases nuclear envelop tension and triggers calcium release from ER. The calcium signal further activates cytoplasmic myosin through ROCK, which disrupts its binding with actin to reduce the transmission of force from cell surface to nucleus. This cytoplasmic myosin‐mediated mechanoadaptation serves as a new survival mechanism of CTCs to protect them from shear‐induced DNA damage and death during hematogenous dissemination.

Since CTCs are the ‘seeds’ of distant metastases, efficient eradication of CTCs has the potential to suppress tumor metastasis. Myosin activity has multifaceted effects on various cellular functions, such as cell proliferation, migration, and invasion. It is thus important to temporally target cytoplasmic myosin only in blood circulation but avoid the potential ‘off‐target effects’ of myosin modulation after the exit of such milieu and re‐attachment to the metastasized organs. Toward this goal, MDA‐MB‐231 cells were transfected with doxycycline‐inducible ROCK2 shRNA to develop a stable cell line (Doxy‐shROCK2). After the induction with doxycycline, phosphorylated ROCK, cytoplasmic p‐MLC, cell migration and invasion were substantially decreased (Figures S36a–e and S38), which were gradually restored upon the removal of doxycycline (Figures S37a–f and S38). Doxycycline itself had no detectable effect on myosin activation or FTE (Figure S37g–i). Consistently, the reduction of cytoplasmic myosin significantly increased FTE (Figure S36f). These Doxy‐shROCK2 cells were pre‐treated with (+Doxy) or without (‐Doxy) doxycycline before inoculation into the tail vein of mice, which were not administrated with extra doxycycline. Doxycycline‐induced transient ROCK inhibition decreased cytoplasmic myosin activity of CTCs during blood circulation, which elevated force transmission and mechanoresponses to FSS (Figure S36), re‐sensitized CTCs to shear‐induced death (Figure 8a,b), and suppressed tumor metastasis in long term (Figure 8c,d). These findings indicate that cytoplasmic myosin‐mediated mechanoadaptation may provide a promising target for the development of new mechanotherapeutics to efficiently eradicate CTCs during hematogenous dissemination and eventually prevent tumor metastasis (Figure 8e and Figure S39a).

## 3 Discussion

One major route for tumor cells to metastasize to distant organs is through hematogenous dissemination, during which CTCs experience a variety of stressing factors, such as anchorage‐dependent cell death or anoikis, immune surveillance, cytokines as well as shear‐induced death [4]. As such, the vast majority of CTCs undergo stress‐induced death, which considerably limits the success rate of metastatic cascade. Therefore, hematogenous dissemination in vasculature could be one of the most vulnerable steps during the whole metastatic process, rendering it as a promising target for the prevention of metastasis. This study reports that in response to increasing FSS experienced in vasculature, non‐adherent CTCs redistribute activated myosin into cytoplasm, providing a ‘cytoskeletal shield’ by disrupting the binding of actin with myosin, which eventually reduces the transmission of the exerted force into chromatin and protects nucleus from excessive mechanical distortion. This serves as a self‐protection mechanism for CTCs to adapt to this harsh shearing microenvironment and evade shear‐induced death, a process known as mechanoadaptation, which addresses how CTCs survive the mechanical interrogation during hematogenous dissemination. Importantly, this mechanoadaptation response and the underlying mechanisms can be generalized to clinical samples and other non‐adherent cells, implicating the potential involvement of these unappreciated mechanisms in the survival of CTCs in the vasculature of patients with cancer.

In contrast to the poor understanding of non‐adherent cells, the mechanoadaptive phenomenon has been reported in adherent cells. For example, under fluid flow, endothelial cells realign cytoskeleton to minimize the total force transmitted to nucleus [50, 51]. Failed mechanoadaptation to FSS results in junctional damage and atherosclerotic lesion [52]. Furthermore, low cyclic stretch triggers prenuclear actin formation and nuclear softening mediated by H3K9 demethylation, both of which protect nucleus from stretch‐induced accumulation of DNA damage [53]. Intriguingly, actin is redistributed from the leading edge to cell centroid via DOCK8 in activated T cells during confined migration. This actin rearrangement further protects nucleus from confinement‐mediated deformation and DNA damage, suggesting that cytoskeletal protein relocalization may serve as a mechanoadaptive response under confinement [54]. The mechanoadaptive mechanism of non‐adherent CTCs discovered in this study, together with nuclear decoupling under cyclic strain of adherent cells [55], collectively suggest that living cells, irrespective of their adherent state, can leverage a “self‐defensive” strategy to protect their nuclei, which contain the essential genome, from force‐induced DNA damage. Our findings may inspire the development of new therapeutic modalities by targeting this mechanoadaptation to eliminate CTCs during hematogenous dissemination [56].

Non‐muscle myosin II activation is essential in force generation and mechanotransduction of epithelial cells [57], which play indispensable roles in various cellular functions, such as migration, invasion and division [58]. Intriguingly, recent work shows quick redistribution of myosin from cytoplasm to cortex that facilitates cell migration under spatial confinement, indicating distinct roles of cytoplasmic and cortical myosin [20]. Indeed, our study shows that cytoplasmic, but not cortical myosin activation, mediates mechanoadaptation of non‐adherent tumor cells and reduces the mechanoresponses to high FSS, which enables the survival of a subpopulation of CTCs during hematogenous metastasis. Thus, myosin in different subcellular regions may have distinct roles in cellular functions. Nevertheless, the dynamics of force‐induced myosin redistribution into different regions of a cell and the essential underpinning mechanisms remain unclear. The effects of subcellular myosin redistribution on various cellular behaviors need to be further investigated under varying levels of mechanical stimulations in the future [59, 60].

Myosin activation enhances its ATPase activity and facilitates force transmission and mechanotransduction in adherent cells [61, 62]. In contrast, our results suggest that in non‐adherent tumor cells, cytoplasmic myosin activation disrupts its binding with actin that further leads to reduced force transmission, which can be explained by shear‐induced cytoskeletal fluidization using the DPD model. Nevertheless, the detailed molecular mechanisms underlying force‐induced dissociation between myosin and actin warrant further investigation. The interaction between myosin and actin depends on myosin ATPase cycle [63], intracellular calcium level [64] and tropomyosin [65]. Considering the increased myosin activity and calcium signaling we found in non‐adherent tumor cells under shear stress, tropomyosin may potentially underlie high shear‐induced disruption of the binding of myosin with actin. Our results suggest that under high shear stress, tropomyosin exhibits reduced phosphorylation, suggesting the increase of tropomyosin activity (Figure S39b,c). Y27632 treatment rescues high shear‐induced tropomyosin dephosphorylation to some extent (Figure S39d,e), which may partially explain the restoration of the actin‐myosin binding upon Y27632 treatment (Figure S14c,d). These results indicate the potential roles of tropomyosin in myosin‐actin uncoupling under high shear stress. Further studies are required to elucidate how tropomyosin and/or ATPase regulate actin‐myosin binding and force transmission in non‐adherent cells under FSS. In line with our findings, recent studies show that myosin II activation enhances cytoskeletal tension and stiffens adherent cells but softens cells in non‐adherent state [66]. These seemingly contradictory results collectively implicate distinct contributions of myosin II activity to cell mechanics and mechanotransduction in non‐adherent and adherent cells. Thus, the roles of myosin II in cellular functions should be carefully interrogated in the context of adherent status.

It is documented that chromatin and Lamin A/C govern cellular mechanical responses to small (<3 µm) and large (3–10 µm) deformation [67, 68], respectively, indicating their distinct roles in mechanotransduction under different levels of mechanical stimuli. In this study, H2B displacement under both small and large FSS is less than 0.6 µm, which falls in the regime of small nuclear distortion. It thus likely reasons that chromatin may dominate how the nucleus senses and responds to the exerted FSS. Intuitively, the force within chromatin reflects the mechanical homeostasis inside the nucleus. The comparison of such force after the treatment of varying FSS indicates the efficiency of the force transmission process. However, the tools for direct measurement of mechanical force within chromatin are still lacking [69]. Alternatively, chromatin displacement and FTE have been adopted as a surrogate in this study to reflect the force within chromatin and force transmission from cell membrane into chromatin when the global chromatin mechanics remain unchanged. Future development of force biosensors within chromatin can empower the direct evaluation of force transmission. Nevertheless, the transmission of force from cell membrane into chromatin requires not only cytoskeletal elements and LINC complex, but also chromatin and the proteins linking chromosomes to nuclear lamina [70]. The linkage between nuclear lamina and chromatin is believed to play critical roles in force transmission from nuclear envelope to chromosome [71], which may further remodel the chromatin network to affect the accessibility and gene transcription [62, 72]. The roles of these associated proteins as well as nucleoskeleton (e.g., nuclear actin and myosin) in force transmission and mechanoadaptation of CTCs under varying levels of FSS warrant further investigation.

## 4 Conclusion

This study unveils a previously unappreciated mechanoadaptive survival mechanism of non‐adherent CTCs under high shear stress in both breast cancer cell lines and patient‐derived primary tumor cells. This is elicited through Lamin A/C‐mediated nuclear mechanotransduction to redistribute activated myosin into cytoplasm, which disrupts the binding of myosin with actin through force‐induced cytoskeletal fluidization, eventually attenuating force transmission into nucleus to protect CTCs from shear‐induced death. Transient inhibition of cytoplasmic myosin in vasculature diminishes such mechanoadaptation and restores CTC's mechanosensitivity to high FSS, which leads to reduced metastatic burden. In summary, this study uncovers a self‐protection mechanism of CTCs, which enables them to survive the harsh shearing milieu during hematogenous metastasis. These findings potentiate the development of new mechanotherapeutic strategies against metastasis by targeting this mechanoadaptation signaling.

## 5 Materials and Methods

### 5.1 Cell Maintenance

MCF‐7 (RRID: CVCL_0031) and MDA‐MB‐231 (RRID: CVCL_0062) breast cancer cell lines, BxPC3 (RRID: CVCL_0186) human pancreatic cancer cells and A549 (RRID: CVCL_0023) human lung cancer cells were purchased from ATCC. Jurkat E6.1 cells (RRID: CVCL_0367) were purchased from Procell company. MCF‐7 and Jurkat E6.1 were cultured with Gibco Roswell Park Memorial Institute (RPMI) 1640 Medium. MDA‐MB‐231 cells, BxPC3 cells and A549 cells were cultured with Dulbecco's Modified Eagle Medium (DMEM, HyClone). All mediums were supplemented with 10% FBS (HyClone) and 1% Penicillin/Streptomycin (HyClone). Patient‐derived primary breast tumor cells were purchased from Pricella Biotechnology Co., Ltd (Cat. No CP‐H146) and maintained in specialized human primary breast tumor cells culture medium (Pricella Biotechnology Co., Ltd, Cat. No CM‐H146) for no more than five passages. Primary breast tumor cells were authenticated by vimentin staining. Cells were cultured under 37°C and 5% CO_2_, and passaged every 2–3 days depending on the confluency. All the cells were verified by STR and tested negative for mycoplasma contamination periodically.

### 5.2 Treatment of Non‐Adherent Cells Under Fluid Shear Stress

Tumor cells were circulated under FSS as previously described [23]. Briefly, a peristaltic pump (Harvard) was coupled by a silicone micro‐tubing with the diameter of 0.51 mm, which was pre‐treated with BSA before experiments to prevent the potential cell attachment. 2 mL of cell solution (2 × 10^5^ cells/mL) was loaded to a syringe, which was used as the cell solution reservoir. During experiments, non‐adherent cells were circulated within the tube under 37°C and 5% CO_2_ for the indicated duration. The FSS on tube wall τ (dyne/cm^2^) was calculated using the equation τ = 4µQ/(πR^3^), where µ was the viscosity of the medium, Q was the flow rate and R was the radius of the tubing.

### 5.3 Treatment of PLL‐Adhered Cells Under FSS

1 × 10^5^ cells/mL of non‐adherent cells were attached to Poly‐L‐Lysine or PLL (Sigma‐Aldrich) coated microfluidic chips (Ibidi µ‐Slide I^0.2^ Luer, Cat. No. 80166) for 15 min. The non‐adhered tumor cells were washed away using 0.2 dyne/cm^2^ shear flow for 10 s. Further, varying wall FSS was applied by changing the flow rate through peristaltic pump and micro‐tubing (Figure S1a). The wall FSS on microfluidic chips was calculated strictly following the manufacturer's instructions as τ [𝑑𝑦𝑛e/𝑐𝑚^2^] = 𝜂 [𝑑𝑦𝑛e·s/𝑐𝑚^2^]·512.9·Φ [𝑚𝑖𝑛/𝑚L], where 𝜂 was viscosity of medium, and Φ was flow rate.

### 5.4 Pharmacologic Treatment

Cells were treated by 50 µM 4‐Hydroxyacetopophenone (4‐HAP, Sigma‐Aldrich), 20 µM Blebbistatin (Bleb, Sigma‐Aldrich), 20 µM Y‐27632 (Y27, Sigma‐Aldrich), 20 µM ML‐7 (Sigma‐Aldrich), 20 nM calyculin A (MCE, Cat. No HY‐18983/CS‐54), 10 µM si*LMNA* (Thermo Fisher Scientific, Cat. No 4390824), 50 nM Thapsigargin (Tg, Thermo Fisher Scientific, Cat. No 2403704), 2 µM Yoda1 (MCE, Cat. No HY‐18723), 10 µM BAS00602705 (MCE, CAS No. : 317321‐89‐4) or 0.5 µM Doxorubicin (MCE, Cat. No HY‐15142) for the indicated durations before or after the shear treatment. 1 mM ethylene glycol‐bis (β‐aminoethylether)‐N,N,N′,N′‐tetraacetic acid (EGTA, Sigma‐Aldrich, Cat. No E3889) was added to chelate calcium ions in cell culture medium for an hour. For 4‐HAP treatment, ethanol was used as solvent for 4‐HAP, the concentration was quite low (about 0.01% v/v) and did not induce DNA damage.

### 5.5 Plasmid Transfection

Cells were transfected with plasmids using Lipofectamine 3000 (Life Technologies, Carlsbad, CA, USA) following the manufacturer's protocol. The treated cells were used for experiments 3 days after transfection. For the transfection of siRNA and overexpression plasmid of Lamin A/C, MCF‐7 cells were cultured on tissue culture plates (Thermo Fisher Scientific, Cat. No 140675) for 24 h. Cells were transfected with Lamin A/C siRNA or the overexpression plasmids when the confluence reached 60%. For each well, 3.75 µL Lipo3000 was diluted in 125 µL OMEM (Thermo Fisher Scientific, Cat. No 31985062). 0.05 nmol siRNA targeting *LMNA* or 2 µg mRuby2‐LaminA‐C‐18 plasmid was gently mixed with 5 µL P3000 and diluted in 125 µL OMEM. The Lipo3000 solution and the siRNA or plasmid solution were then gently mixed, followed by the incubation at room temperature for 20 min. Finally, the mixture was added to cells and incubated in the incubator under 37°C and 5% CO_2_. The efficiency of gene knockdown and overexpression was quantified by immunoblotting analysis at 72 h after transfection. The siRNA sequences used in this study were provided in Supplementary Table I. The plasmids used in this study were listed below: Gata3‐T2A‐H2B‐eGFP donor was a gift from Janet Rossant (plasmid # 113119); PECAM‐H2B‐GFP was a gift from Victoria Bautch (plasmid # 14689); pcdna nesprin TS was a gift from Daniel Conway (plasmid # 68127); pAAV.CAG.GCaMP6f.WPRE.SV40 was a gift from Douglas Kim & GENIE Project (plasmid # 100836); mRuby2‐LaminA‐C‐18 was a gift from Michael Davidson (plasmid # 55901); pinducer 20 DN‐KASHΔPPPL was a gift from Daniel Conway (plasmid # 129280); pinducer 20 DN‐KASH was a gift from Daniel Conway (plasmid # 125554); pNLuc was a gift from Koen Venken (plasmid # 118058). pMXs‐IP‐ SCAT3.2 caspase3 sensitive FRET sensor was a gift from David Scadden (plasmid # 172497). These plasmids were all purchased from Addgene. si‐*MLCK*, doxycycline inducible shROCK2 and EGFP‐TERF1 were designed and purchased from Yanming Biotechnology Co., Ltd.

### 5.6 Immunofluorescence Staining

Cells were fixed using 4% paraformaldehyde (Thermo Fisher Scientific) for 30 min and further washed by PBS twice. 0.5% Triton X‐100 (SAFC) in 1% BSA solution (VWR Life Science) was used to permeabilize the cells for 1 h. After washing twice by PBS, the permeabilized cells were further incubated with the first antibody correspondingly at 4°C overnight. Then, the cells were washed by PBS and stained with the secondary antibody for 1 h in dark. Finally, after washing twice, the nuclei were counterstained with Hoechst (Thermo Fisher Scientific) and imaged by Leica SPE confocal microscopy with 63 × 1.4 NA oil‐immersion objective. The wavelength of the excitation light for DAPI, green fluorescence and red fluorescence of Leica SPE confocal microscopy was 405 nm, 488 nm and 568 nm, respectively.

First antibodies used in this study were: Phospho‐Myosin Light Chain 2 Ser19 antibody (Cell signaling, Cat. No. 3671), Vinculin antibody (abcam, Cat. No 129002), Phospho‐Histone H2A.X Ser139 (γ‐H2AX) antibody (Cell signaling, Cat. No. 9718), Lamin A and Lamin C (Lamin A/C) antibody (abcam, Cat. No 238303), MLCK antibody (abcam, Cat. No 76092), Phospho‐ROCK ser 1366 antibody (Thermo Fisher Scientific, Cat. No PA5‐34895), Phospho‐Lamin A/C Ser22 antibody (Thermo Fisher Scientific, Cat. No PA5‐17113), CD24 antibody (Proteintech, Cat. No 67627‐1), CD44 antibody (abcam, Cat. No ab81424), Sox2 antibody (Proteintech, Cat. No 66411‐1), Oct4 antibody (abcam, Cat. No 19857), Nanog antibody (abcam, Cat. No 109250), E‐cadherin antibody (Cell signaling, Cat. No. 3195), BAF antibody (abcam, Cat. No 129184), HP1 antibody (abcam, Cat. No 109028), Phospho‐tropomyosin Ser283 (Thermo Fisher Scientific, Cat. No 600‐401‐J52) and Cleaved Caspase‐3 Asp 175 antibody (Cell signaling, Cat. No. 9661).

Secondary antibodies used in this study were: Alexa Fluor 488 (Cat. No. ab150077), Alexa Fluor 647 (Cat. No. ab150079) and Alexa Fluor 594 (Cat. No. ab150116).

### 5.7 Quantitative PCR

The mRNAs of cells were extracted using Aurum Total RNA Mini Kit (Bio‐Rad). The sequences of all the primers were listed in the Supplementary Table 1. The cDNAs were synthesized using cDNA Synthesis Kit (Thermo Fisher Scientific). Real‐time PCR was performed with CFX96 Real‐Time System (Bio‐Rad).

### 5.8 Calculation of Pearson Correlation Coefficient Between p‐MLC and F‐Actin

p‐MLC was first stained through immunofluorescence as previously described, followed by the staining of F‐actin using phalloidin (abcam, Cat. No 112125). Images were taken under Leica SPE confocal microscopy with 63 × 1.4 NA oil‐immersion objective. Before measurement, images were converted into 8 bit and the background of each image was subtracted by the Background Subtracted plugin of ImageJ. Further, the images of two channels were stacked and Pearson correlation coefficient was calculated by Coloc 2 in Fiji software.

### 5.9 Western Blotting

Proteins were extracted from cells by RIPA and protein extraction buffer (Thermo Fisher Scientific). After the measurement of protein concentration, proteins in each condition were aliquoted to ∼50 µg. The gel electrophoresis (BIO‐RAD) was used to separate proteins with different molecular weights. Proteins in 10% SDS‐PAGE gel were transferred to polyvinylidene difluoride (PVDF) western blotting membrane with pore size of 0.2/0.44 µm through Trans‐Blotting Turbo (BIO‐RAD). 3% BSA solution was used to block the unspecific binding for an hour at room temperature. The membrane was further incubated with desired primary antibody at 4°C overnight. Finally, after the staining with secondary antibody (abcam, Cat. No 6715) for an hour and washing twice by TBST (Thermo Fisher Scientific, J77500.K2), the membrane was imaged by Clarity MAX Western ECL Blotting Substrates and ChemiDoc MP Imaging System (BIO‐RAD).

### 5.10 Co‐Immunoprecipitation

Cells were solubilized in the lysis buffer containing 20 mM Tris–HCl, 100 mM KCl, 5 mM MgCl_2_, 0.5% Triton X‐100, 1 mM DTT and 1 mM PMSF (Beyotime) followed by centrifugation at 12 000 g for 20 min at 4°C. The supernatant was transferred to a conical centrifuge tube of 1.5 mL and incubated with 1.0 µg of the appropriate control IgG (abcam, Cat. No 6715) and 20 µL of resuspended volume of Protein A/G PLUS‐Agarose Beads (Santa Cruz) at 4°C for 30 min. Then, the tube was centrifuged at 1000 rpm for 5 min at 4°C and the supernatant was transferred to a conical centrifuge tube of 1.5 mL on ice, followed by adding 2 µg of primary antibody at 4°C overnight. Further, 20 µL of resuspended volume of Protein A/G PLUS‐Agarose Beads was added to and incubated with the supernatant at 4°C on a rocker platform for an hour, followed by washing Agarose Beads four times with lysis buffer. Finally, proteins were subjected to SDS–PAGE and the related protein expression was quantified through western blotting analysis. Antibodies used were listed as below: anti‐beta actin antibody (abcam, Cat. No 227387) and anti‐myosin IIA antibody (abcam, Cat. No 238131).

### 5.11 Calcein AM/PI Assay

Non‐adherent tumor cells after pharmacologic treatments were collected and stained by Calcein AM/PI Double Staining Kit (Sigma‐Aldrich, Cat. No 04511) for an hour. The percentages of tumor cells stained with Calcein AM/PI were analyzed by BD Accuri C6 Flow Cytometer.

### 5.12 Measurement of p‐MLC Cortex/Cytoplasm Ratio

The cortex thickness (∼450 nm) was measured by full width at half maximum of F‐actin as preciously established [30]. Further, we quantified total fluorescence intensity of p‐MLC as integrated fluorescence intensity in the cell cross section and the fluorescence intensity of cortical p‐MLC as integrated fluorescence intensity within 450 nm beneath cell boundary. Finally, the p‐MLC cortex/cytoplasm ratio was determined as below:

(1) $$\frac{p-\mathrm{ML}C_{\mathrm{cortex}}}{p-\mathrm{ML}C_{\mathrm{cytoplasm}}}=\frac{\Sigma_{\mathrm{cortex}}^{I}/A_{\mathrm{cortex}}}{\left(\Sigma_{\mathrm{cell}}^{I}-\Sigma_{\mathrm{cortex}}^{I}\right)\left(A_{\mathrm{cell}}-A_{\mathrm{cortex}}\right)}$$

Here, $\Sigma_{\mathrm{cell}}^{I}$ and $\Sigma_{\mathrm{cortex}}^{I}$ were integrated p‐MLC fluorescence intensity in cell cross section and 450 nm beneath cell boundary. *A_cell_* and *A_cortex_* were area of cell cross section and 450 nm beneath cell boundary. The quantification was conducted in ImageJ 1.5K.

### 5.13 Quantification of FRET Index

FRET index of pcdna nesprin tension sensor (TS) and caspase 3 FRET sensor were quantified by Leica TCS SP8 MP multiphoton microscope with 63 × 1.4 NA oil‐immersion objective. Briefly, cells were transfected with pcdna nesprin TS or caspase 3 FRET sensor 3 days before experiment. The cells that expressed relatively similar level of pcdna nesprin TS or caspase 3 FRET sensor were chosen as targets. The pcdna nesprin TS and caspase 3 sensitive FRET sensor were excited at 405 nm. The channels of donor (cyan) and receptor (yellow) were recorded. For the FRET measurement under FSS, non‐adherent cells were attached to PLL‐coated chips. Further, automatic focusing was utilized to avoid potential vibration during shear stress application. The FRET index was defined as fluorescence intensity ratio of receptor (yellow) and donor (cyan). Due to the expression of pcdna nesprin TS predominantly around nucleus, FRET index was quantified 1.5 µm around the nucleus. The FRET index of caspase 3 sensitive FRET sensor was quantified within cell boundary.

### 5.14 Living Cell Imaging of Calcium and Lamin A/C Under FSS

Cells were transfected with pAAV.CAG.GCaMP6f.WPRE.SV40 plasmid 3 days before experiment. The transfected cells were trypsinized and attached to PLL‐coated microfluidic chip. Images of green fluorescence (excited at 488 nm) were taken under Leica SPE confocal microscopy with 63 × 1.4 NA oil‐immersion objective every minute during FSS application. Automatic focusing was utilized to avoid potential out‐of‐focus effect.

For the dynamic tracking of Lamin A/C, cells were transfected with mRuby2‐LaminA‐C‐18 2 days before experiment. The transfected cells were trypsinized and adhered to PLL‐coated microfluidic chip. Images of red fluorescence (excited at 568 nm) were taken under Leica SPE confocal microscopy with 63 × 1.4 NA oil‐immersion objective every minute during the course of FSS application. Automatic focusing was utilized to avoid potential out‐of‐focus effect.

### 5.15 Quantification of Cross Section Area, Nuclear Area, Nuclear Volume and Excess of Perimeter of Nuclear Envelop

The cross section was defined as the section with maximum area under z‐scanning of Leica MM AF software. Nuclear area and nuclear volume were measured by Image 1.5K, while Hoechst staining was used to determine nuclear outlines. To quantify the excess of perimeter of nuclear envelop (EOP_NE_), Lamin A/C was stained to determine nuclear outlines. When measuring EOP_NE_, a free‐hand lane was first plotted to fit the outlines of nucleus and the perimeter of free‐hand lane was defined as P_m_. Further, an ellipse with minimal perimeter was used to enclose nucleus and the perimeter of ellipse was defined as P_e_. Finally, the EOP_NE_ was calculated as:

(2) $$EOP_{NE}=\frac{P_{m}-P_{e}}{P_{e}}$$

### 5.16 Quantification of H2B Displacement

The H2B displacement was quantified as previously described [62]. Briefly, cells were transfected with Gata3‐T2A‐H2B‐eGFP plasmid 3 days before experiments. These cells were trypsinized and attached to PLL‐coated microfluid chip for 15 min. During FSS application, fifty images of nucleus were taken within 46.296 s by Leica SPE confocal microscopy with 63 × 1.4 NA oil‐immersion objective. Further, H2B displacement map was measured from fifty images through online available MATLAB script [29]. Briefly, the fifty images were divided into five cycles. For each cycle, the H2B displacement in every pixel between each two images was calculated using the MATLAB script and the maximum H2B displacement within ten images would be exported. After averaging the maximum H2B displacement among five cycles, the final H2B displacement was exported and the H2B displacement map was plotted based on the value of each pixel.

### 5.17 Quantification of Force Transmission Efficiency

The H2B displacement in each pixel was exported to Excel and the average H2B displacement was used to calculate FTE as blow:

(3) $$\mathrm{Force}\;\mathrm{transmission}\;\mathrm{efficiency}=\frac{H2B\;\mathrm{displacement}\;\left(\mathrm{nm}\right)}{\mathrm{stress}\;\left(\mathrm{dyne}/cm^{2}\right)}$$

Here, H2B displacement was the average value of H2B displacement from H2B displacement map, and stress was the FSS applied to cells.

### 5.18 Quantification of Mean Square Displacement of TERF1

Cells were transfected with EGFP‐TERF1 2 days before experiments. These cells were then trypsinized and attached to PLL‐coated microfluid chip for 15 min and subjected to different levels of FSS for an hour. Further, 1500 images of EGFP‐TERF1 (excited at 488 nm) were taken within 30 min (50 frames/minute) by Leica SPE confocal microscopy with 63 × 1.4 NA oil‐immersion objective. Automatic focusing was utilized to avoid potential out‐of‐focus effect. The images were then converted to 8 bit and the noisy was removed by background subtraction in ImageJ software. Finally, a previously reported MATLAB program was used to detect and track the fluorescence particle through statistical algorithms and relevant motions during succeeding frames [62].

### 5.19 Fluorescence Lifetime Imaging Microscopy (FLIM) Measurement

Cells after FSS were collected and incubated with ER Flipper‐TR (Cytoskeleton, Cat. No CY‐SC021) diluted by cell culture medium in 1:1000 ratio for 15 min. Further, FLIM of ER Flipper‐TR was taken by Nikon A1/SIM/STORM super‐resolution/confocal microscope with Olympus Plan Apo 60×/1.42 oil objective using 12.5 µs dwell time. The fluorescence lifetime was quantified by ImageJ software.

### 5.20 Proximity Ligation Assay

Cells after FSS were collected, fixed and permeabilized by 0.5% Triton X‐100 (SAFC) in 1% BSA solution (VWR Life Science). Myosin‐actin interaction was analyzed by Duolink In Situ Red Starter Kit Mouse/Rabbit kit (Sigma‐Aldrich, Cat. No DUO92101) according to manufacturer's instructions. Briefly, cells were blocked by Duolink Blocking Solution (Sigma Aldrich) for 60 min in a humidity chamber at 37°C, and then incubated with anti‐beta actin antibody (abcam, Cat. No 227387) and anti‐myosin IIA antibody (abcam, Cat. No 238131) at 4°C overnight. Further, PLUS and MINUS PLA probes were incubated with the cells for an hour at 37°C. After ligation and amplification, cells were washed with washing buffers twice and cell nucleus was stained by Duolink In Situ Mounting Medium with DAPI (Sigma‐Aldrich, Cat. No DUO82040). Finally, PLA fluorescence was imaged under Leica TCS SP8 MP multiphoton microscope with 63 × 1.4 NA oil‐immersion objective. The number of PLA dots was quantified by ImageJ software.

### 5.21 Propidium Iodide (PI) Assay

Cells after treatment were collected and washed with PBS twice. Further, cells were incubated with 4 µM PI (Thermo Fisher Scientific, P1304MP) for 5 min. Finally, the images of PI fluorescence were taken under Leica SPE confocal microscopy with 20×objective. The fluorescence quantification was performed in Image 1.5K.

### 5.22 Cancer Stem Cell Selection in Fibrin Gels

CSCs were selected by fibrin gels as previously described [48]. Briefly, MCF‐7 cells (7 × 10^5^ cells/mL) were mixed with fibrinogen solution (1 mg/mL, Salmonics LLC) in T7 buffer. After 15 min mixture at room temperature, 150 µL of fibrinogen‐cell mixture was added to one well of a 24‐well plate that was pre‐seeded with 6 µL 0.1U/µL thrombin (Salmonics LLC). After incubation in a cell culture incubator for 30 min, 1 mL of the medium 1640 was added to each well. After 5 days, the fibrin gel was digested by fibrinase (Salmonics LLC) and fibrin‐selected cells were collected as CSCs for further study.

### 5.23 Transwell Migration and Invasion Assay

Transwell migration and invasion assay were conducted through transwell cell culture chambers (8 µm pore polycarbonate, Corning Costar, USA, Cat. No 3422). For the migration assay, 200 µL of cell suspension containing 5 × 10^4^ cells was placed into the upper chamber with DMEM, whereas the bottom chamber was filled with 600 µL DMEM containing 10% FBS. The cells were further incubated at 37°C and 5% CO_2_ for 24 h. After that, the cells were fixed by 4% paraformaldehyde for 15 min. The top chamber surface was cleaned using a cotton swab and the bottom chamber surface was stained using 0.1% crystal violet for 20 min. For the invasion assay, the upper chamber was coated with 60 µL, 2 mg/mL Matrigel (Corning Costar, USA, Cat. No 354230) and incubated at 37°C and 5% CO_2_ for 2 h before cell seeding. Further, 200 µL of cell suspension containing 1 × 10^5^ cells was placed onto the polymerized Matrigel in the upper chamber, whereas the bottom chamber was filled with 600 µL DMEM with 10% FBS. The cells were further incubated at 37°C and 5% CO_2_ for 24 h. After that, the cells were fixed by 4% paraformaldehyde for 15 min. The top chamber surface was cleaned using a cotton swab and the bottom chamber surface was stained using 0.1% crystal violet for 20 min. Images were taken by Nikon Ti2A microscopy with 20×objective.

### 5.24 Dissipative Particle Dynamics Simulation of Cellular Behavior Under FSS

DPD is a mesoscopic particle‐based coarse‐graining (CG) simulation method for soft matter [73, 74], which has been widely used to simulate systems such as lipid membranes, polymers, natural protein biomaterials, and blood cells [38, 39]. Here we adopted the DPD method to simulate the dynamics of non‐adherent tumor cells under FSS at the whole‐cell level via explicitly considering cellular components, including the cell cortex, cytoskeletal actomyosin, nuclear lamina, cytoplasm, and nucleoplasm.

We modelled cell cortex and nuclear lamina with spring‐based triangular networks, an approach that has been widely used in DPD models of blood cells [38, 39]. In the model, each edge of the triangle was a linear spring with the potential

(4) $$U_{B,c}=\frac{1}{2}K_{c}{\left(r-r_{0,c}\right)}^{2}$$

for cortex network, and

(5) $$U_{B,n}=\frac{1}{2}K_{n}{\left(r-r_{0,n}\right)}^{2}$$

for lamina network. *K_c_* and *K_n_* were spring constants; *r* was the edge length; and *r*_0,*c*_ and *r*_0,*n*_ were equilibrium edge lengths.

The actin filament was modelled as the bead‐and‐spring polymer chains. In our model, each polymer chain, consisting of 9 beads, represented an actin filament. We randomly generated 400 polymer chains in the cytoplasm, half of which were connected to the nuclear lamina with the other end free, and the other half were connected to the cortex with the other end free. The actin filaments were bipolar, connecting to the cell cortex or nucleus as the plus end and the other end as the minus end.

A harmonic bond potential was applied on all neighboring beads of the polymer chains to constrain the bond length to a preferred value:

(6) $$U_{B,p}=\frac{1}{2}K_{p}{\left(r-r_{0,p}\right)}^{2}$$

where *K_p_* was the spring constant, *r* and *r*_0,*p*_ were the stretched bond length and the equilibrium bond length, respectively. The bending resistance of polymer chains was applied by a harmonic angle potential on the adjacent three beads:

(7) $$U_{\theta }=\frac{1}{2}K_{\theta }{\left(\theta -\theta_{0}\right)}^{2}$$

where *K*_θ_ was the spring constant, θ and θ_0_ were the angle of the adjacent three beads and the equilibrium angle, respectively.

During the simulation, bonds were stochastically created and broken between the polymer chains, mimicking the kinetics of myosin II and actin filament, where these dynamic bonds implicitly modelled myosin II. Once the myosin bond was formed, the harmonic potential was applied:

(8) $$U_{B,m}=\frac{1}{2}K_{m}{\left(r-r_{0,m}\right)}^{2}$$

where *K_m_* was the spring constant, *r* and *r*_0,*m*_ were the stretched bond length and the equilibrium bond length, respectively. The kinetics of myosin II and actin filament were described in details in the next section.

In addition, the cytoplasm and nucleoplasm were modelled as free solvent beads. All of the mechanical parameters of cellular components were estimated from tumor cells and summarized in Supplementary Table 2.

Kinetics of actin‐ and myosin‐based cytoskeleton. The myosin swinging cross‐bridge model has been widely utilized to understand the mechanism of ATP‐driven movement of myosin along actin filament [75], where the activity of myosin as a molecular motor is powered by its head groups which bind and hydrolyze ATP to provide the energy. We used a simple version of the swinging cross‐bridge model to capture the movement of myosin along actin filament and the disassembly of the actomyosin with a minimal number of parameters. There were two bound states (original conformation and swinging‐forward conformation) of the myosin‐actin complex and one unbound state in our model. Similar to the other models [76, 77], we assumed that all of the transitions between these states were well modelled as a first‐order reaction, whose reaction rates were force‐dependent and consistent with Bell's approximation. In our model, the cross‐bridge cycle exhibited catch‐bond behavior:

(9) $$k_{\mathrm{fw}}=k_{\mathrm{fw}}^{0}\exp\left(-\frac{F}{F_{\mathrm{fw}}}\right)$$

where *k*_fw_ donated the rate at which the myosin bond dissociated from the currently bound actin bead and then bound to the next bead toward the plus end of the actin polymer (i.e., forward step, Figure 4 b), $k_{\mathrm{fw}}^{0}$ and *F*_fw_ were force‐free reaction rate and characteristic force value, respectively. In our model, myosin returned to its original conformation and thereby generated contraction force (i.e. power‐stroke) through the elastic potential energy *U*_*B*,*m*_ of myosin bond [76].

Conversely, considering the reversibility of the cross‐bridge cycle [75], the inverse cross‐bridge cycle exhibited a slip‐bond behavior:

(10) $$k_{\mathrm{bw}}=k_{\mathrm{bw}}^{0}\exp\left(\frac{F}{F_{\mathrm{bw}}}\right)$$

where *k*_bw_ donated the rate at which the myosin bond dissociated from the currently bound actin bead and then bond to the prior bead of actin polymer (i.e., backward step), $k_{\mathrm{bw}}^{0}$ and *F*_bw_ were force‐free reaction rate and characteristic force value, respectively. And *F* = *K*_m_(*r* − *r*_0,*m*_) was the force within the myosin bond.

Combination of Equations (9) and (10) modelled the catch‐slip behavior of the cross‐bridge cycle under mechanical force to simulate the tensional homeostasis of the actomyosin [78]. When the tension within actomyosin was small, *k*_fw_ dominated and myosin moved toward the plus end of actin filaments, increasing the tension; conversely, when the tension was large, *k*_bw_ dominated and myosin moved toward the minus end, decreasing the tension.

Considering that myosin also acted as the cross‐linker between actin filaments in addition to regulating tension via the forward/backward step movement [78], we introduced the binding and unbinding of myosin with actin filament, respectively,

(11) $$k_{\mathrm{on}}=k_{\mathrm{on}}^{0}$$

(12) $$k_{\mathrm{off}}=k_{\mathrm{off}}^{0}\exp\left(\frac{F}{F_{\mathrm{off}}}\right)$$

where $k_{\mathrm{on}}^{0}$ and $k_{\mathrm{off}}^{0}$ were force‐free reaction rates (Figure 4b). We assumed that *k*_on_ remained constant. *F*_off_ was characteristic unbinding force. Note that experiments showed that myosin detachment exhibited catch‐slip behavior [79]. Here Equation (12) ignored the catch‐bond behavior because the cross‐bridge cycle was triggered when the force was small in our model.

We chose reaction parameters in Equations (9) to (12) to maintain force homeostasis through actin sliding under external forces, mimicking cellular homeostasis. The reaction parameters were compatible with the experimental measurements and summarized in Supplementary Table 2.

Given the reaction rate, we obtained the probability of the reaction occurring during the time interval δ*t* as [80]

(13) $$p_{i}=1-\exp\left(-k_{i}\delta t\right)$$

where the subscript “i” was one of “on”, “off”, “fw” and “bw” indicating the reaction rates of myosin binding/unbinding, myosin forward/backward movement, respectively. During each time interval δ*t* of the simulation, the probability *p_i_* was compared to a uniform random number *p*_ran_ ∈ [0, 1], and the reaction occurred only if *p*_ran_ < *p_i_*.

DPD simulation method. In the DPD simulations, the motion of the beads was governed by the Newton's equations,

(14) $$\frac{dr_{i}}{\mathrm{dt}}=v_{i},\;m_{i}\frac{dv_{i}}{\mathrm{dt}}=F_{i}$$

The total force on bead *i* was given by

(15) $$F_{i}=\sum_{j\neq i}\left(F_{\mathrm{ij}}^{C}+F_{\mathrm{ij}}^{D}+F_{\mathrm{ij}}^{R}\right)+F_{i}^{A}$$

where $F_{\mathrm{ij}}^{C}$, $F_{\mathrm{ij}}^{D}$ and $F_{\mathrm{ij}}^{R}$ were the conservative force, the dissipative force and the random force, respectively. And $F_{i}^{A}=-\frac{\partial U_{A}}{\partial r_{i}}$ was the additional conservative force which was the negative gradient of a potential *U_A_*, such as the bond force and angle force in polymer system.

The standard forms of the pairwise forces $F_{\mathrm{ij}}^{C}$, $F_{\mathrm{ij}}^{D}$ and $F_{\mathrm{ij}}^{R}$ between bead i and bead j were represented by

(16) $$\begin{array}{c} F_{\mathrm{ij}}^{C}=a_{\mathrm{ij}}\omega \left(r_{\mathrm{ij}}\right)e_{\mathrm{ij}}\\ F_{\mathrm{ij}}^{D}=-\gamma \omega^{2}\left(r_{\mathrm{ij}}\right)\left(e_{\mathrm{ij}}\cdot v_{\mathrm{ij}}\right)e_{\mathrm{ij}}\\ F_{\mathrm{ij}}^{R}=\sigma \omega \left(r_{\mathrm{ij}}\right)\xi_{\mathrm{ij}}\Delta t^{-1/2}e_{\mathrm{ij}} \end{array}$$

with the normalized distribution function

(17) $$\omega \left(r_{ij}\right)=\left\{\begin{array}{c} 1-r_{ij}/r_{c},\;\;\;r_{ij}<r_{c}\\ 0,\;\;\;r_{ij}\geq r_{c} \end{array}\right.$$

In Equation (16), *a_ij_* > 0 was the potential parameter representing the maximum repulsion between beads i and j, and *a_ii_* = 25 was used with a bead density of 3 in general DPD systems [73]. Hence, *a_ij_* was larger than 25 for a bead‐bead repulsion interaction while it was smaller than 25 for an attraction between two beads. *r_ij_* = |r_*i*_ − r_*j*_| was the distance between beads i and j, e_*ij*_ = (r_*i*_ − r_*j*_)/*r_ij_* was the unit vector from bead j to i, and v_*ij*_ = v_*i*_ − v_*j*_ was the relative velocity between beads i and j. γ and σ were the parameters related to each other as σ^2^ = 2γ*k_B_T*, where *k_B_T* was the Boltzmann constant and *T* was the system temperature. The standard values σ = 3 and γ = 4.5 were used as the previous study [73]. ξ_*ij*_ was a Gaussian random number with zero mean and unit variance. Δ*t* was the timestep size. *r_c_* was an interaction cutoff distance. The values of *a_ij_* used in the current study were summarized in Supplementary Table 3.

Parameter values. For the DPD approach, one usually makes use of reduced units for the physical quantities [73]; hence, it is of great importance to map the DPD model units into physical units. Following the mapping strategy widely used in the DPD simulations of red blood cells [39], we obtained an estimate of the physical length, force and time scales as follows:

(18) $$\left[L\right]=\frac{D_{c,\mathrm{EXP}}}{D_{c,\mathrm{DPD}}},\;\;\;\left[F\right]=\left[L\right]\frac{E_{c,\mathrm{EXP}}}{E_{c,\mathrm{DPD}}},\;\;\;\left[t\right]=\left[L\right]\frac{E_{c,\mathrm{DPD}}}{E_{c,\mathrm{EXP}}}\frac{\eta_{\mathrm{EXP}}}{\eta_{\mathrm{DPD}}}$$

where the subscripts “DPD” and “EXP” represented the values from DPD simulation and experiments, respectively. *D_c_* was the cell diameter, η was the dynamic viscosity of fluid, *E_c_* was the 2D Young's modulus of cell cortex, which was related to the bulk Young's modulus by *E_c_* = *E*_*c*,3D_ *h_c_* with *h_c_* denoting the thickness of cell cortex.

For the unit of length, given that the cell diameters for the DPD model and the experiment were *D*_*c*,DPD_ = 32 and *D*_*c*,EXP_ = 16 µm, respectively, we got [*L*] = 0.5 µm. To estimate the scales of force and time of DPD simulation, we first quantified fluid viscosity η_DPD_ = 1.31 and Young's modulus *E*_*c*,DPD_ = 115.47 of cell cortex, where the latter was related roughly to the bond rigidity *K_c_* of cell cortex via the relationship $E_{c,\mathrm{DPD}}=\frac{2}{\sqrt{3}}K_{c}$ [81]. Experimentally, multiple types of cancer cells are reported to be softer than healthy counterparts [82, 83]. By taking *E*_*c*,EXP_ = 10 µN/m (estimated from *E*_*c*,3D_ = 100 Pa,  *h_c_* = 0.1 µm) [82, 84] and η_EXP_ = 1.002 mPa · s, we obtained [*F*] = 0.043 pN and [*t*] = 4.42 ms. The DPD method usually set *r_c_* = 1 and *m_i_* = 1, that is, all the beads in the system had equal mass [73].

We converted any quantities in DPD units to physical units by multiplying [*L*],  [*F*],  [*t*] or their combinations. The parameters (in DPD units and physical units) used in the current study were summarized in Supplementary Table 2.

Simulation setup. To simulate the dynamic behavior of non‐adherent tumor cells under FSS, we constructed a rectangular fluid channel using two fixed parallel flat plates in the xy‐plane. The dimensions of the fluid channel in the x, y and z directions were 100, 50.2, and 47.6, respectively. The fixed plate consisted of four layers of beads arranged in a face‐centered cubic (FCC) lattice with a bead‐bead distance of 0.5. The fluid channel contained a non‐adherent tumor cell and a mass of fluid beads, wherein the tumor cell was initially placed in the center of the channel and the density of fluid bead was about 3.

The simulation box was a 100 × 50.2 × 50 rectangle subjected to periodic boundary conditions in x and y directions, and non‐periodic in the z direction, where two fixed parallel plates prevented beads from moving out of the fluid channel. The initial configuration of the model was packed together by PACKMOL and the simulations were performed after equilibrium is achieved (about 500 000 timesteps) [85]. The simulations were performed in the NVE ensemble by using the open‐source software LAMMPS (https://www.lammps.org) [86]. Particularly, the kinetics of the actin‐ and myosin‐based cytoskeleton were implemented using the LAMMPS built‐in “fix bond/react” module [87]. The time step of simulation Δ*t* =  0.01 and the time interval of reaction occurring δ*t* = 100Δ*t*  [73].

To drive shear flow, a driving force along the x‐direction was applied to the fluid beads. The driving force was uniform along the x and y directions and had a uniform gradient in the z direction, and linearly increased to a prescribed value from 0 within the initial 10 000 timesteps and then held constant for preventing dramatic deformation of the cell due to fluid shock. We thus obtained a parallel flow with a uniform velocity gradient $\frac{dv_{x}}{dz}$ (i.e., shear rate), by which FSS was applied to the non‐adherent tumor cell. The FSS was quantified as

(19) $$\tau =\eta \frac{dv_{x}}{dz}$$

In our simulations, shear stress varied from 0 to 10 dyne/cm^2^.

### 5.25 Calculation of Viscoelastic Moduli

The viscoelastic properties of the material were determined from the mean‐squared displacement (MSD) of probe particles using the local power‐law approximation proposed by Mason [88]. Briefly, the MSD, 〈Δ*r* ^2^(*t*)〉, was first calculated as a function of lag time *t*. Assuming that the MSD exhibits a local power‐law behavior over short time windows, that is, 〈Δ*r* ^2^(*t*)〉 ∼ *t^α^*, the local logarithmic slope $\alpha \left(t\right)=\frac{d\ln\langle \Delta r^{2}\left(t\right)\rangle }{d\ln t}$ was obtained from the derivative in log‐log coordinates.

Each angular frequency ω was mapped to the corresponding time scale *t* = 1/ω. The magnitude of the complex shear modulus was then estimated according to the generalized Stokes‐Einstein relation in its algebraic form

(20) $$\left|G^{*}\left(\omega \right)\right|=\frac{k_{B}T}{\pi a\;\langle \Delta r^{2}\left(1/\omega \right)\rangle \Gamma \left(1+\alpha \left(\omega \right)\right)}$$

where *k_B_* is the Boltzmann constant, *T* is the absolute temperature, *a* is the probe radius, and Γ denotes the Gamma function. The frequency‐dependent elastic (or storage) modulus and viscous (or loss) modulus were obtained from

(21) $$G^{\prime }\left(\omega \right)=\left|G^{*}\left(\omega \right)\right|\cos\left(\frac{\pi \alpha \left(\omega \right)}{2}\right)$$

and

(22) $$G^{\prime \prime }\left(\omega \right)=\left|G^{*}\left(\omega \right)\right|\sin\left(\frac{\pi \alpha \left(\omega \right)}{2}\right)$$

respectively. The dynamic viscosity is directly related to the viscous modulus by

(23) $$\eta \left(\omega \right)=\frac{G^{\prime \prime }\left(\omega \right)}{\omega }$$

### 5.26 The Experiments Using Patient‐Derived Primary Tumor Cells

Primary breast tumor cells were isolated from fresh human breast cancer tissues under the approval by the Institutional Review Boards of Jiangsu Province Hospital (Ethics approval #: 2024‐SR‐982) and Hong Kong Polytechnic University (Ethics approval #: HSEARS20230913001). For the isolation of primary tumor cells, human breast cancer tissue was washed by PBS (HyClone) for twice, followed by adipose tissue removal. The tissue was then minced into approximately 1 mm^3^ pieces and digested by the mixture of Dnase (Gibco) and collagenase type I (Sigma‐Aldrich) in 1:30 ratio (v/v) at 37°C for 4 h to remove fibroblasts. Further, the breast tissue was treated with primary breast tumor cell culture medium (Pricella Biotechnology Co., Ltd, Cat. No CM‐H146) to terminate tissue digestion. Finally, primary breast tumor cells were collected by subsequent centrifugation and identified by vimentin (Affinity, Cat. No BF8006) staining. The primary cells were tested negative for HIV‐1, HBV, HCV, mycoplasma, and bacteria. Primary cells within five passages were used in this study. All the experimental procedures using primary breast tumor cells were approved by the Institutional Review Boards of Jiangsu Province Hospital (Ethics approval #: 2024‐SR‐982) and Hong Kong Polytechnic University (Ethics approval #: HSEARS20230913001).

### 5.27 Animal Experiments

All animal experiments in this study were on the record of the animal license ((22‐321) DH/HT&A/8/2/4 Pt.12) issued by Department of Health, Hong Kong Special Administrative Region. The experimental protocols were approved by the Animal Subjects Ethics Subcommittee of Hong Kong Polytechnic University (Ethics approval #: 23‐24/671‐BME‐R‐CRF). Following the National Institutes of Health's Guide for the Care and Use of Laboratory Animals, all animal experiments were under the supervision of Centralised Animal Facilities of Hong Kong Polytechnic University. Humanitarian care was strictly followed to relief the pain of the mice during all the experiments and terminate them humanely after the experiments.

Measurement of CTC apoptosis in vasculature in vivo: MDA‐MB‐231 cells were stably transfected with pNLuc plasmid. These cells were either pretreated with Y27632 or DMSO for an hour, or transfected with doxycycline‐inducible ROCK2 shRNA (Dox‐shROCK2) following by the treatment of DMSO or doxycycline for 2 days. 100 µL of cell solution containing 1 × 10^6^ pre‐treated MDA‐MB‐231 cells was injected into the tail vein of 5‐week female nude mice (Centralised Animal Facilities of Hong Kong Polytechnic University). After 12‐h circulation, the mice were anesthetized by isoflurane and the whole blood (∼1 mL) was retrieved into a vacuum blood collection tube (Lingen Precision Medical Products Co., Ltd.). Before bioluminescence imaging, the blood was transferred to a 12‐well plate and 20 µM coelenterazine (Sigma‐Aldrich) was added to each well in 1:4 ratio (v/v) and incubated for 15 min in dark. Finally, bioluminescence imaging was performed using Perkin‐Elmer IVIS Lumina Series III Pre‐Clinical In Vivo Animal Imaging Systems.

Long‐term formation of metastatic tumors: MDA‐MB‐231 cells were transfected with doxycycline‐inducible ROCK2 shRNA and pretreated with DMSO or doxycycline for 2 days. For each condition, 1 × 10^6^ cells were injected into the tail vein of female nude mice. Bioluminescence signal was taken 15 min after injection (week 0) and once every week after cell inoculation. The bioluminescence signal of each animal was normalized to the signal of week 0. Bioluminescence imaging was performed using Perkin‐Elmer IVIS Lumina Series III Pre‐Clinical In Vivo Animal Imaging Systems.

### 5.28 Statistical Analysis

Data were presented as mean ± SEM (standard error of the mean), except for the modeling results in Figure 4, where mean ± SD (standard deviation) was adopted. For each experiment, at least three repeats were performed. For comparison between two groups, unpaired two‐tailed student's *t*‐test (if samples assumed normal distribution) or Mann–Whitney U‐test (if samples did not assume normality) was used. For comparison between three or more groups, one‐way analysis of variance (ANOVA) followed by Tukey's post‐hoc test (if samples assumed normal distribution) and Kruskal–Wallis one‐way ANOVA followed by Mann–Whitney U‐test with Bonferroni correction (if samples did not assume normality) were used. *p* value lower than 0.05 was considered statistically significant. *, *p* < 0.05, **, *p* < 0.01, ***, *p* < 0.001.

## Author Contributions

Y.T. conceived the project. Y.T. and C.Z. designed the experiments in this project. C.Z., K.L., G.H., Y.X., K.T., B.H., P.D., and R.M. conducted the experiments and analyzed the data. Q.W. and B.J. developed the theoretical model and analyzed the simulation results, Y.T., B.J., C.Z., and Q.W. wrote the manuscript. Y.T. and B.J. supervised the study. All authors commented and approved the manuscript.

## Conflicts of Interest

The authors declare no conflict of interest.

## Supporting information




**Supporting File**: advs75023‐sup‐0001‐SuppMat.pdf.

## Data Availability

The data that support the findings of this study are available from the corresponding author upon reasonable request.
