# Supplementary material for: Mechanoadaptation via Myosin Cytoplasmic Redistribution Protects Circulating Tumor Cells From Shear‐induced Death During Hematogenous Dissemination
Source: Adv Sci (Weinh). 2026 Mar 30;13(33):e23112. doi: 10.1002/advs.202523112 (PMC13271613; doi:10.1002/advs.202523112)
Supplement: Supplementary file 1 — Supporting File: advs75023‐sup‐0001‐SuppMat.pdf. [file ADVS-13-e23112-s001.pdf]

Fig S1

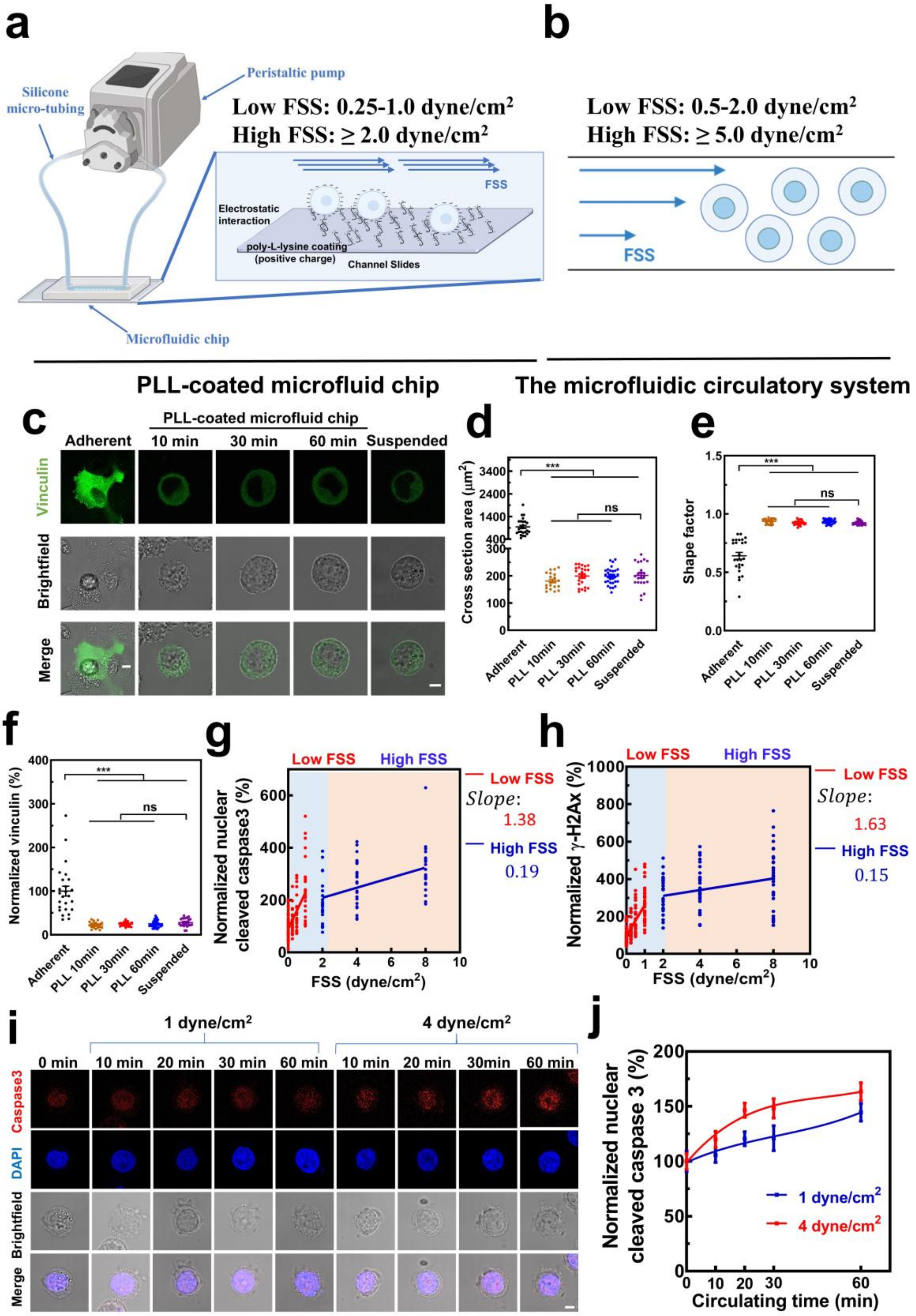

a
Peristaltic pump
Silicone micro-tubing
Low FSS: 0.25-1.0 dyne/cm2
High FSS: ≥ 2.0 dyne/cm2
FSS
Electrostatic interaction
poly-L-lysine coating (positive charge)
Channel Slides
Microfluidic chip
PLL-coated microfluid chip
b
Low FSS: 0.5-2.0 dyne/cm2
High FSS: ≥ 5.0 dyne/cm2
FSS
The microfluidic circulatory system
c
PLL-coated microfluid chip
Adherent
10 min
30 min
60 min
Suspended
Vinculin
Brightfield
Merge
d
Cross section area (μm2)
Adherent
PLL 10min
PLL 30min
PLL 60min
Suspended
ns
e
Shape factor
ns
f
Normalized vinculin (%)
ns
g
Low FSS
High FSS
Normalized nuclear cleaved caspase3 (%)
FSS (dyne/cm2)
Slope:
1.38
0.19
h
Normalized γ-H2Ax (%)
Slope:
1.63
0.15
i
1 dyne/cm2
4 dyne/cm2
0 min
10 min
20 min
30 min
60 min
30min
Caspase3
DAPI
j
Normalized nuclear cleaved caspase 3 (%)
Circulating time (min)

Fig S2

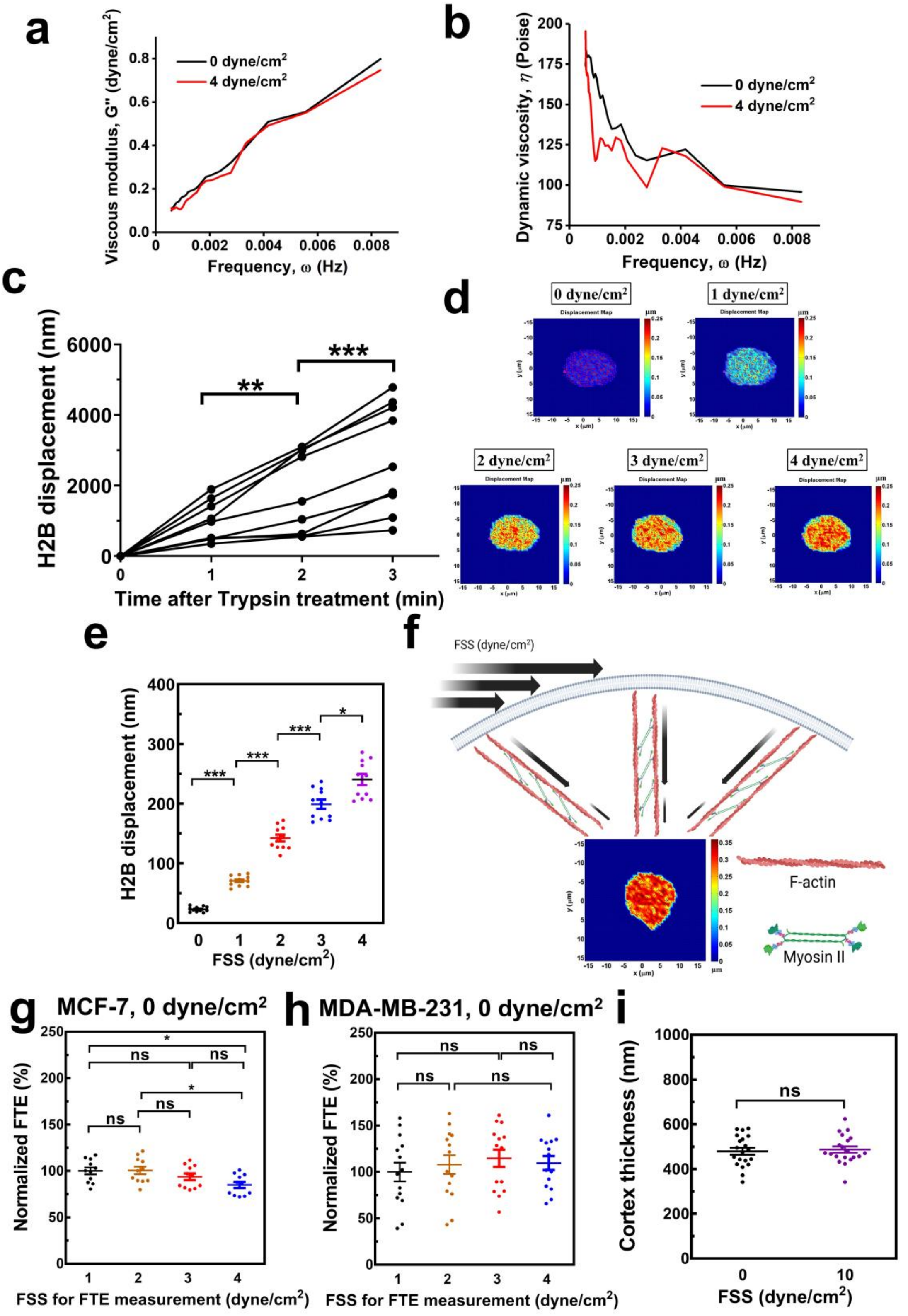
a
Viscous modulus, G'' (dyne/cm2)
Frequency, ω (Hz)
0 dyne/cm2
4 dyne/cm2
b
Dynamic viscosity, η (Poise)
Frequency, ω (Hz)
0 dyne/cm2
4 dyne/cm2
c
H2B displacement (nm)
Time after Trypsin treatment (min)
**
***
d
0 dyne/cm2
1 dyne/cm2
2 dyne/cm2
3 dyne/cm2
4 dyne/cm2
Displacement Map
e
H2B displacement (nm)
FSS (dyne/cm2)
f
FSS (dyne/cm2)
F-actin
Myosin II
g
MCF-7, 0 dyne/cm2
Normalized FTE (%)
FSS for FTE measurement (dyne/cm2)
h
MDA-MB-231, 0 dyne/cm2
Normalized FTE (%)
FSS for FTE measurement (dyne/cm2)
i
Cortex thickness (nm)
FSS (dyne/cm2)
ns

Fig S3

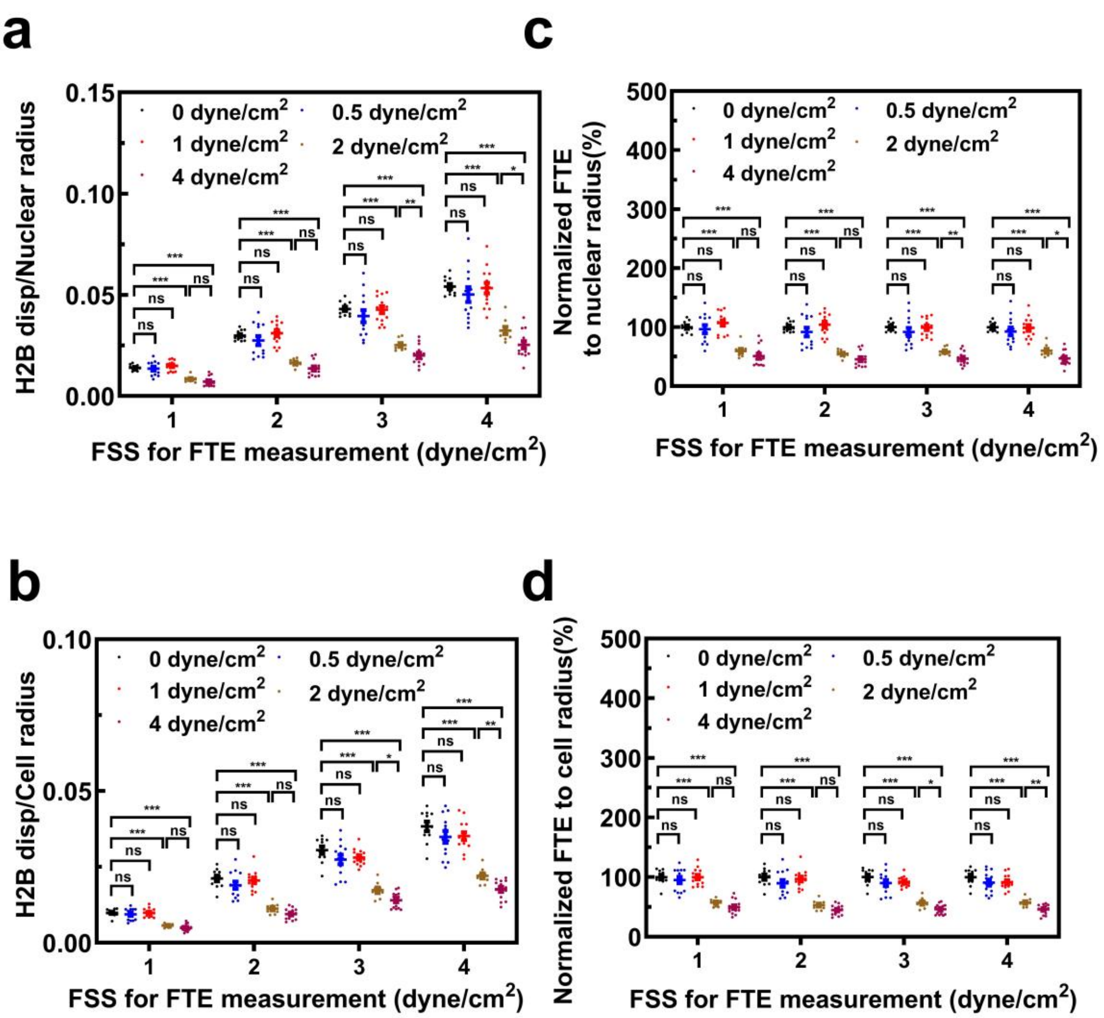
a
H2B disp/Nuclear radius
0 dyne/cm2
0.5 dyne/cm2
1 dyne/cm2
2 dyne/cm2
4 dyne/cm2
FSS for FTE measurement (dyne/cm2)
b
H2B disp/Cell radius
c
Normalized FTE to nuclear radius(%)
d
Normalized FTE to cell radius(%)

Fig S4

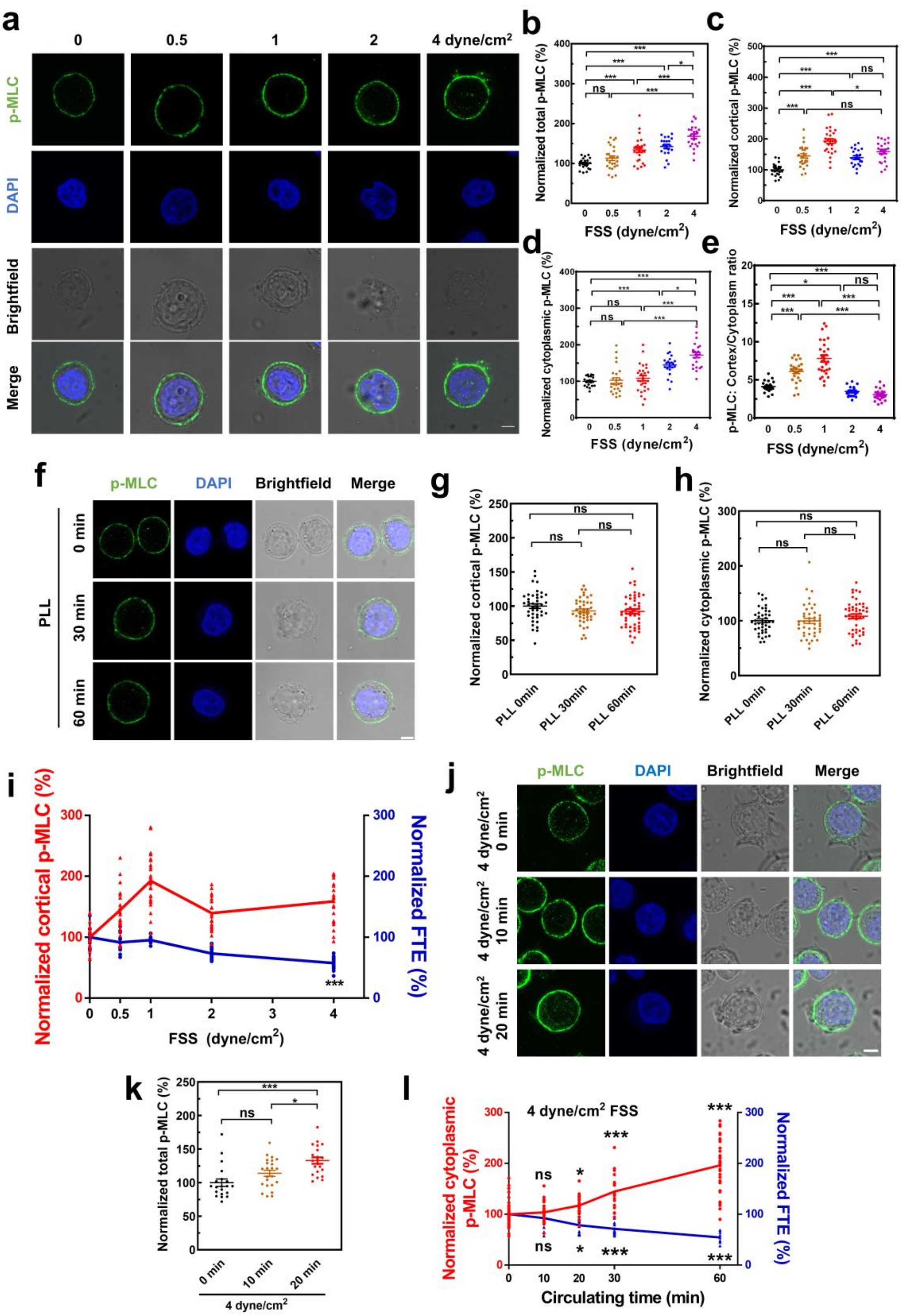
a
0
0.5
1
2
4 dyne/cm²
p-MLC
DAPI
Brightfield
Merge
b
Normalized total p-MLC (%)
FSS (dyne/cm²)
c
Normalized cortical p-MLC (%)
FSS (dyne/cm²)
d
Normalized cytoplasmic p-MLC (%)
FSS (dyne/cm²)
e
p-MLC: Cortex/Cytoplasm ratio
FSS (dyne/cm²)
f
p-MLC
DAPI
Brightfield
Merge
PLL
0 min
30 min
60 min
g
Normalized cortical p-MLC (%)
PLL 0min
PLL 30min
PLL 60min
h
Normalized cytoplasmic p-MLC (%)
PLL 0min
PLL 30min
PLL 60min
i
Normalized cortical p-MLC (%)
Normalized FTE (%)
FSS (dyne/cm²)
j
p-MLC
DAPI
Brightfield
Merge
4 dyne/cm² 0 min
4 dyne/cm² 10 min
4 dyne/cm² 20 min
k
Normalized total p-MLC (%)
0 min
10 min
20 min
4 dyne/cm²
l
4 dyne/cm² FSS
Normalized cytoplasmic p-MLC (%)
Normalized FTE (%)
Circulating time (min)

Fig S5

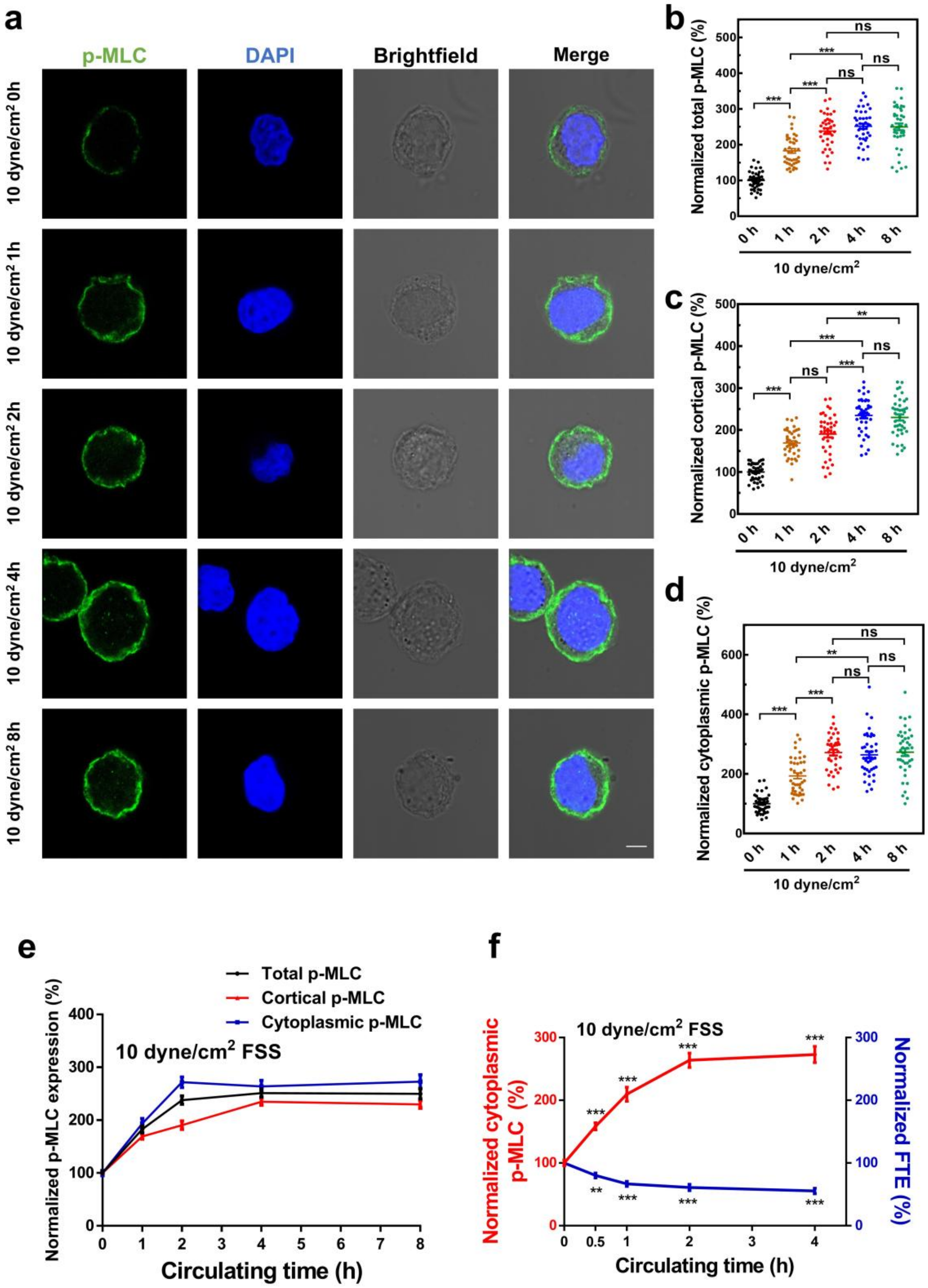
a
p-MLC
DAPI
Brightfield
Merge
10 dyne/cm² 0h
10 dyne/cm² 1h
10 dyne/cm² 2h
10 dyne/cm² 4h
10 dyne/cm² 8h
b
Normalized total p-MLC (%)
10 dyne/cm²
c
Normalized cortical p-MLC (%)
d
Normalized cytoplasmic p-MLC (%)
e
Total p-MLC
Cortical p-MLC
Cytoplasmic p-MLC
10 dyne/cm² FSS
Normalized p-MLC expression (%)
Circulating time (h)
f
Normalized cytoplasmic p-MLC (%)
Normalized FTE (%)

Fig S6

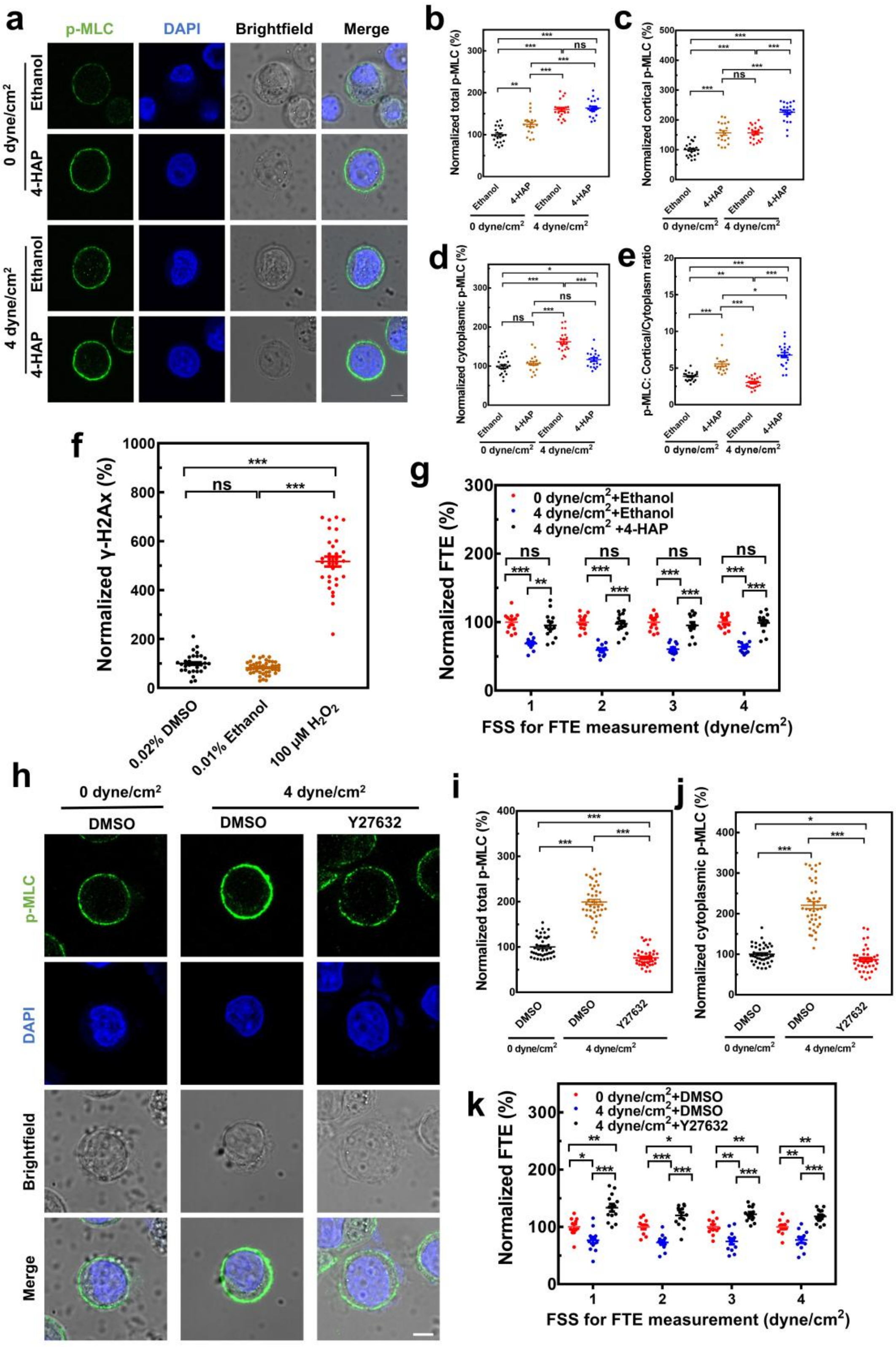

a
p-MLC
DAPI
Brightfield
Merge
0 dyne/cm²
Ethanol
4-HAP
4 dyne/cm²
Ethanol
4-HAP
b
Normalized total p-MLC (%)
c
Normalized cortical p-MLC (%)
d
Normalized cytoplasmic p-MLC (%)
e
p-MLC: Cortical/Cytoplasm ratio
Ethanol
4-HAP
0 dyne/cm²
4 dyne/cm²
f
Normalized γ-H2Ax (%)
0.02% DMSO
0.01% Ethanol
100 μM H2O2
g
0 dyne/cm²+Ethanol
4 dyne/cm²+Ethanol
4 dyne/cm² +4-HAP
Normalized FTE (%)
FSS for FTE measurement (dyne/cm²)
h
0 dyne/cm²
4 dyne/cm²
DMSO
Y27632
p-MLC
DAPI
Brightfield
Merge
i
Normalized total p-MLC (%)
j
Normalized cytoplasmic p-MLC (%)
k
0 dyne/cm²+DMSO
4 dyne/cm²+DMSO
4 dyne/cm²+Y27632
Normalized FTE (%)
FSS for FTE measurement (dyne/cm²)

Fig S7

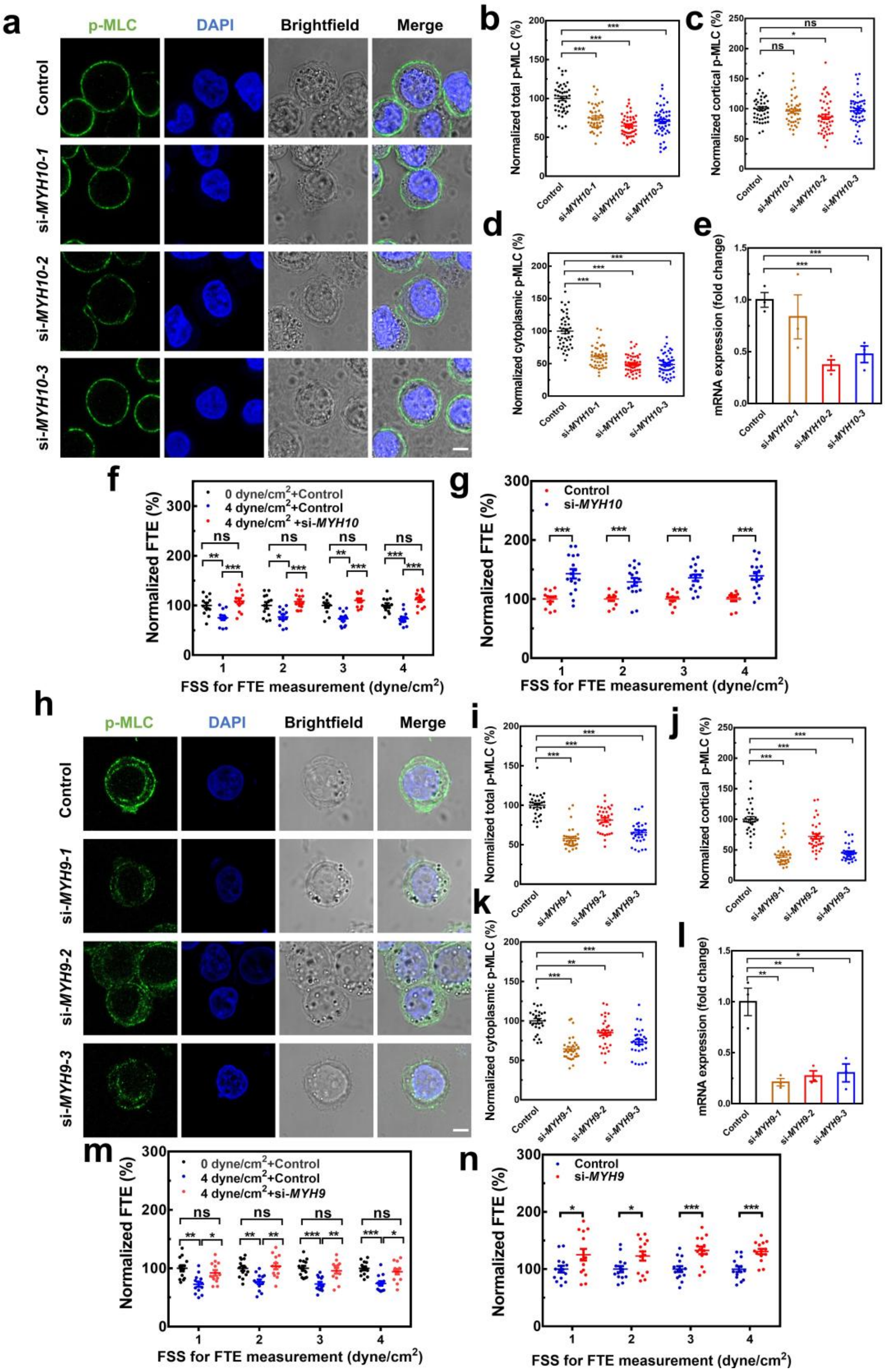

a
p-MLC
DAPI
Brightfield
Merge
Control
si-MYH10-1
si-MYH10-2
si-MYH10-3
b
Normalized total p-MLC (%)
c
Normalized cortical p-MLC (%)
d
Normalized cytoplasmic p-MLC (%)
e
mRNA expression (fold change)
f
0 dyne/cm2+Control
4 dyne/cm2+Control
4 dyne/cm2 +si-MYH10
Normalized FTE (%)
FSS for FTE measurement (dyne/cm2)
g
Control
si-MYH10
h
si-MYH9-1
si-MYH9-2
si-MYH9-3
i
j
k
l
m
4 dyne/cm2+si-MYH9
n
si-MYH9

Fig S8

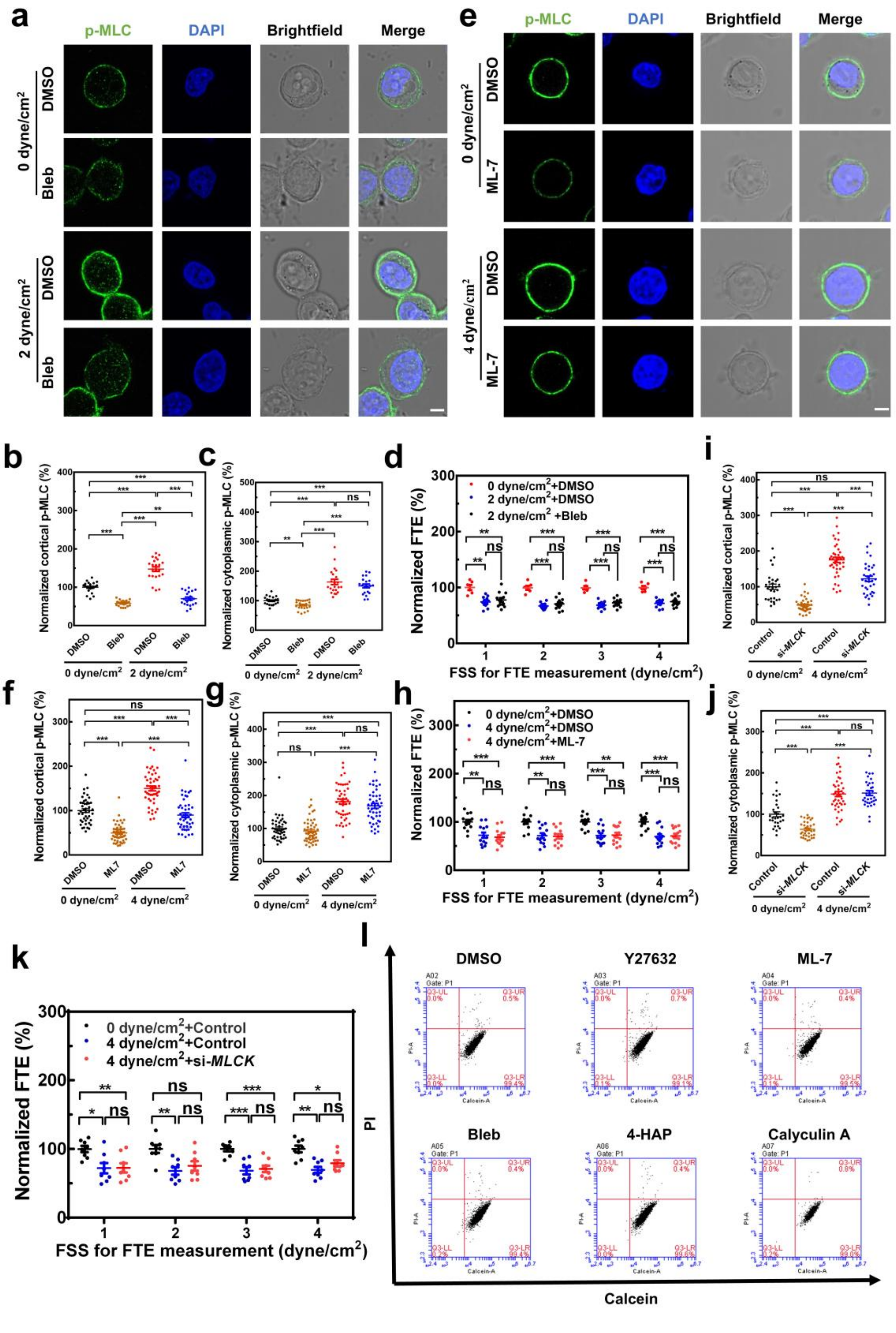

a
p-MLC
DAPI
Brightfield
Merge
0 dyne/cm2
DMSO
Bleb
2 dyne/cm2
DMSO
Bleb
e
p-MLC
DAPI
Brightfield
Merge
0 dyne/cm2
DMSO
ML-7
4 dyne/cm2
DMSO
ML-7
b
Normalized cortical p-MLC (%)
DMSO
Bleb
DMSO
Bleb
0 dyne/cm2
2 dyne/cm2
c
Normalized cytoplasmic p-MLC (%)
d
Normalized FTE (%)
0 dyne/cm2+DMSO
2 dyne/cm2+DMSO
2 dyne/cm2 +Bleb
FSS for FTE measurement (dyne/cm2)
i
Normalized cortical p-MLC (%)
Control
si-MLCK
Control
si-MLCK
0 dyne/cm2
4 dyne/cm2
f
Normalized cortical p-MLC (%)
DMSO
ML7
DMSO
ML7
0 dyne/cm2
4 dyne/cm2
g
Normalized cytoplasmic p-MLC (%)
h
Normalized FTE (%)
0 dyne/cm2+DMSO
4 dyne/cm2+DMSO
4 dyne/cm2+ML-7
FSS for FTE measurement (dyne/cm2)
j
Normalized cytoplasmic p-MLC (%)
k
Normalized FTE (%)
0 dyne/cm2+Control
4 dyne/cm2+Control
4 dyne/cm2+si-MLCK
FSS for FTE measurement (dyne/cm2)
l
DMSO
Y27632
ML-7
Bleb
4-HAP
Calyculin A
PI
Calcein

Fig S9

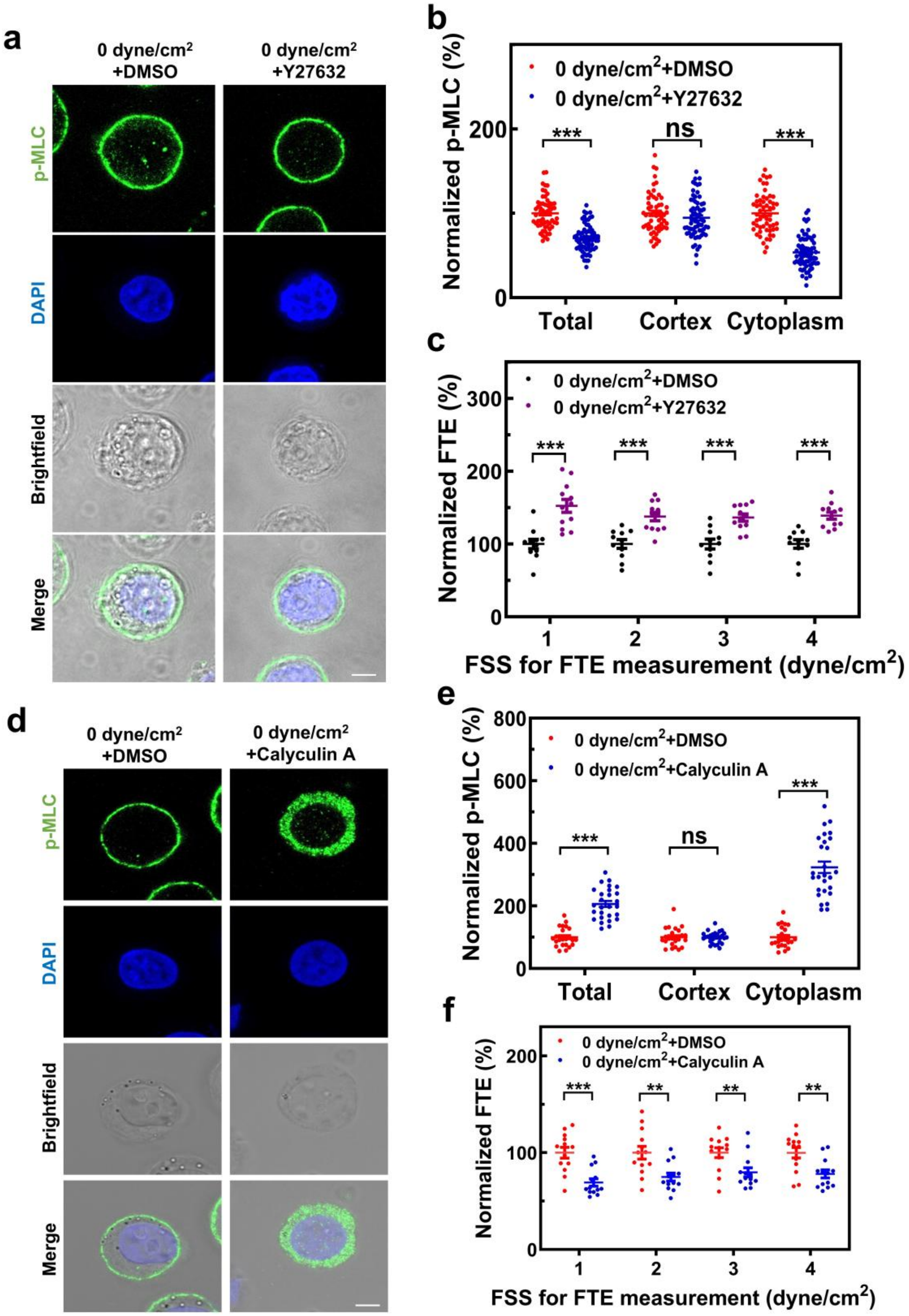

a
0 dyne/cm2 +DMSO
0 dyne/cm2 +Y27632
p-MLC
DAPI
Brightfield
Merge
b
Normalized p-MLC (%)
0 dyne/cm2+DMSO
0 dyne/cm2+Y27632
***
ns
***
Total
Cortex
Cytoplasm
c
Normalized FTE (%)
0 dyne/cm2+DMSO
0 dyne/cm2+Y27632
FSS for FTE measurement (dyne/cm2)
d
0 dyne/cm2 +DMSO
0 dyne/cm2 +Calyculin A
p-MLC
DAPI
Brightfield
Merge
e
Normalized p-MLC (%)
0 dyne/cm2+DMSO
0 dyne/cm2+Calyculin A
Total
Cortex
Cytoplasm
f
Normalized FTE (%)
0 dyne/cm2+DMSO
0 dyne/cm2+Calyculin A
FSS for FTE measurement (dyne/cm2)

Fig S10

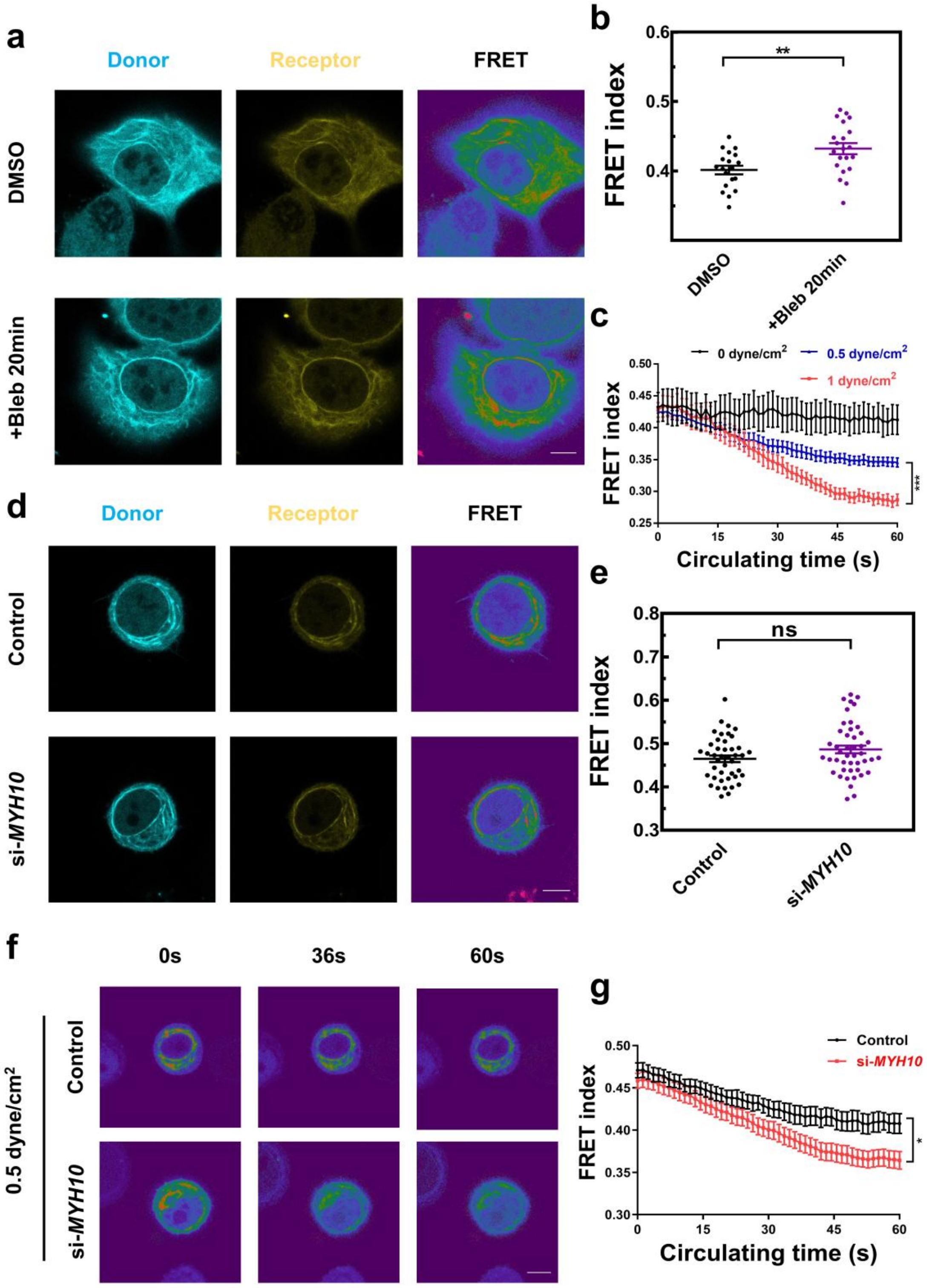
a
Donor
Receptor
FRET
DMSO
+Bleb 20min
b
FRET index
**
DMSO
+Bleb 20min
c
0 dyne/cm2
0.5 dyne/cm2
1 dyne/cm2
FRET index
***
Circulating time (s)
d
Donor
Receptor
FRET
Control
si-MYH10
e
FRET index
ns
Control
si-MYH10
f
0s
36s
60s
0.5 dyne/cm2
Control
si-MYH10
g
Control
si-MYH10
FRET index
*
Circulating time (s)

Fig S11

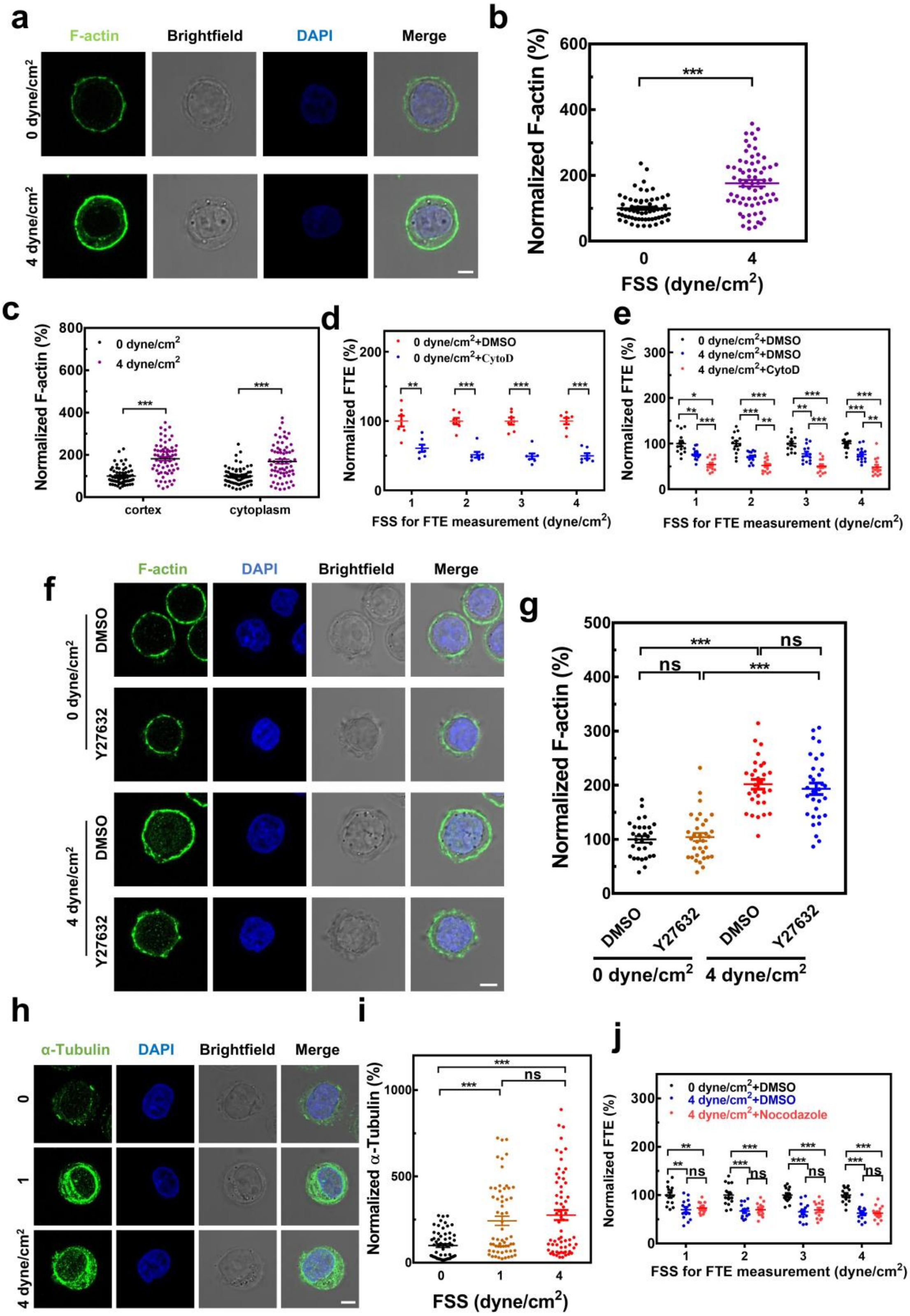
a
F-actin
Brightfield
DAPI
Merge
0 dyne/cm2
4 dyne/cm2
b
Normalized F-actin (%)
FSS (dyne/cm2)
c
0 dyne/cm2
4 dyne/cm2
cortex
cytoplasm
d
Normalized FTE (%)
0 dyne/cm2+DMSO
0 dyne/cm2+CytoD
FSS for FTE measurement (dyne/cm2)
e
0 dyne/cm2+DMSO
4 dyne/cm2+DMSO
4 dyne/cm2+CytoD
f
DMSO
Y27632
g
ns
h
α-Tubulin
i
Normalized α-Tubulin (%)
j
4 dyne/cm2+Nocodazole

Fig S12

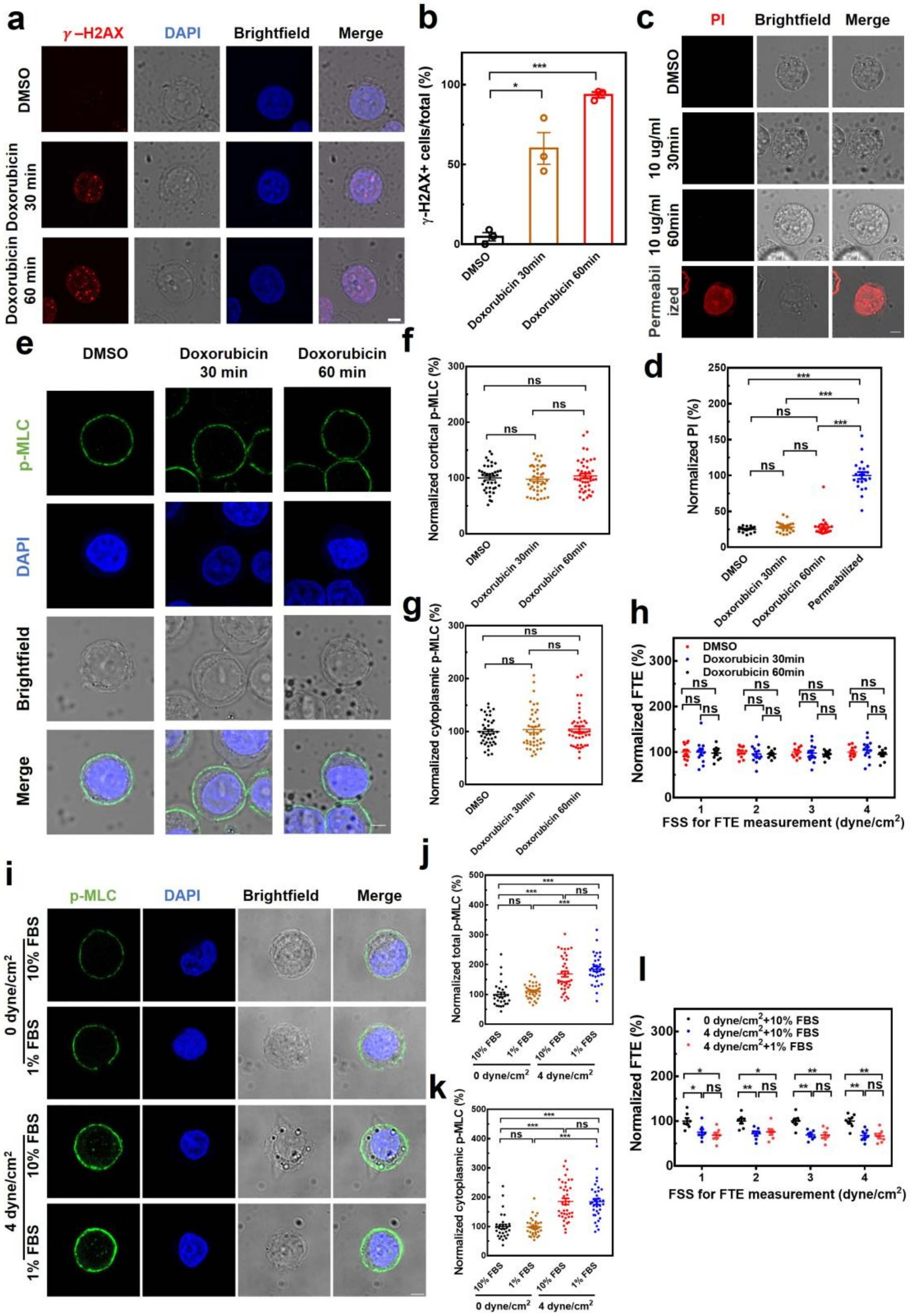
a
γ –H2AX
DAPI
Brightfield
Merge
DMSO
Doxorubicin 30 min
Doxorubicin 60 min
b
γ-H2AX+ cells/total (%)
DMSO
Doxorubicin 30min
Doxorubicin 60min
c
PI
Brightfield
Merge
DMSO
10 ug/ml 30min
10 ug/ml 60min
Permeabilized
d
Normalized PI (%)
Permeabilized
e
p-MLC
f
Normalized cortical p-MLC (%)
g
Normalized cytoplasmic p-MLC (%)
h
Normalized FTE (%)
FSS for FTE measurement (dyne/cm²)
i
0 dyne/cm²
4 dyne/cm²
10% FBS
1% FBS
j
Normalized total p-MLC (%)
k
l
0 dyne/cm²+10% FBS
4 dyne/cm²+10% FBS
4 dyne/cm²+1% FBS

Fig S13

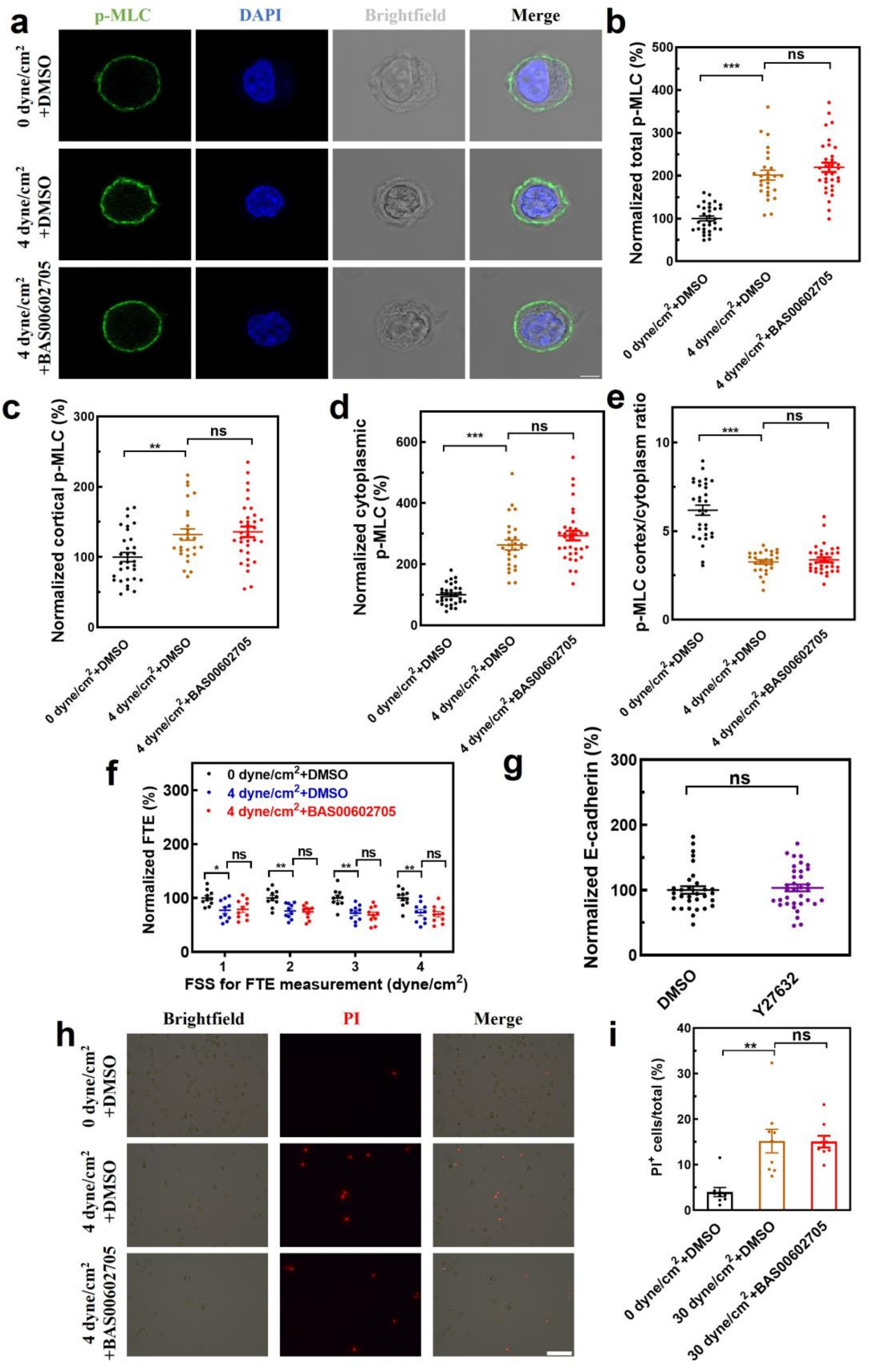
a
p-MLC
DAPI
Brightfield
Merge
0 dyne/cm2 +DMSO
4 dyne/cm2 +DMSO
4 dyne/cm2 +BAS00602705
b
Normalized total p-MLC (%)
***
ns
0 dyne/cm2+DMSO
4 dyne/cm2+DMSO
4 dyne/cm2+BAS00602705
c
Normalized cortical p-MLC (%)
**
ns
d
Normalized cytoplasmic p-MLC (%)
***
ns
e
p-MLC cortex/cytoplasm ratio
***
ns
f
Normalized FTE (%)
0 dyne/cm2+DMSO
4 dyne/cm2+DMSO
4 dyne/cm2+BAS00602705
FSS for FTE measurement (dyne/cm2)
g
Normalized E-cadherin (%)
ns
DMSO
Y27632
h
Brightfield
PI
Merge
0 dyne/cm2 +DMSO
4 dyne/cm2 +DMSO
4 dyne/cm2 +BAS00602705
i
PI+ cells/total (%)
**
ns
0 dyne/cm2+DMSO
30 dyne/cm2+DMSO
30 dyne/cm2+BAS00602705

Fig S14

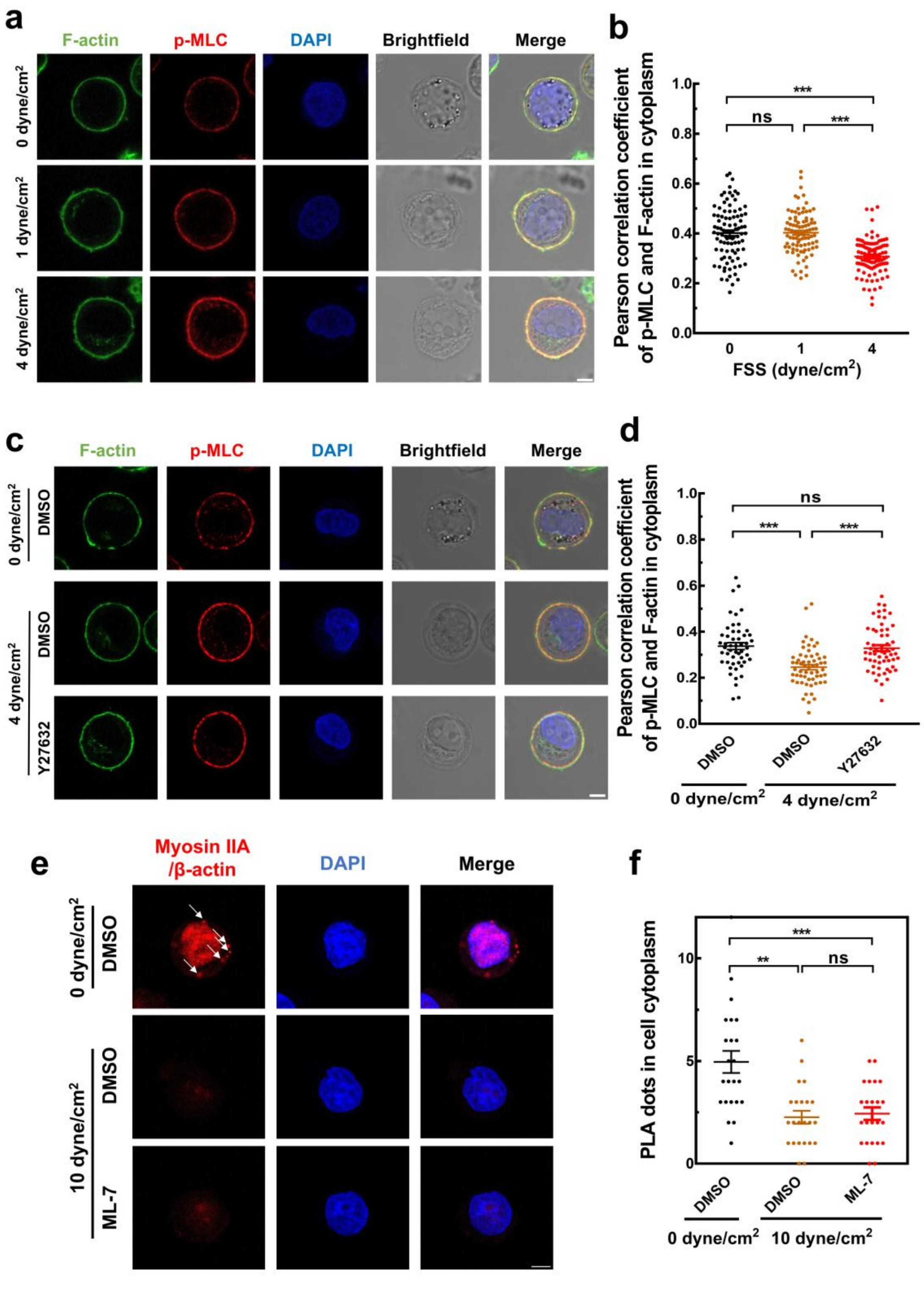
a
F-actin
p-MLC
DAPI
Brightfield
Merge
0 dyne/cm²
1 dyne/cm²
4 dyne/cm²
b
Pearson correlation coefficient of p-MLC and F-actin in cytoplasm
FSS (dyne/cm²)
ns
***
c
DMSO
Y27632
0 dyne/cm²
4 dyne/cm²
d
e
Myosin IIA /β-actin
10 dyne/cm²
ML-7
f
PLA dots in cell cytoplasm
**

Fig S15

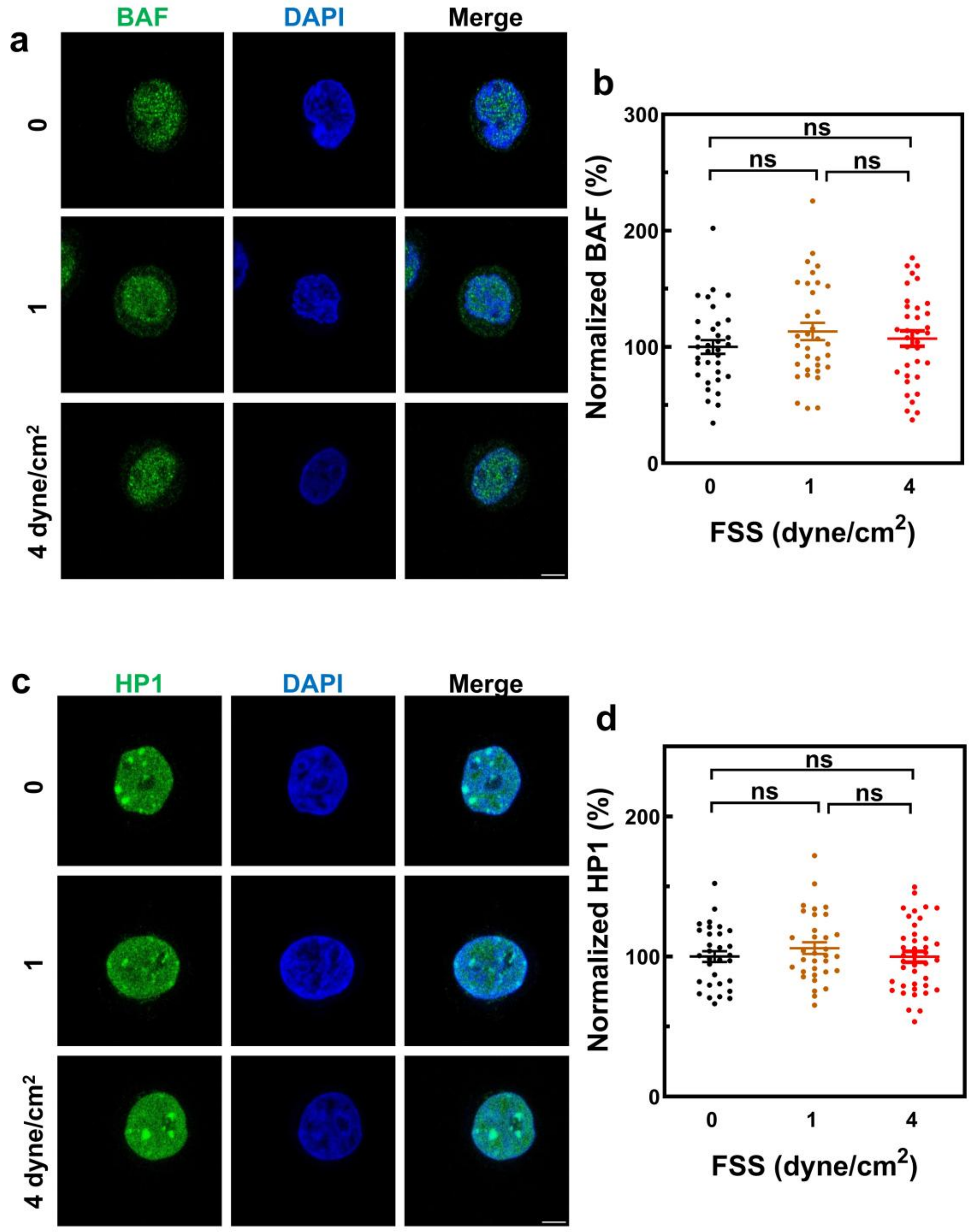
a
BAF
DAPI
Merge
0
1
4 dyne/cm²
b
Normalized BAF (%)
300
200
100
0
ns
ns
ns
0
1
4
FSS (dyne/cm²)
c
HP1
DAPI
Merge
0
1
4 dyne/cm²
d
Normalized HP1 (%)
200
100
0
ns
ns
ns
0
1
4
FSS (dyne/cm²)

Fig S16

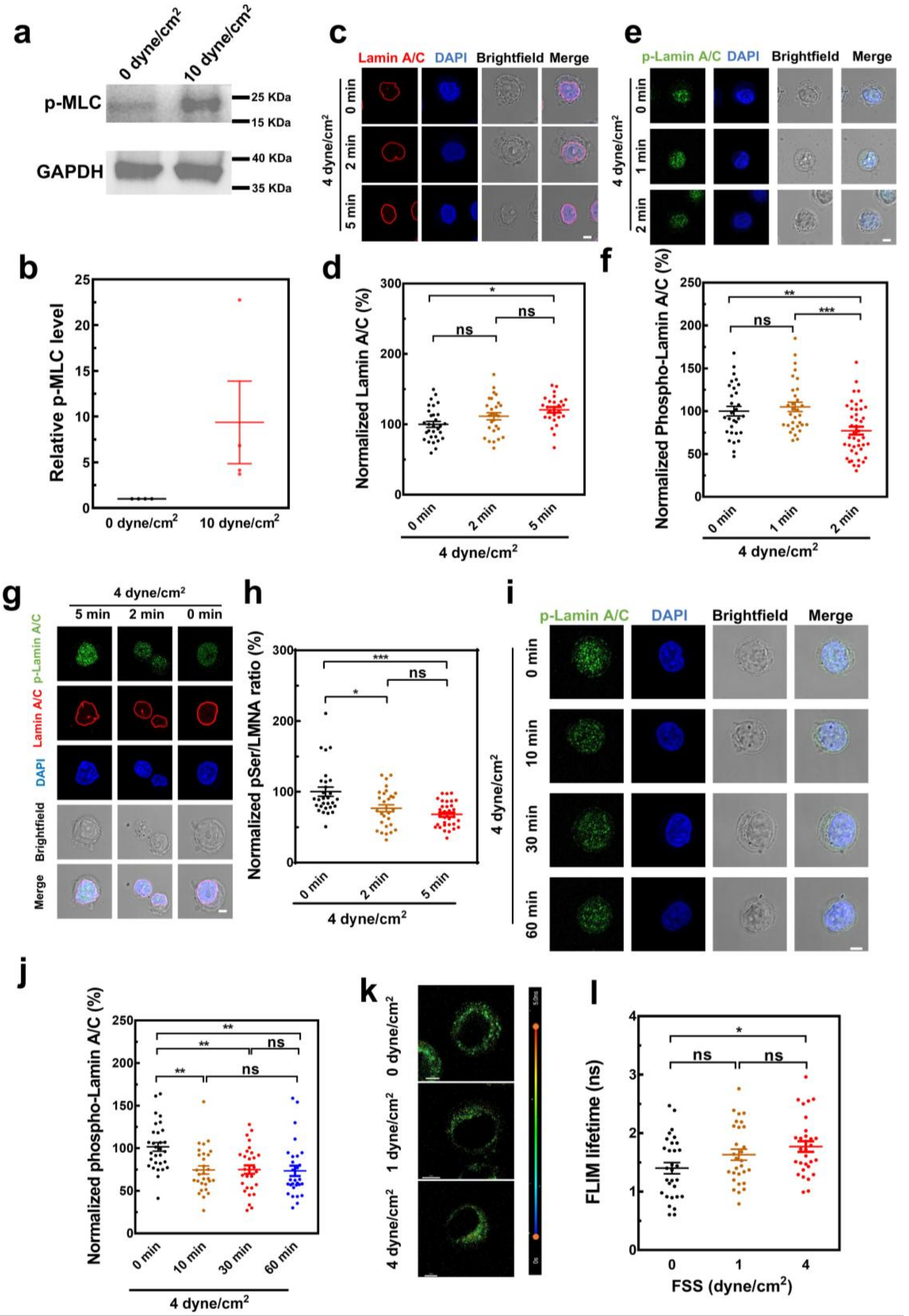

a
0 dyne/cm2
10 dyne/cm2
p-MLC
25 KDa
15 KDa
GAPDH
40 KDa
35 KDa
b
Relative p-MLC level
0 dyne/cm2
10 dyne/cm2
c
Lamin A/C
DAPI
Brightfield
Merge
4 dyne/cm2
0 min
2 min
5 min
d
Normalized Lamin A/C (%)
ns
e
p-Lamin A/C
DAPI
Brightfield
Merge
0 min
1 min
2 min
f
Normalized Phospho-Lamin A/C (%)
g
4 dyne/cm2
5 min
2 min
0 min
p-Lamin A/C
Lamin A/C
DAPI
Brightfield
Merge
h
Normalized pSer/LMNA ratio (%)
i
p-Lamin A/C
DAPI
Brightfield
Merge
0 min
10 min
30 min
60 min
j
Normalized phospho-Lamin A/C (%)
k
0 dyne/cm2
1 dyne/cm2
4 dyne/cm2
l
FLIM lifetime (ns)
FSS (dyne/cm2)

Fig S17

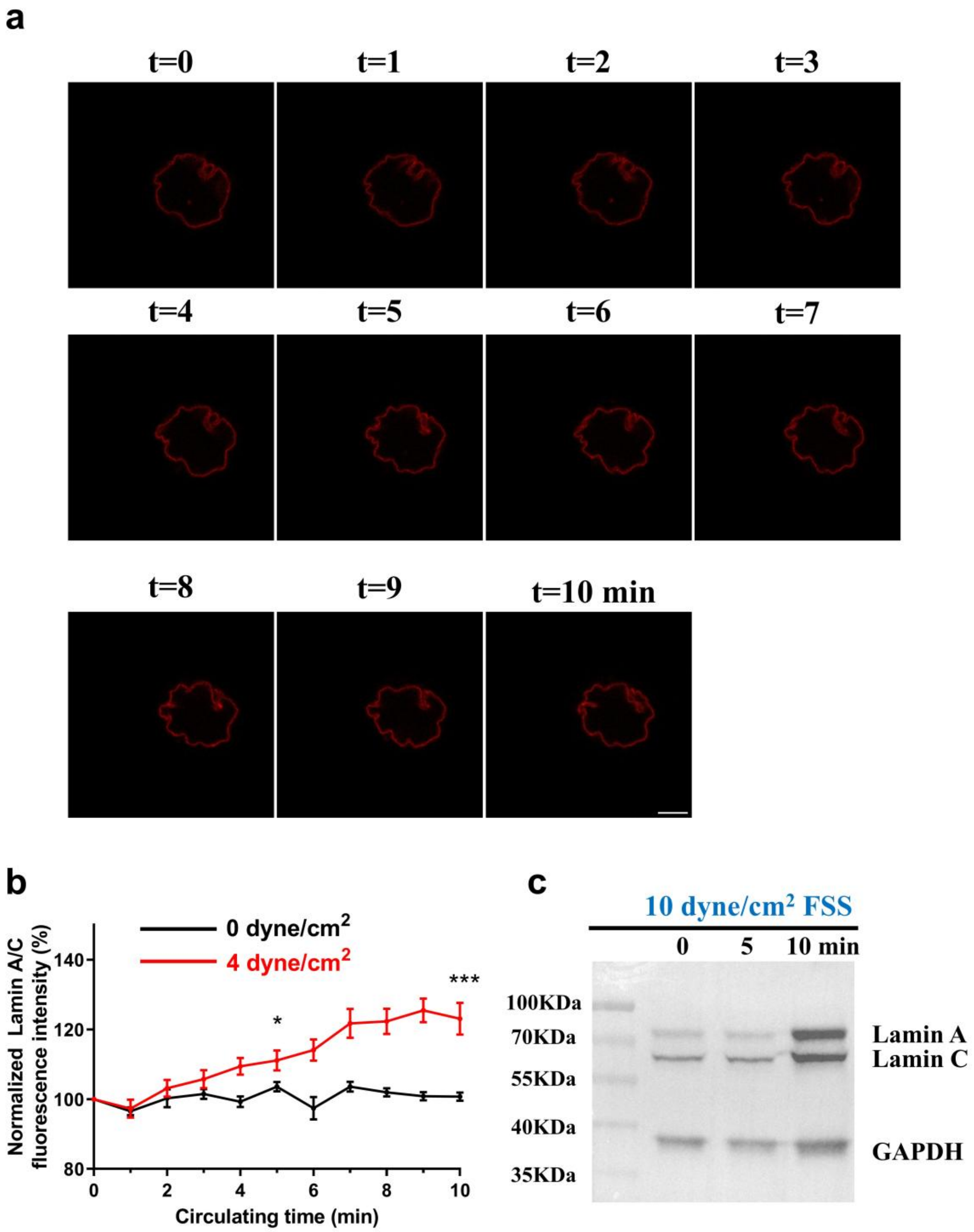
a
t=0
t=1
t=2
t=3
t=4
t=5
t=6
t=7
t=8
t=9
t=10 min
b
0 dyne/cm2
4 dyne/cm2
Normalized Lamin A/C fluorescence intensity (%)
Circulating time (min)
*
***
80
100
120
140
0
2
4
6
8
10
c
10 dyne/cm2 FSS
0
5
10 min
100KDa
70KDa
55KDa
40KDa
35KDa
Lamin A
Lamin C
GAPDH

Fig S18

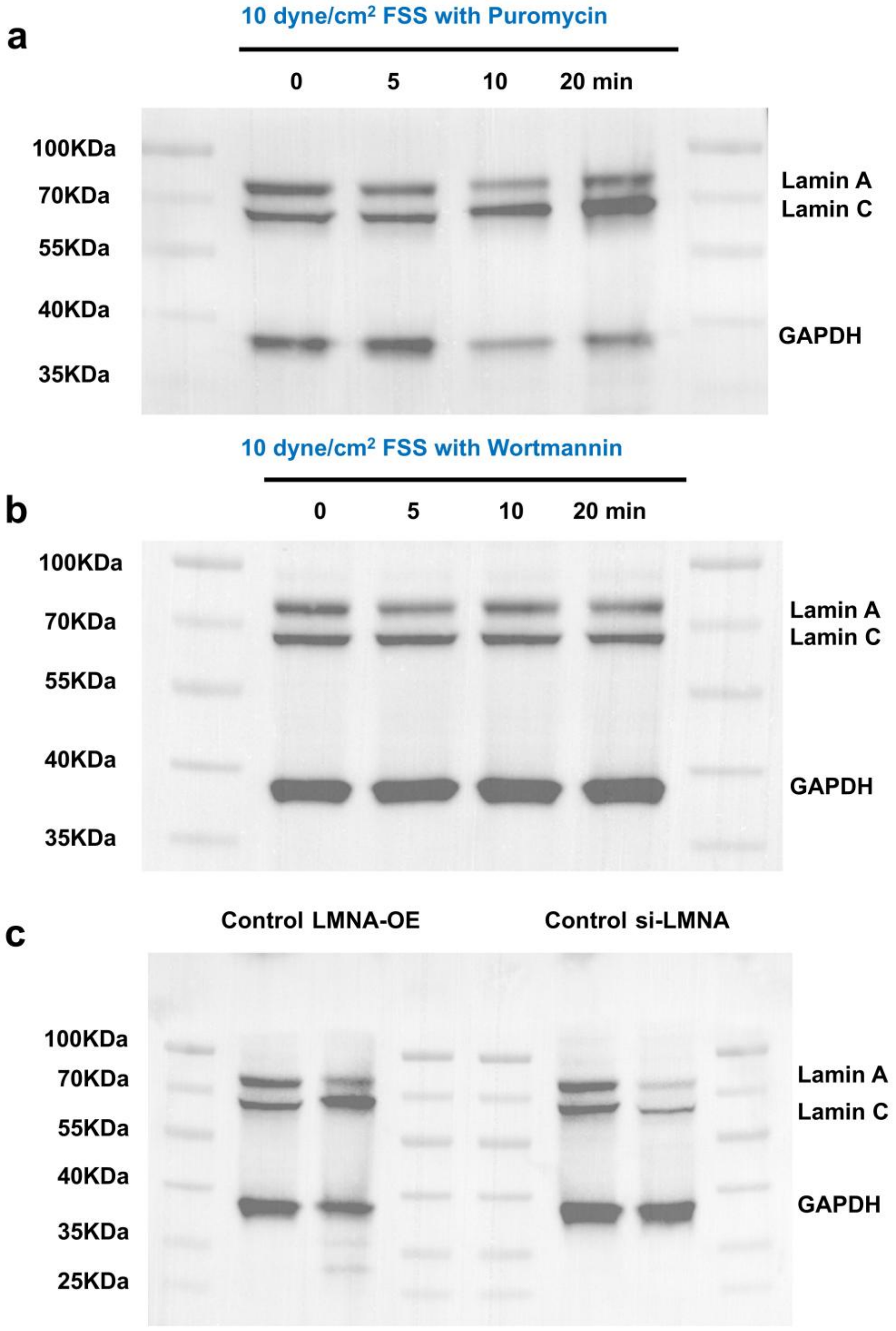
a
10 dyne/cm2 FSS with Puromycin
0
5
10
20 min
100KDa
70KDa
55KDa
40KDa
35KDa
Lamin A
Lamin C
GAPDH
b
10 dyne/cm2 FSS with Wortmannin
0
5
10
20 min
100KDa
70KDa
55KDa
40KDa
35KDa
Lamin A
Lamin C
GAPDH
c
Control LMNA-OE
Control si-LMNA
100KDa
70KDa
55KDa
40KDa
35KDa
25KDa
Lamin A
Lamin C
GAPDH

Fig S19

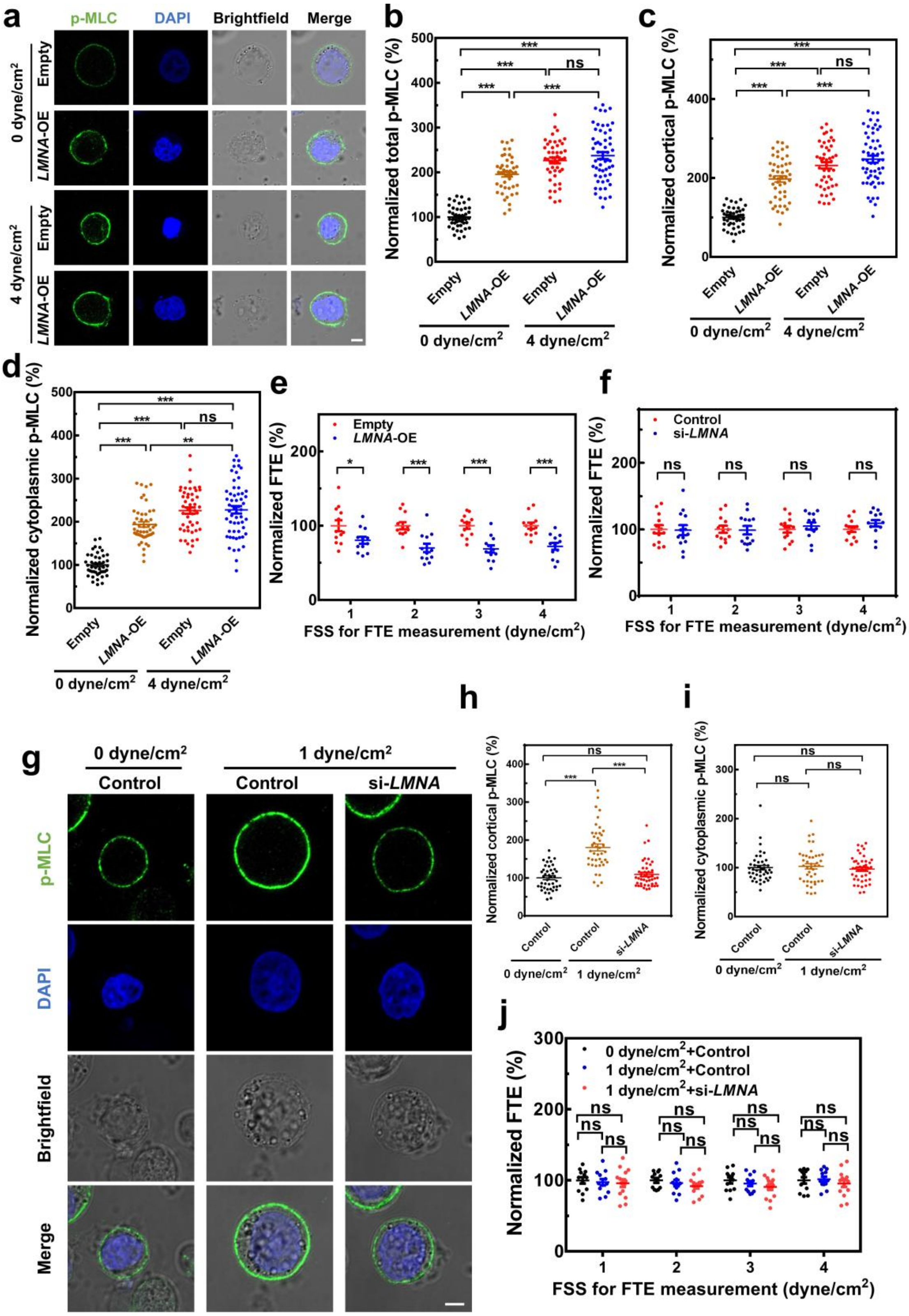
a
p-MLC
DAPI
Brightfield
Merge
0 dyne/cm2
4 dyne/cm2
Empty
LMNA-OE
b
Normalized total p-MLC (%)
c
Normalized cortical p-MLC (%)
d
Normalized cytoplasmic p-MLC (%)
e
Normalized FTE (%)
Empty
LMNA-OE
FSS for FTE measurement (dyne/cm2)
f
Control
si-LMNA
g
0 dyne/cm2
1 dyne/cm2
Control
si-LMNA
h
i
j
0 dyne/cm2+Control
1 dyne/cm2+Control
1 dyne/cm2+si-LMNA
ns
***
**
*

Fig S20

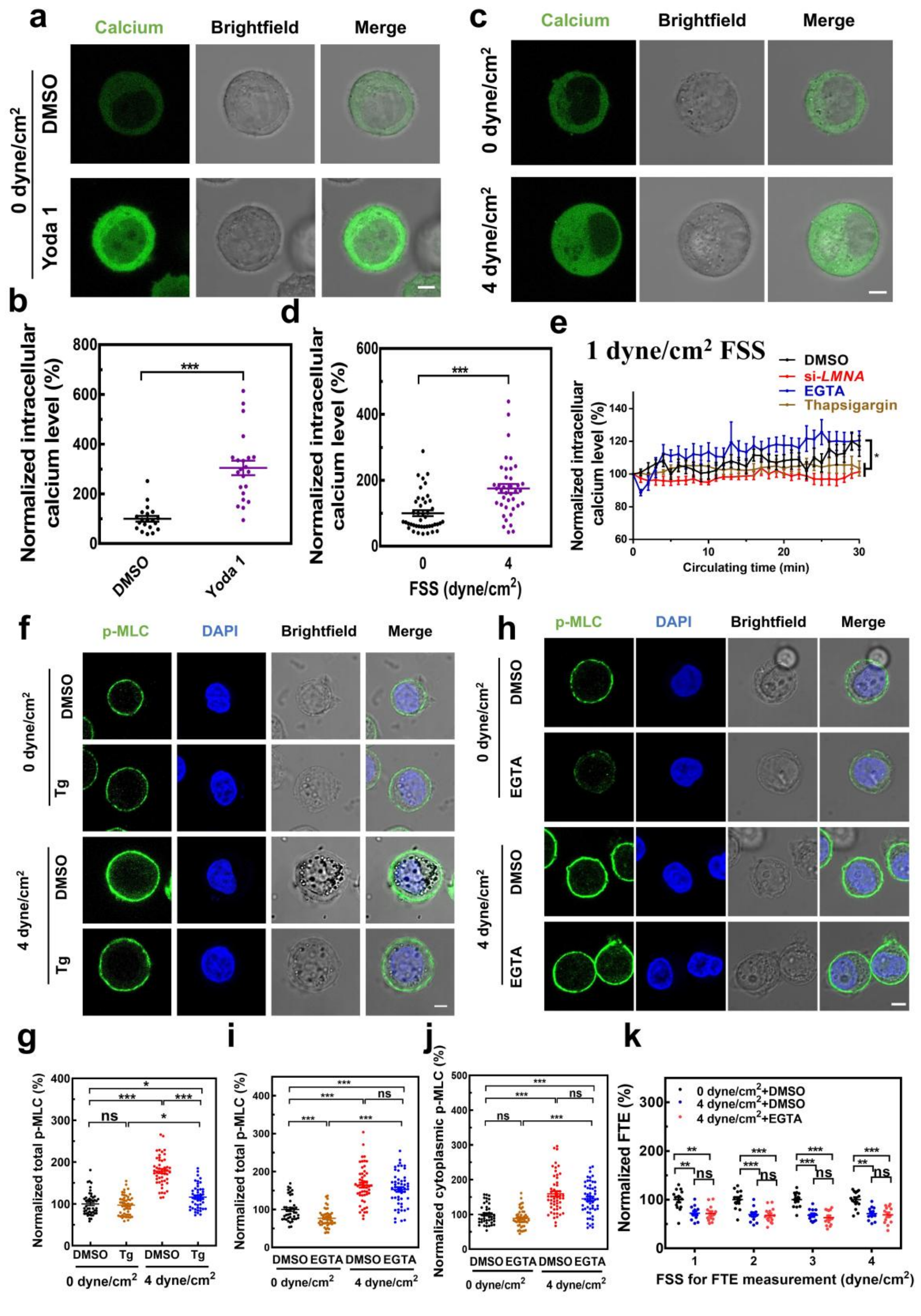

a
Calcium
Brightfield
Merge
0 dyne/cm2
DMSO
Yoda 1
b
Normalized intracellular calcium level (%)
DMSO
Yoda 1
c
0 dyne/cm2
4 dyne/cm2
d
FSS (dyne/cm2)
e
1 dyne/cm2 FSS
DMSO
si-LMNA
EGTA
Thapsigargin
Circulating time (min)
f
p-MLC
DAPI
Tg
4 dyne/cm2
h
EGTA
g
Normalized total p-MLC (%)
ns
i
j
Normalized cytoplasmic p-MLC (%)
k
Normalized FTE (%)
0 dyne/cm2+DMSO
4 dyne/cm2+DMSO
4 dyne/cm2+EGTA
FSS for FTE measurement (dyne/cm2)

Fig S21

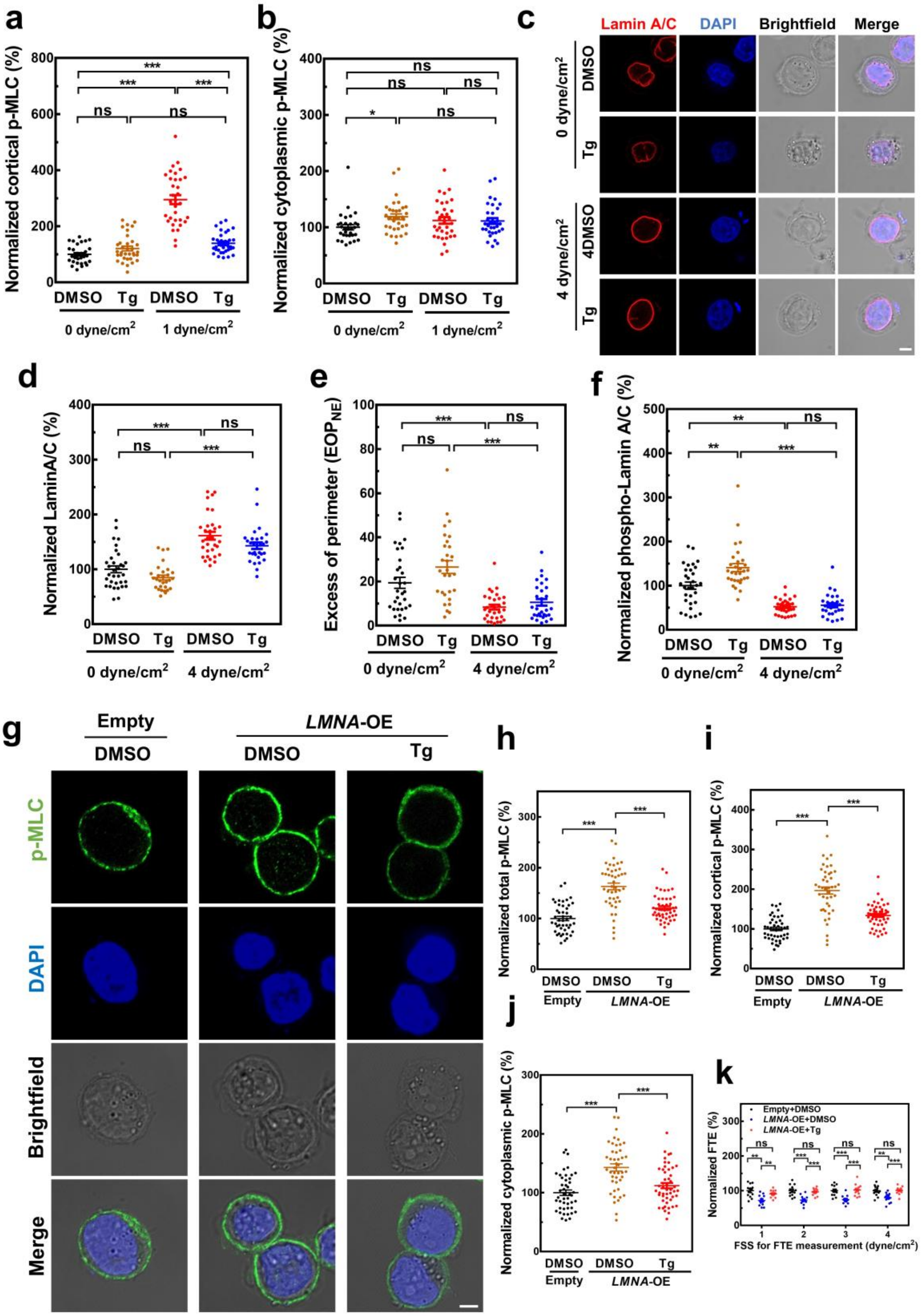

a
Normalized cortical p-MLC (%)
DMSO Tg DMSO Tg
0 dyne/cm2 1 dyne/cm2
b
Normalized cytoplasmic p-MLC (%)
DMSO Tg DMSO Tg
0 dyne/cm2 1 dyne/cm2
c
Lamin A/C DAPI Brightfield Merge
0 dyne/cm2 DMSO Tg
4 dyne/cm2 4DMSO Tg
d
Normalized LaminA/C (%)
DMSO Tg DMSO Tg
0 dyne/cm2 4 dyne/cm2
e
Excess of perimeter (EOPNE)
DMSO Tg DMSO Tg
0 dyne/cm2 4 dyne/cm2
f
Normalized phospho-Lamin A/C (%)
DMSO Tg DMSO Tg
0 dyne/cm2 4 dyne/cm2
g
Empty LMNA-OE
DMSO DMSO Tg
p-MLC
DAPI
Brightfield
Merge
h
Normalized total p-MLC (%)
DMSO DMSO Tg
Empty LMNA-OE
i
Normalized cortical p-MLC (%)
DMSO DMSO Tg
Empty LMNA-OE
j
Normalized cytoplasmic p-MLC (%)
DMSO DMSO Tg
Empty LMNA-OE
k
Empty+DMSO
LMNA-OE+DMSO
LMNA-OE+Tg
Normalized FTE (%)
FSS for FTE measurement (dyne/cm2)

Fig S22

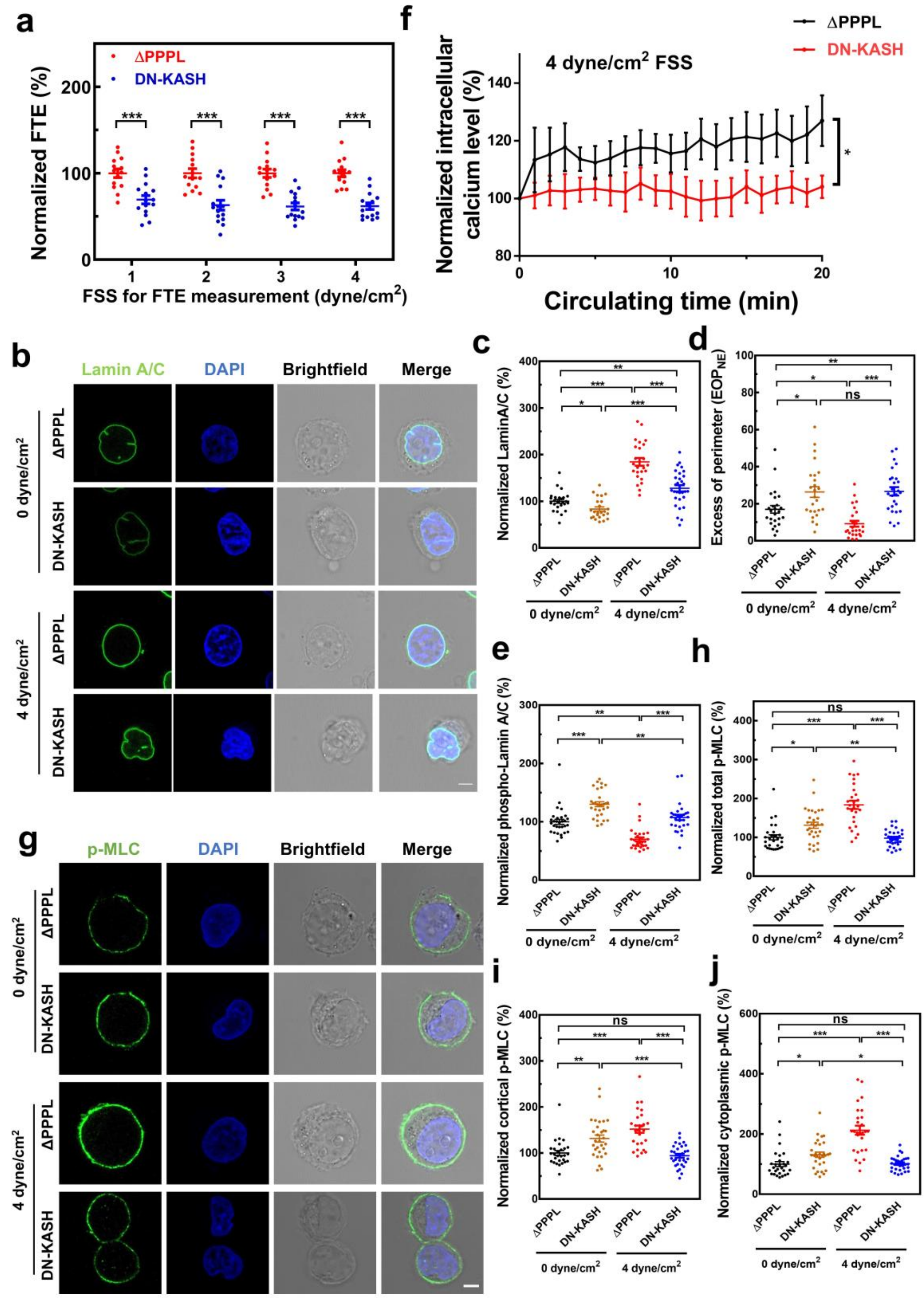
a
ΔPPPL
DN-KASH
Normalized FTE (%)
FSS for FTE measurement (dyne/cm2)
f
4 dyne/cm2 FSS
Normalized intracellular calcium level (%)
Circulating time (min)
b
Lamin A/C
DAPI
Brightfield
Merge
0 dyne/cm2
4 dyne/cm2
c
Normalized LaminA/C (%)
d
Excess of perimeter (EOPNE)
e
Normalized phospho-Lamin A/C (%)
h
Normalized total p-MLC (%)
g
p-MLC
i
Normalized cortical p-MLC (%)
j
Normalized cytoplasmic p-MLC (%)
ns

Fig S23

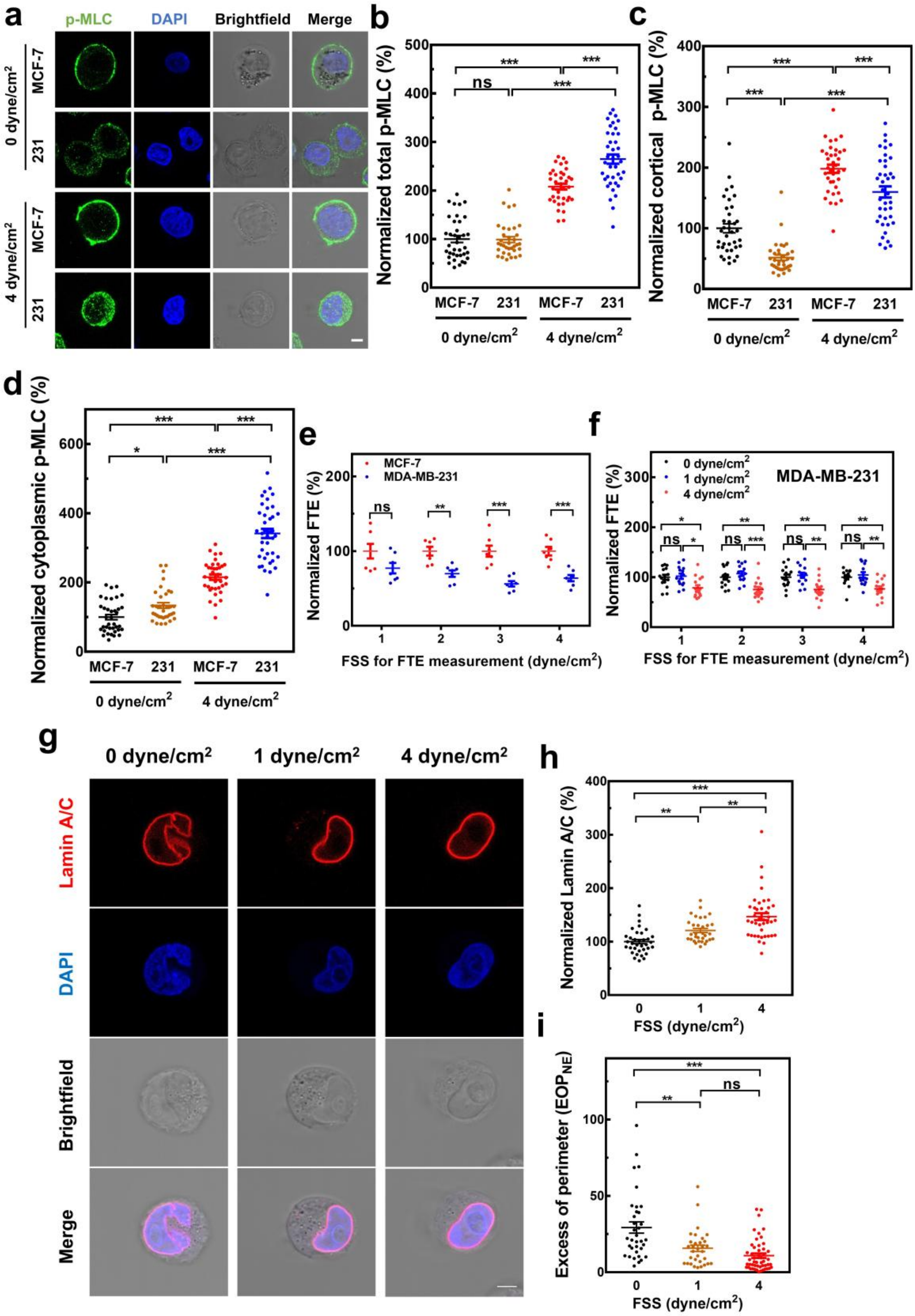

a
p-MLC
DAPI
Brightfield
Merge
0 dyne/cm2
4 dyne/cm2
MCF-7
231
b
Normalized total p-MLC (%)
ns
***
MCF-7
231
0 dyne/cm2
4 dyne/cm2
c
Normalized cortical p-MLC (%)
d
Normalized cytoplasmic p-MLC (%)
e
MCF-7
MDA-MB-231
Normalized FTE (%)
FSS for FTE measurement (dyne/cm2)
f
0 dyne/cm2
1 dyne/cm2
4 dyne/cm2
MDA-MB-231
g
0 dyne/cm2
1 dyne/cm2
4 dyne/cm2
Lamin A/C
DAPI
Brightfield
Merge
h
Normalized Lamin A/C (%)
FSS (dyne/cm2)
i
Excess of perimeter (EOPNE)

Fig S24

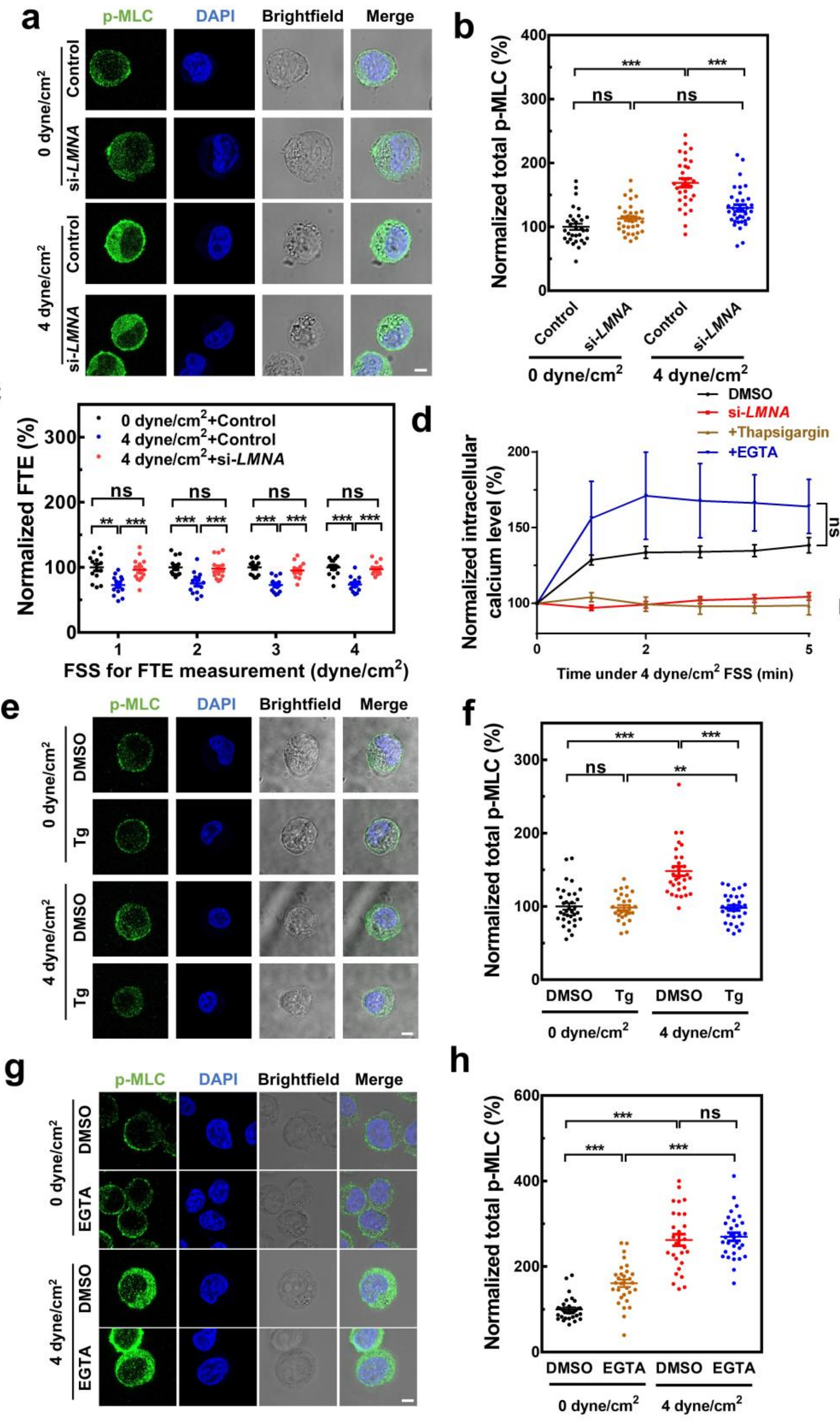
a
p-MLC
DAPI
Brightfield
Merge
0 dyne/cm2
Control
si-LMNA
4 dyne/cm2
Control
si-LMNA
b
Normalized total p-MLC (%)
***
***
ns
ns
Control
si-LMNA
Control
si-LMNA
0 dyne/cm2
4 dyne/cm2
c
Normalized FTE (%)
0 dyne/cm2+Control
4 dyne/cm2+Control
4 dyne/cm2+si-LMNA
FSS for FTE measurement (dyne/cm2)
d
DMSO
si-LMNA
+Thapsigargin
+EGTA
Normalized intracellular calcium level (%)
Time under 4 dyne/cm2 FSS (min)
e
p-MLC
DAPI
Brightfield
Merge
0 dyne/cm2
DMSO
Tg
4 dyne/cm2
DMSO
Tg
f
Normalized total p-MLC (%)
DMSO
Tg
DMSO
Tg
0 dyne/cm2
4 dyne/cm2
g
p-MLC
DAPI
Brightfield
Merge
0 dyne/cm2
DMSO
EGTA
4 dyne/cm2
DMSO
EGTA
h
Normalized total p-MLC (%)
DMSO
EGTA
DMSO
EGTA
0 dyne/cm2
4 dyne/cm2

Fig S25

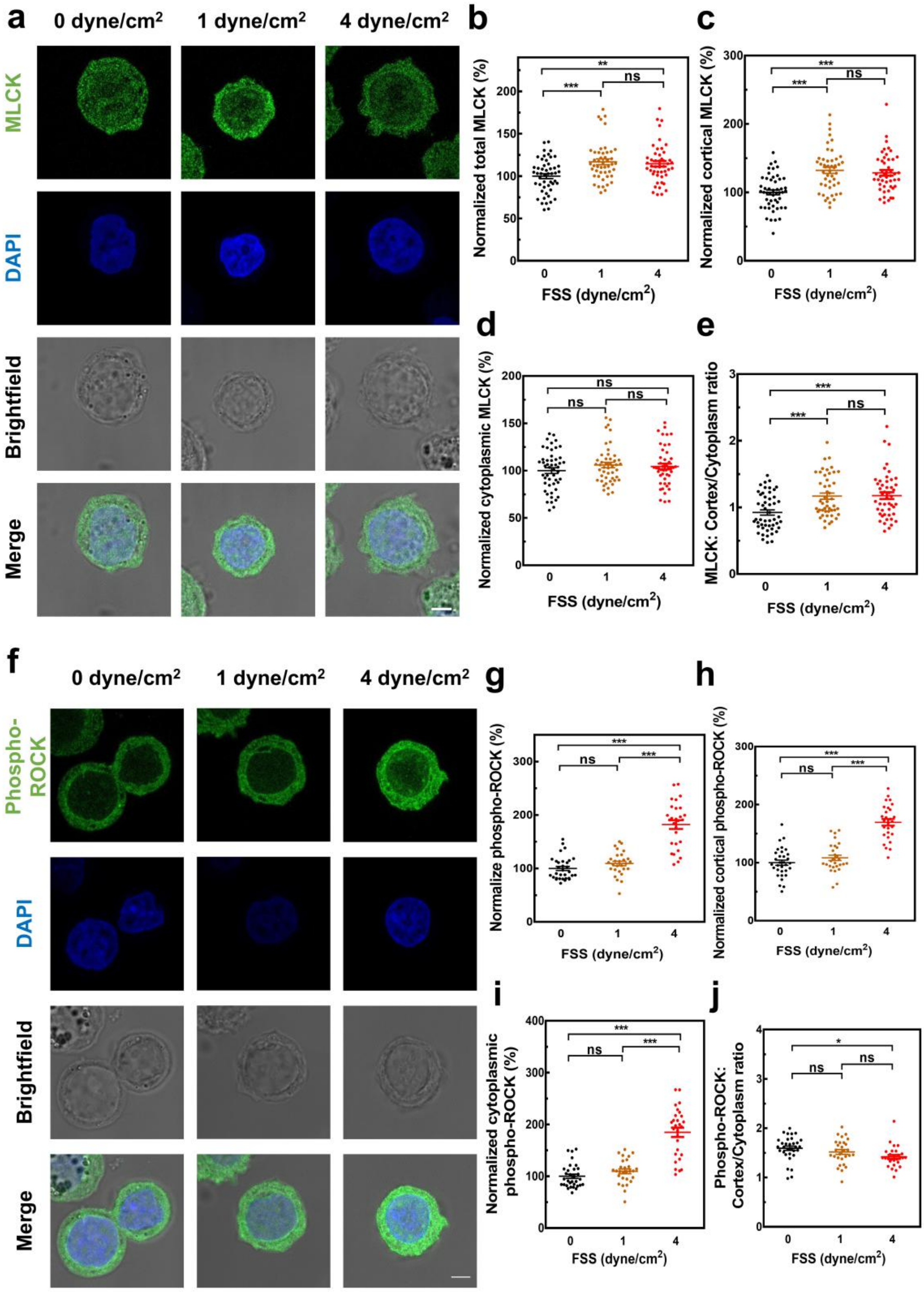

a
0 dyne/cm2
1 dyne/cm2
4 dyne/cm2
MLCK
DAPI
Brightfield
Merge
b
Normalized total MLCK (%)
FSS (dyne/cm2)
c
Normalized cortical MLCK (%)
FSS (dyne/cm2)
d
Normalized cytoplasmic MLCK (%)
FSS (dyne/cm2)
e
MLCK: Cortex/Cytoplasm ratio
FSS (dyne/cm2)
f
0 dyne/cm2
1 dyne/cm2
4 dyne/cm2
Phospho-ROCK
DAPI
Brightfield
Merge
g
Normalize phospho-ROCK (%)
FSS (dyne/cm2)
h
Normalized cortical phospho-ROCK (%)
FSS (dyne/cm2)
i
Normalized cytoplasmic phospho-ROCK (%)
FSS (dyne/cm2)
j
Phospho-ROCK: Cortex/Cytoplasm ratio
FSS (dyne/cm2)

Fig S26

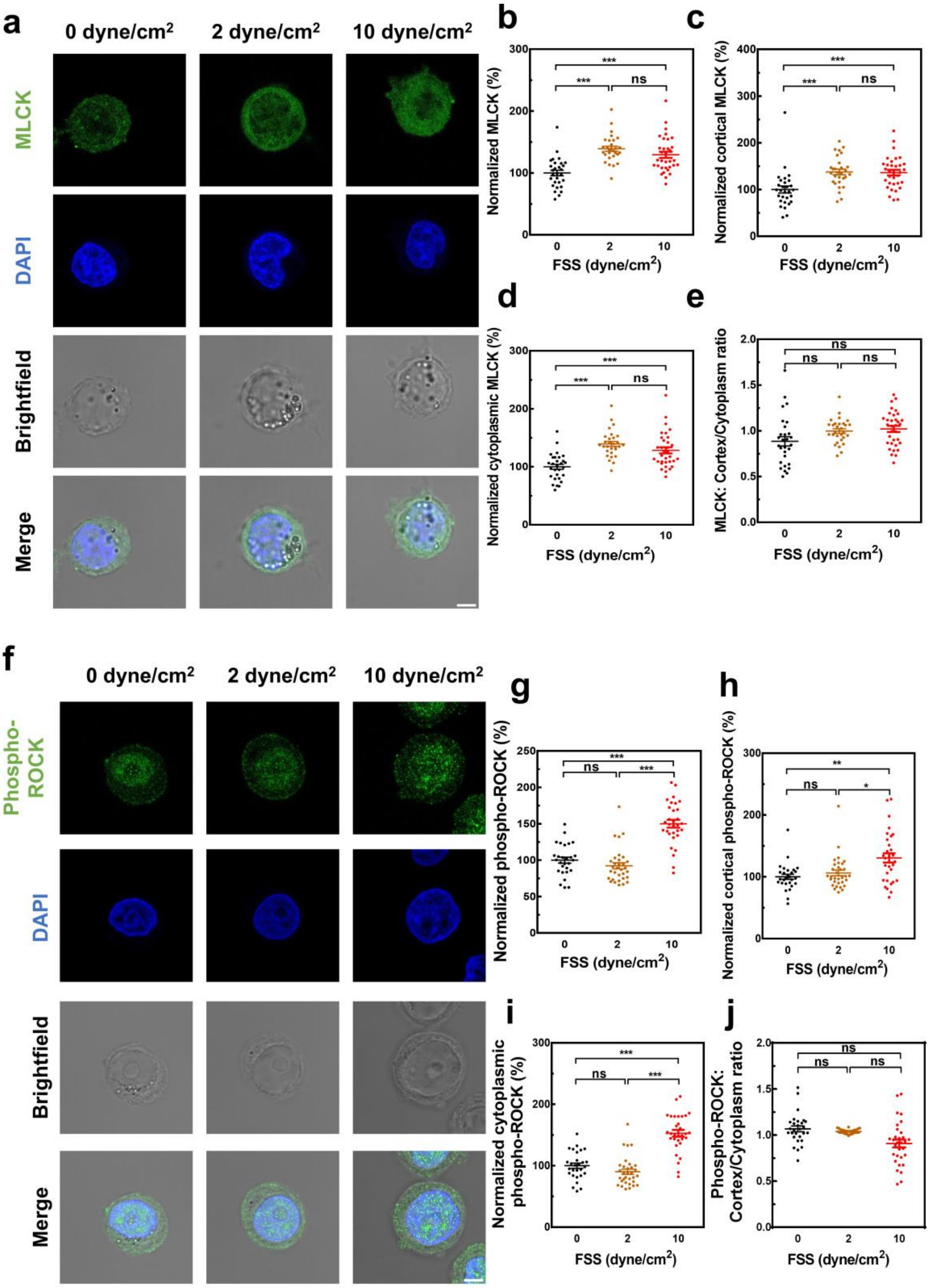
a
0 dyne/cm2
2 dyne/cm2
10 dyne/cm2
MLCK
DAPI
Brightfield
Merge
b
Normalized MLCK (%)
FSS (dyne/cm2)
c
Normalized cortical MLCK (%)
FSS (dyne/cm2)
d
Normalized cytoplasmic MLCK (%)
FSS (dyne/cm2)
e
MLCK: Cortex/Cytoplasm ratio
FSS (dyne/cm2)
f
0 dyne/cm2
2 dyne/cm2
10 dyne/cm2
Phospho-ROCK
DAPI
Brightfield
Merge
g
Normalized phospho-ROCK (%)
FSS (dyne/cm2)
h
Normalized cortical phospho-ROCK (%)
FSS (dyne/cm2)
i
Normalized cytoplasmic phospho-ROCK (%)
FSS (dyne/cm2)
j
Phospho-ROCK: Cortex/Cytoplasm ratio
FSS (dyne/cm2)

Fig S27

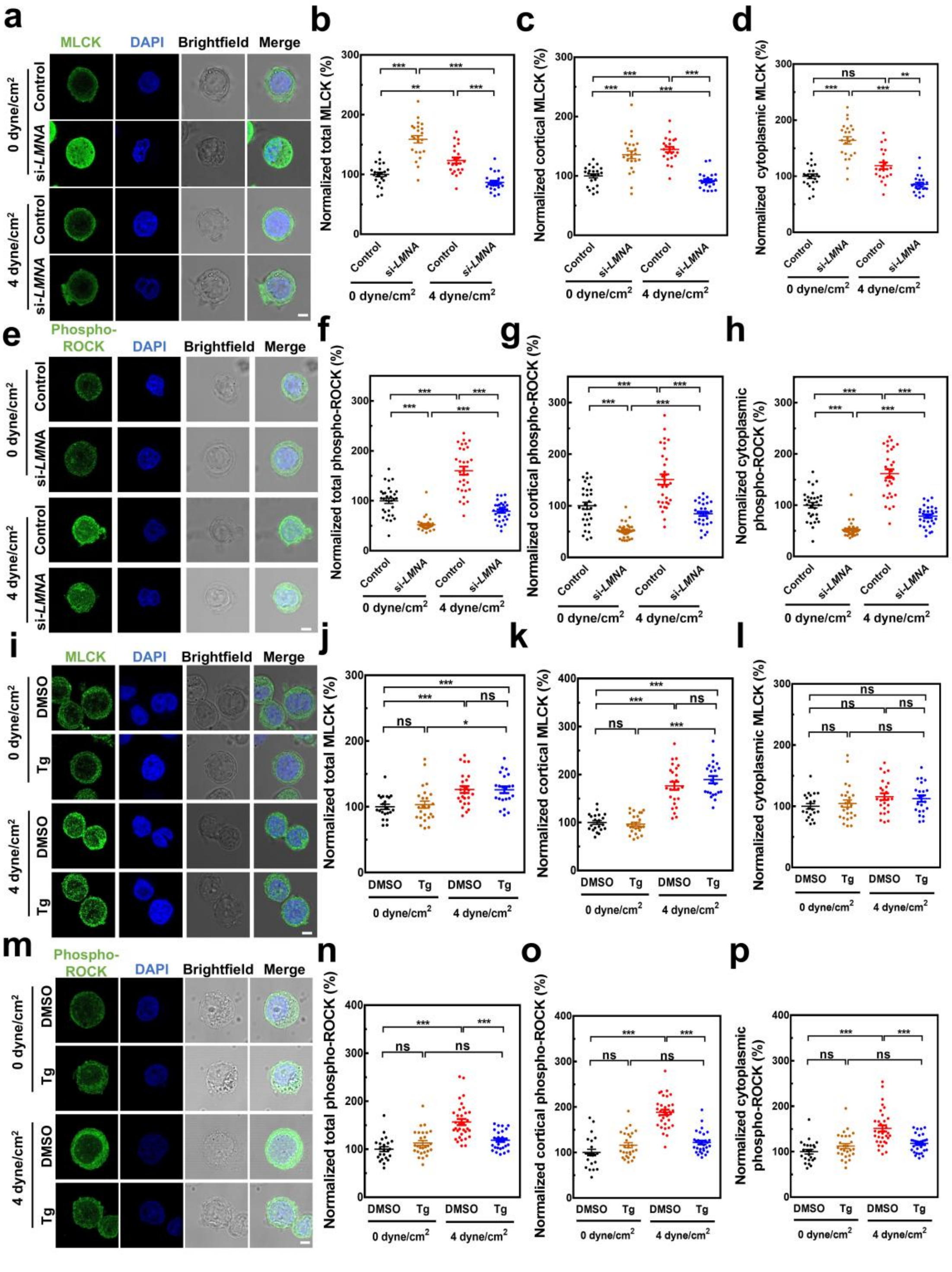

Fig S28

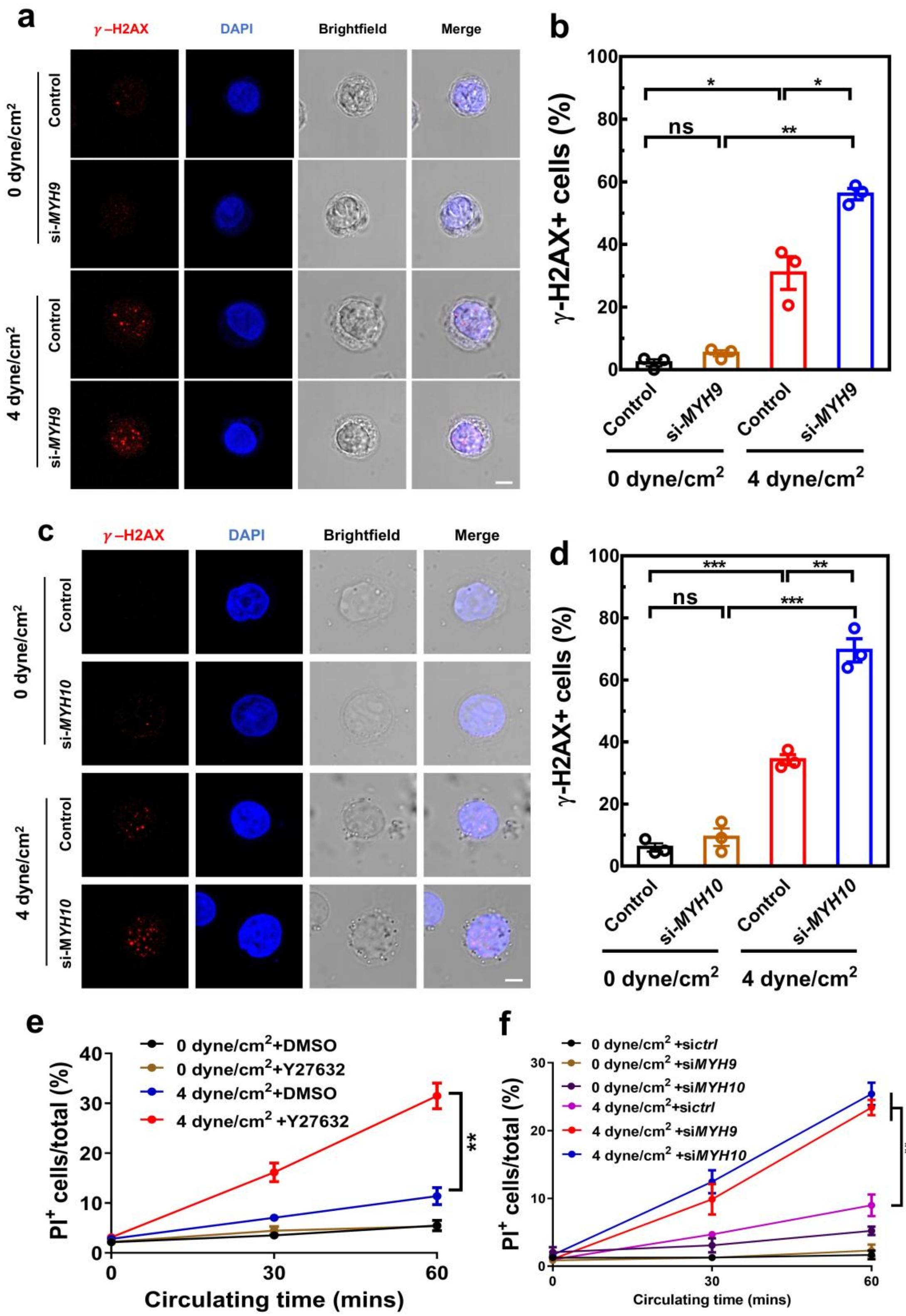
a
γ –H2AX
DAPI
Brightfield
Merge
0 dyne/cm²
Control
si-MYH9
4 dyne/cm²
Control
si-MYH9
b
γ-H2AX+ cells (%)
ns
*
**
Control
si-MYH9
0 dyne/cm²
4 dyne/cm²
c
γ –H2AX
DAPI
Brightfield
Merge
0 dyne/cm²
Control
si-MYH10
4 dyne/cm²
Control
si-MYH10
d
γ-H2AX+ cells (%)
ns
***
**
Control
si-MYH10
0 dyne/cm²
4 dyne/cm²
e
0 dyne/cm²+DMSO
0 dyne/cm²+Y27632
4 dyne/cm²+DMSO
4 dyne/cm² +Y27632
PI⁺ cells/total (%)
Circulating time (mins)
**
f
0 dyne/cm² +sictrl
0 dyne/cm² +siMYH9
0 dyne/cm² +siMYH10
4 dyne/cm²+sictrl
4 dyne/cm² +siMYH9
4 dyne/cm² +siMYH10
PI⁺ cells/total (%)
Circulating time (mins)
**

Fig S29

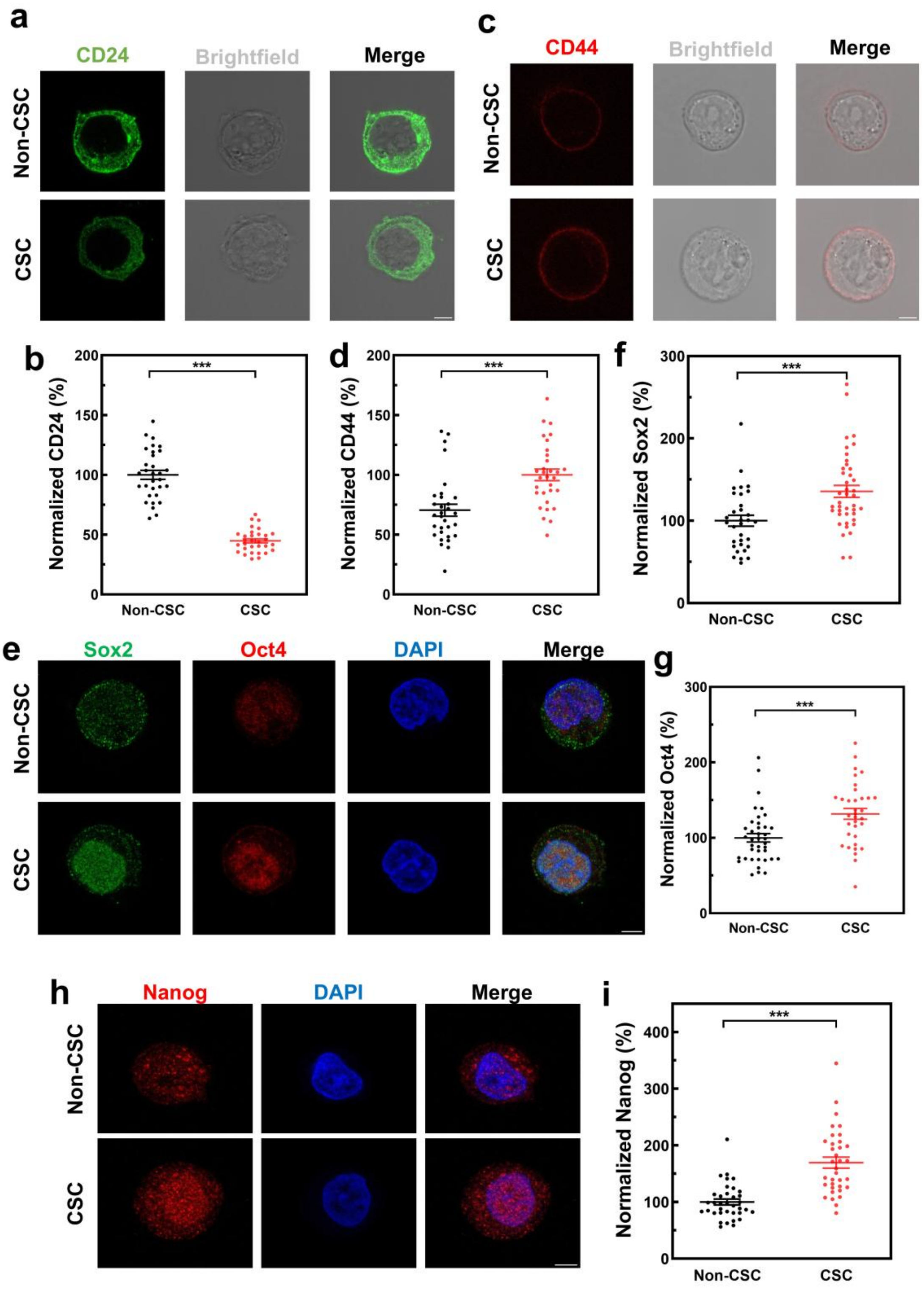
a
CD24
Brightfield
Merge
Non-CSC
CSC
c
CD44
Brightfield
Merge
Non-CSC
CSC
b
Normalized CD24 (%)
***
Non-CSC
CSC
d
Normalized CD44 (%)
***
Non-CSC
CSC
f
Normalized Sox2 (%)
***
Non-CSC
CSC
e
Sox2
Oct4
DAPI
Merge
Non-CSC
CSC
g
Normalized Oct4 (%)
***
Non-CSC
CSC
h
Nanog
DAPI
Merge
Non-CSC
CSC
i
Normalized Nanog (%)
***
Non-CSC
CSC

Fig S30

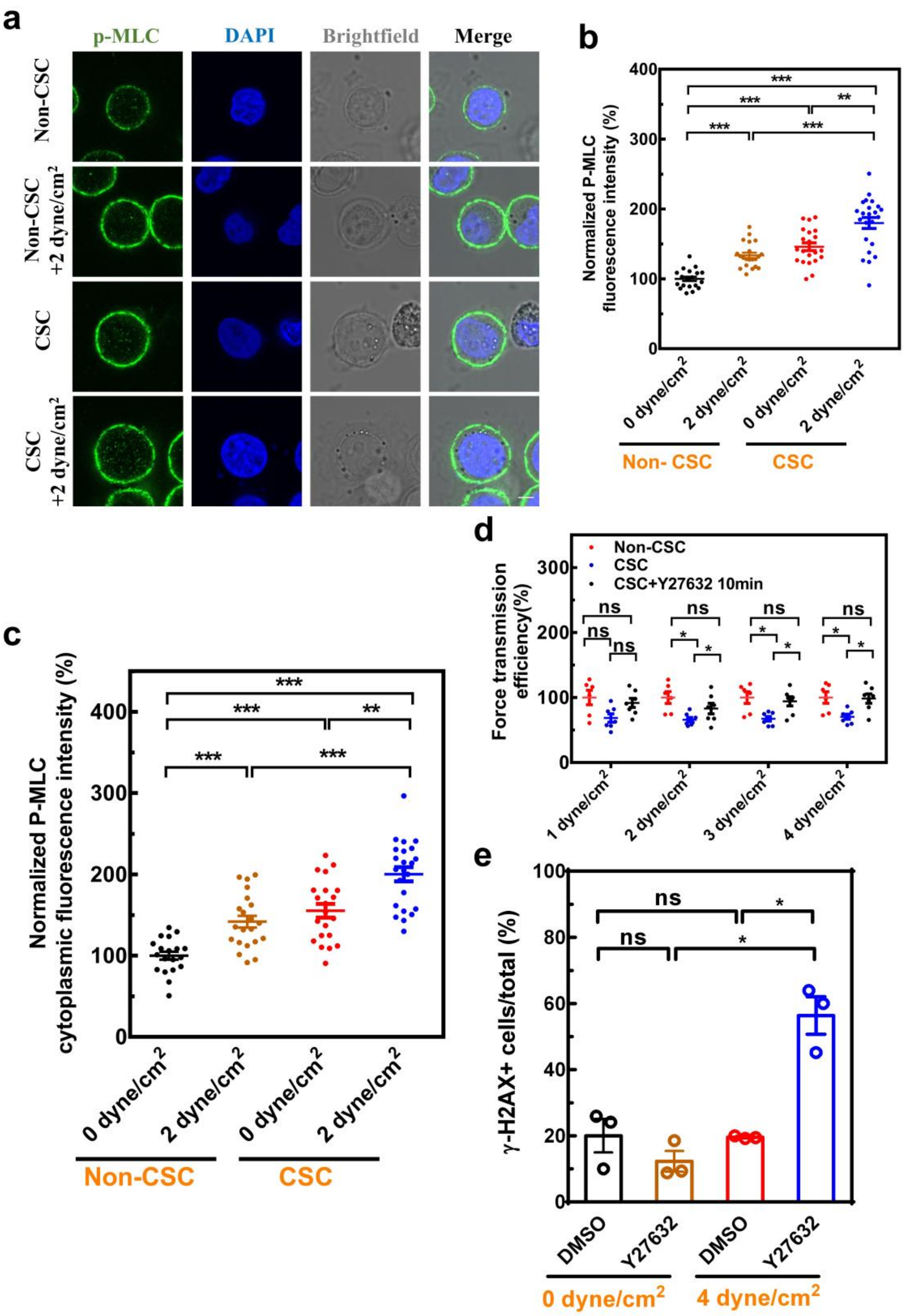
a
p-MLC
DAPI
Brightfield
Merge
Non-CSC
Non-CSC +2 dyne/cm²
CSC
CSC +2 dyne/cm²
b
Normalized P-MLC fluorescence intensity (%)
0 dyne/cm²
2 dyne/cm²
0 dyne/cm²
2 dyne/cm²
Non- CSC
CSC
c
Normalized P-MLC cytoplasmic fluorescence intensity (%)
0 dyne/cm²
2 dyne/cm²
0 dyne/cm²
2 dyne/cm²
Non-CSC
CSC
d
Non-CSC
CSC
CSC+Y27632 10min
Force transmission efficiency(%)
ns
1 dyne/cm²
2 dyne/cm²
3 dyne/cm²
4 dyne/cm²
e
γ-H2AX+ cells/total (%)
ns
DMSO
Y27632
DMSO
Y27632
0 dyne/cm²
4 dyne/cm²

Fig S31

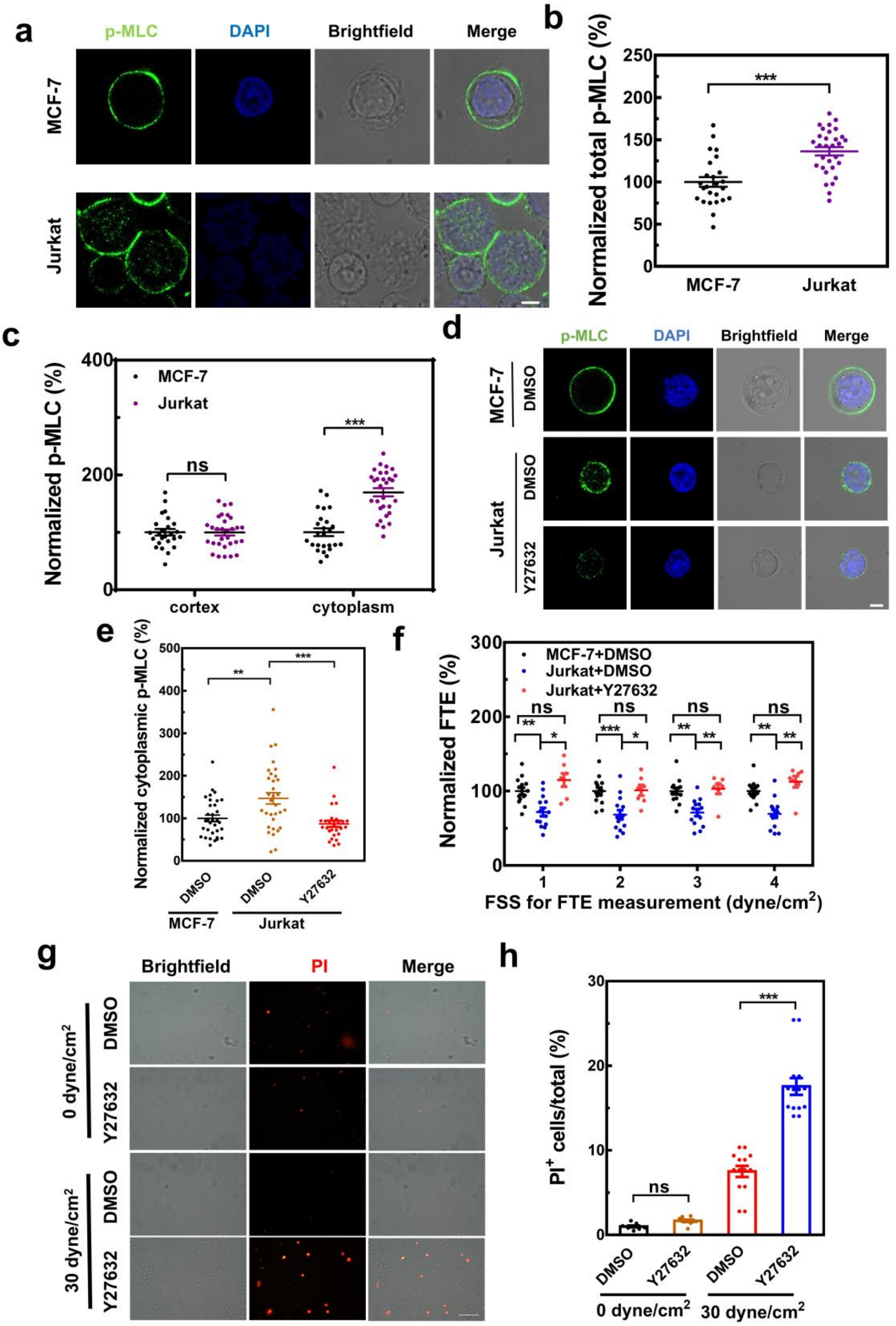

a
p-MLC
DAPI
Brightfield
Merge
MCF-7
Jurkat
b
Normalized total p-MLC (%)
***
MCF-7
Jurkat
c
Normalized p-MLC (%)
MCF-7
Jurkat
ns
***
cortex
cytoplasm
d
p-MLC
DAPI
Brightfield
Merge
MCF-7
DMSO
Jurkat
DMSO
Y27632
e
Normalized cytoplasmic p-MLC (%)
**
***
DMSO
DMSO
Y27632
MCF-7
Jurkat
f
Normalized FTE (%)
MCF-7+DMSO
Jurkat+DMSO
Jurkat+Y27632
FSS for FTE measurement (dyne/cm2)
g
Brightfield
PI
Merge
0 dyne/cm2
30 dyne/cm2
DMSO
Y27632
h
PI+ cells/total (%)
ns
***
DMSO
Y27632
0 dyne/cm2
30 dyne/cm2

Fig S32

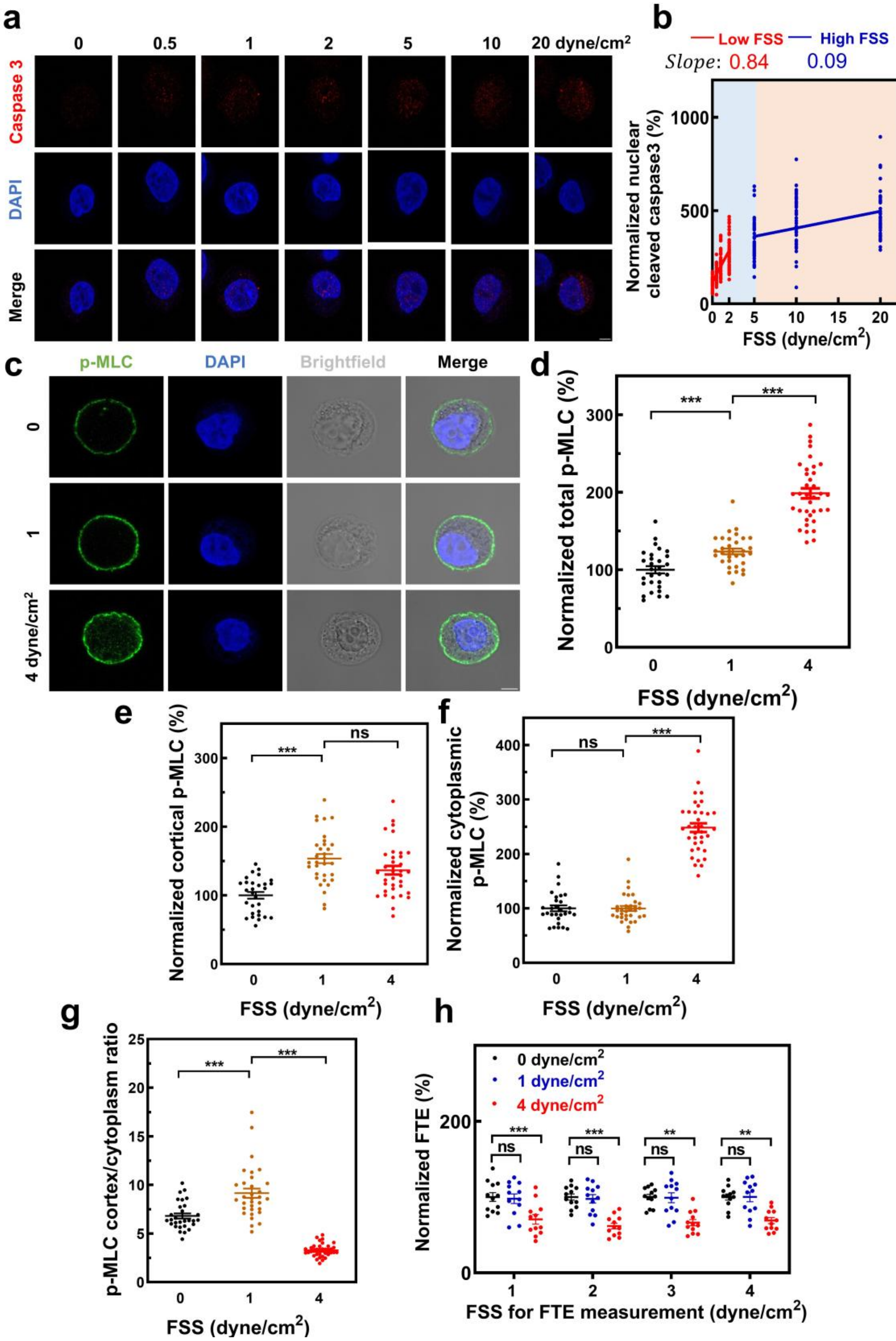

a
0
0.5
1
2
5
10
20 dyne/cm²
Caspase 3
DAPI
Merge
b
Low FSS
High FSS
Slope: 0.84 0.09
Normalized nuclear cleaved caspase3 (%)
FSS (dyne/cm²)
c
p-MLC
DAPI
Brightfield
Merge
0
1
4 dyne/cm²
d
Normalized total p-MLC (%)
FSS (dyne/cm²)
e
Normalized cortical p-MLC (%)
FSS (dyne/cm²)
f
Normalized cytoplasmic p-MLC (%)
FSS (dyne/cm²)
g
p-MLC cortex/cytoplasm ratio
FSS (dyne/cm²)
h
0 dyne/cm²
1 dyne/cm²
4 dyne/cm²
Normalized FTE (%)
FSS for FTE measurement (dyne/cm²)

Fig S33

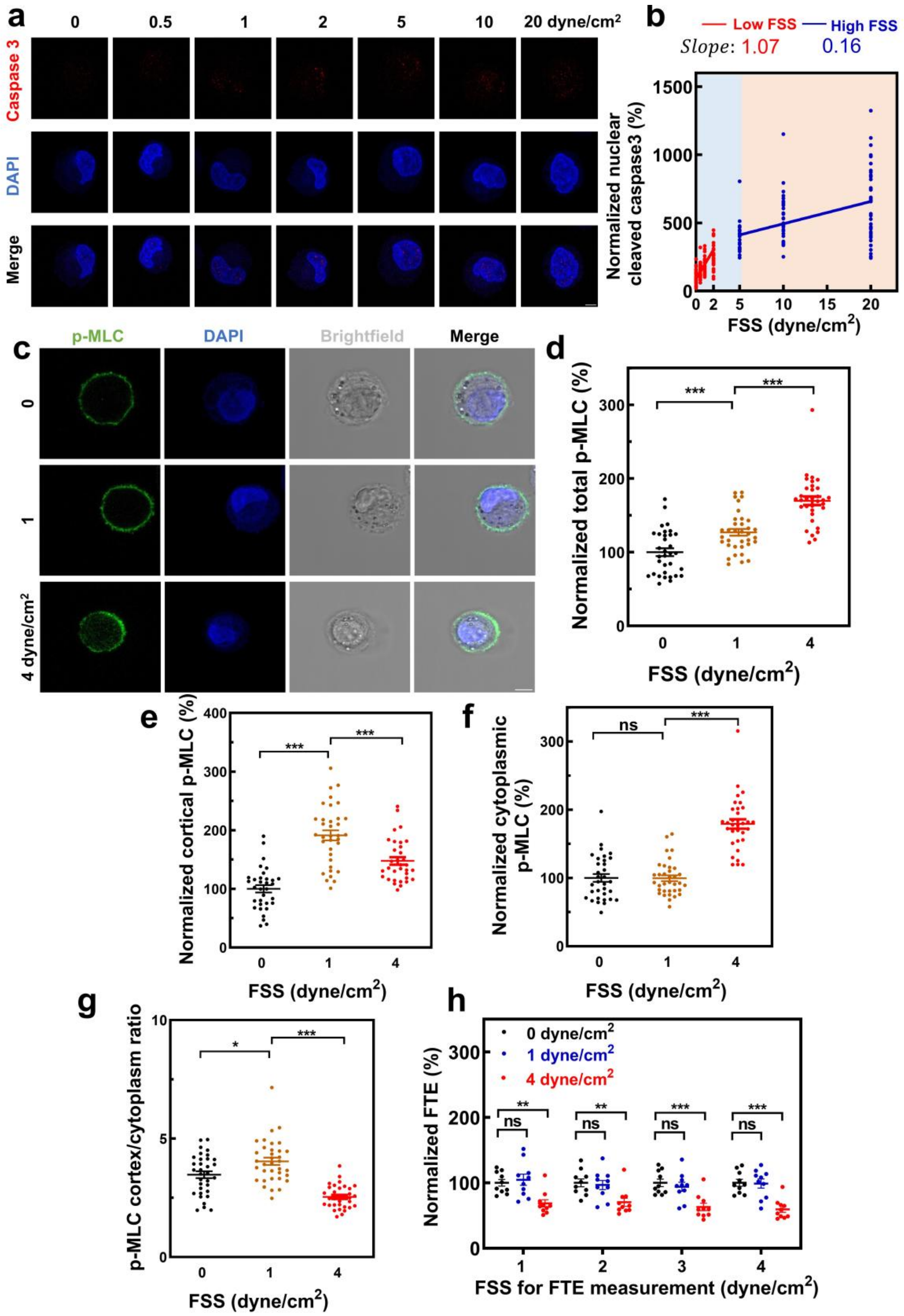
a
0
0.5
1
2
5
10
20 dyne/cm2
Caspase 3
DAPI
Merge
b
Low FSS
High FSS
Slope: 1.07
0.16
Normalized nuclear cleaved caspase3 (%)
FSS (dyne/cm2)
c
p-MLC
DAPI
Brightfield
Merge
0
1
4 dyne/cm2
d
Normalized total p-MLC (%)
FSS (dyne/cm2)
e
Normalized cortical p-MLC (%)
FSS (dyne/cm2)
f
Normalized cytoplasmic p-MLC (%)
FSS (dyne/cm2)
g
p-MLC cortex/cytoplasm ratio
FSS (dyne/cm2)
h
0 dyne/cm2
1 dyne/cm2
4 dyne/cm2
Normalized FTE (%)
FSS for FTE measurement (dyne/cm2)

Fig S34

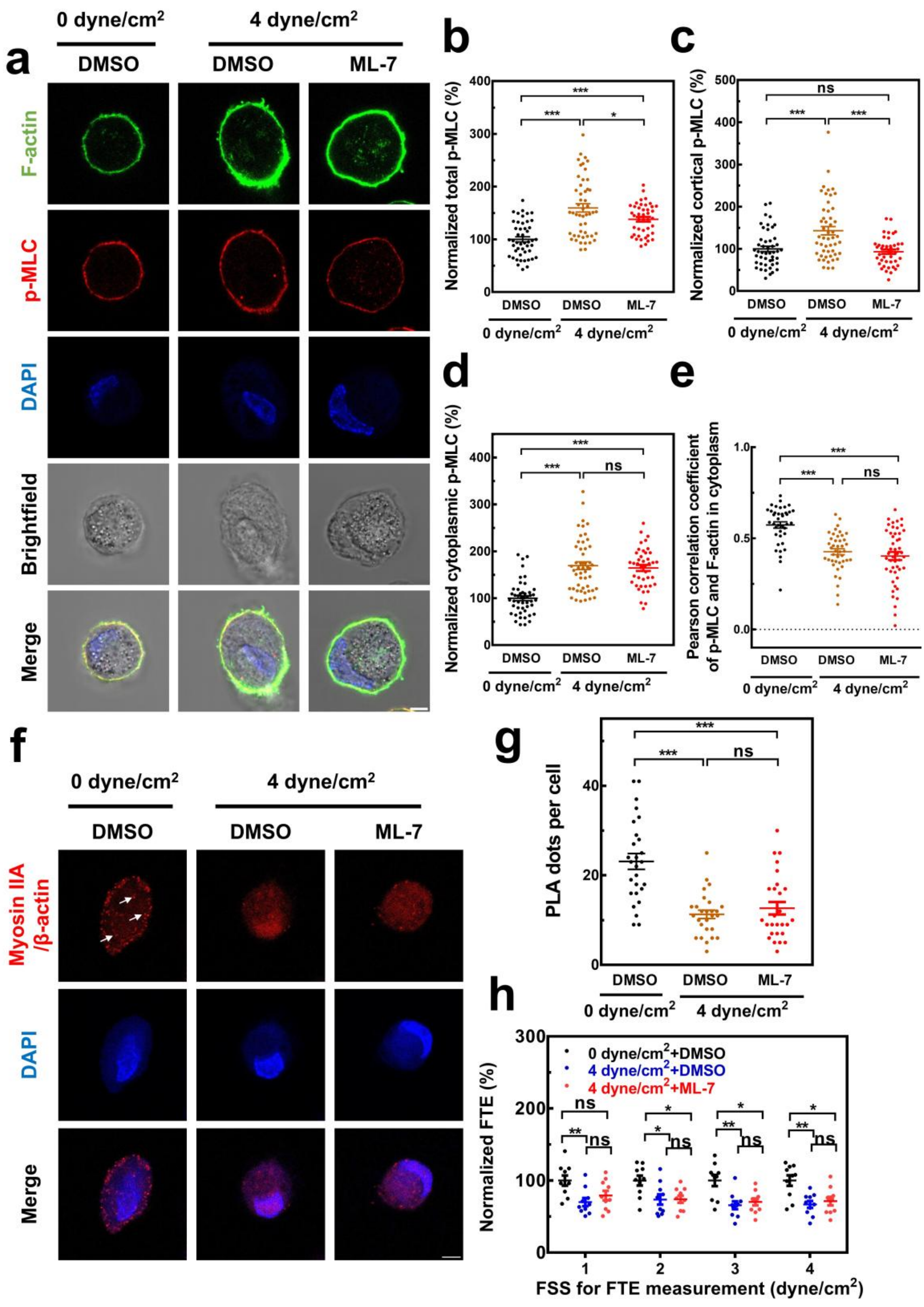

a
0 dyne/cm2
4 dyne/cm2
DMSO
DMSO
ML-7
F-actin
p-MLC
DAPI
Brightfield
Merge
b
Normalized total p-MLC (%)
c
Normalized cortical p-MLC (%)
d
Normalized cytoplasmic p-MLC (%)
e
Pearson correlation coefficient of p-MLC and F-actin in cytoplasm
f
Myosin IIA /β-actin
g
PLA dots per cell
h
0 dyne/cm2+DMSO
4 dyne/cm2+DMSO
4 dyne/cm2+ML-7
Normalized FTE (%)
FSS for FTE measurement (dyne/cm2)

Fig S35

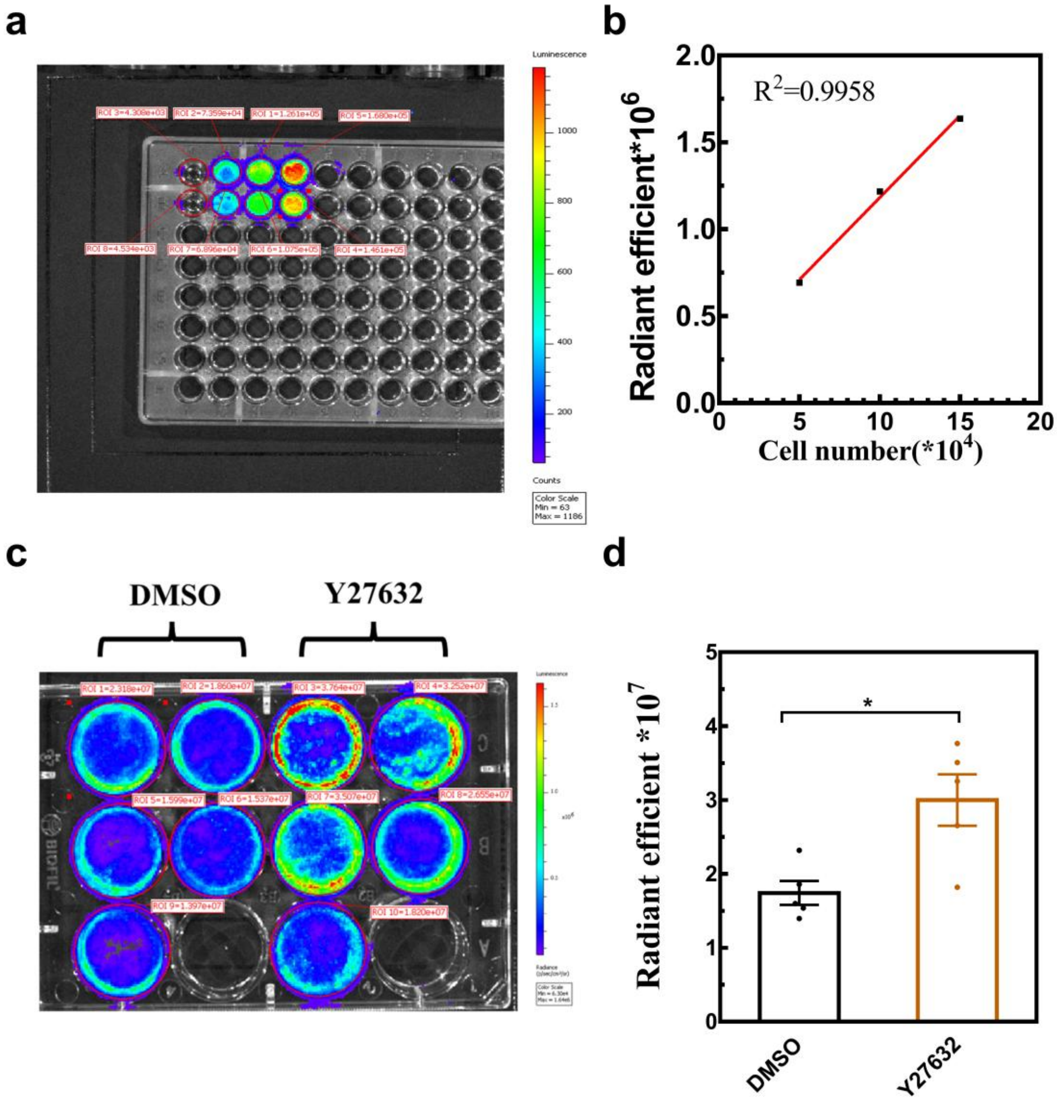

a
b
R2=0.9958
Radiant efficient*10^6
Cell number(*10^4)
c
DMSO
Y27632
d
Radiant efficient *10^7
*
DMSO
Y27632

Fig S36

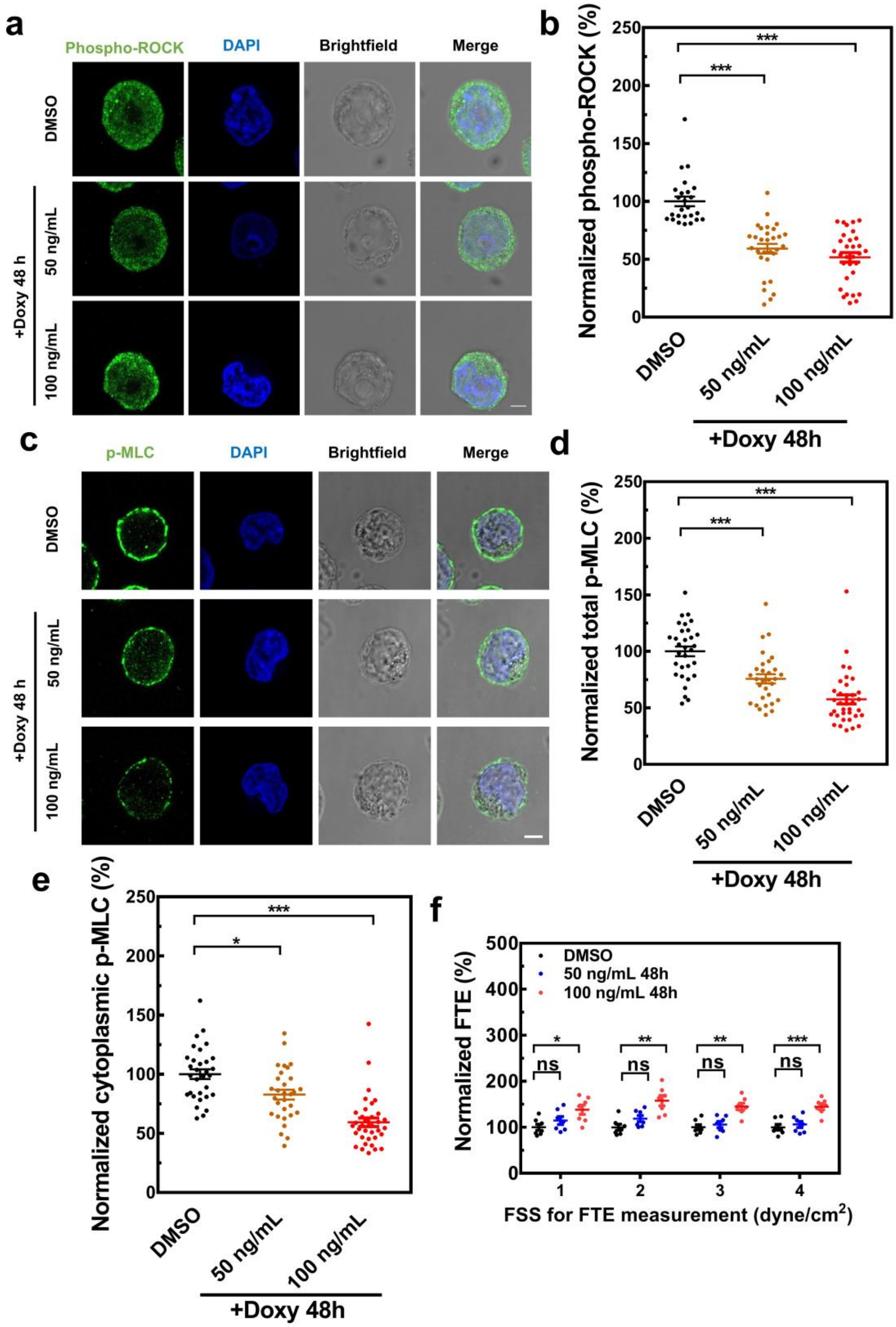
a
Phospho-ROCK
DAPI
Brightfield
Merge
DMSO
+Doxy 48 h
50 ng/mL
100 ng/mL
b
Normalized phospho-ROCK (%)
***
***
DMSO
50 ng/mL
100 ng/mL
+Doxy 48h
c
p-MLC
DAPI
Brightfield
Merge
DMSO
+Doxy 48 h
50 ng/mL
100 ng/mL
d
Normalized total p-MLC (%)
***
***
DMSO
50 ng/mL
100 ng/mL
+Doxy 48h
e
Normalized cytoplasmic p-MLC (%)
***
*
DMSO
50 ng/mL
100 ng/mL
+Doxy 48h
f
Normalized FTE (%)
DMSO
50 ng/mL 48h
100 ng/mL 48h
ns
*
**
***
FSS for FTE measurement (dyne/cm2)

Fig S37

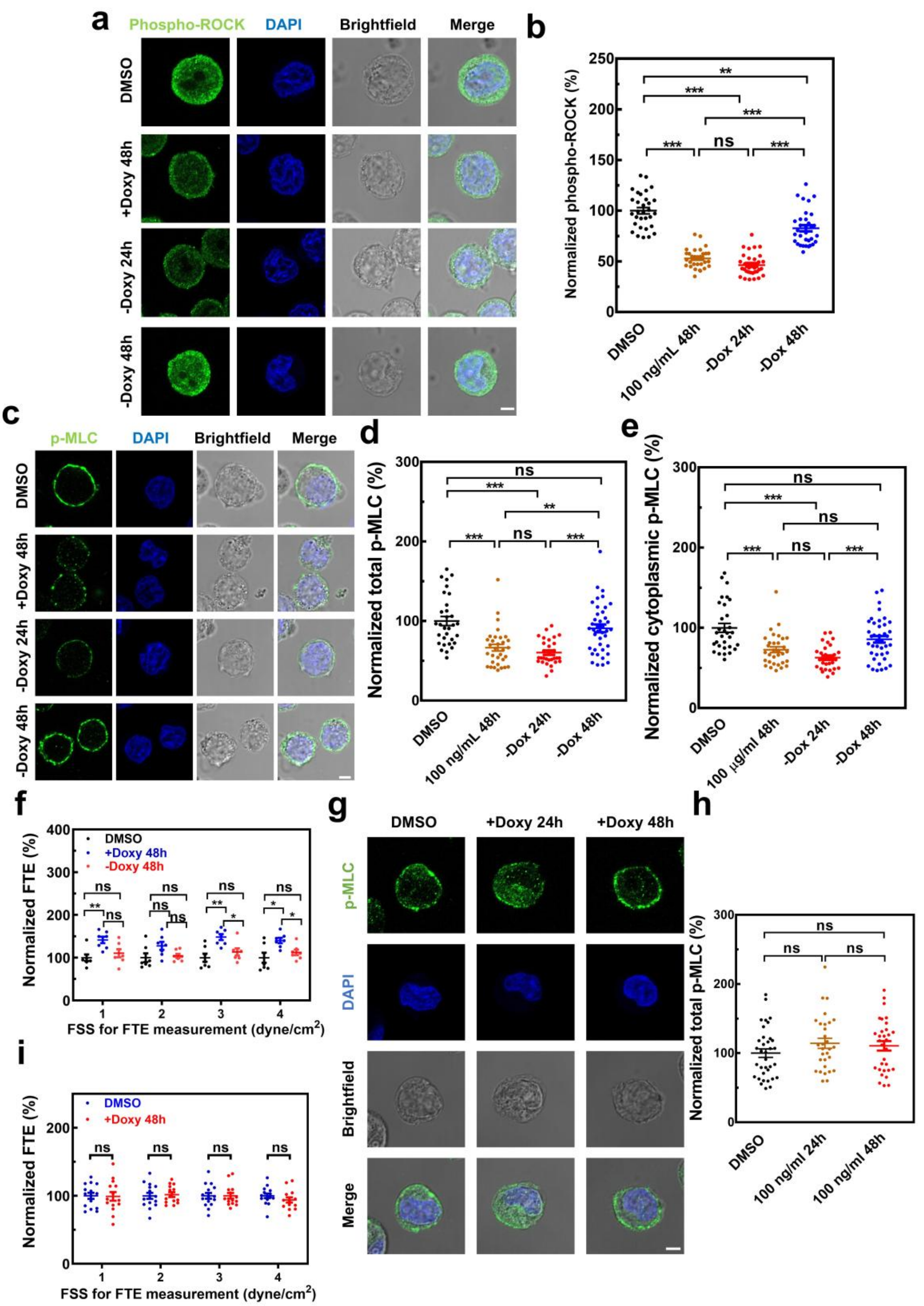
a
Phospho-ROCK
DAPI
Brightfield
Merge
DMSO
+Doxy 48h
-Doxy 24h
-Doxy 48h
b
Normalized phospho-ROCK (%)
DMSO
100 ng/mL 48h
-Dox 24h
-Dox 48h
c
p-MLC
d
Normalized total p-MLC (%)
e
Normalized cytoplasmic p-MLC (%)
100 μg/ml 48h
f
Normalized FTE (%)
+Doxy 48h
-Doxy 48h
FSS for FTE measurement (dyne/cm^2)
g
+Doxy 24h
h
100 ng/ml 24h
100 ng/ml 48h
i

Fig S38

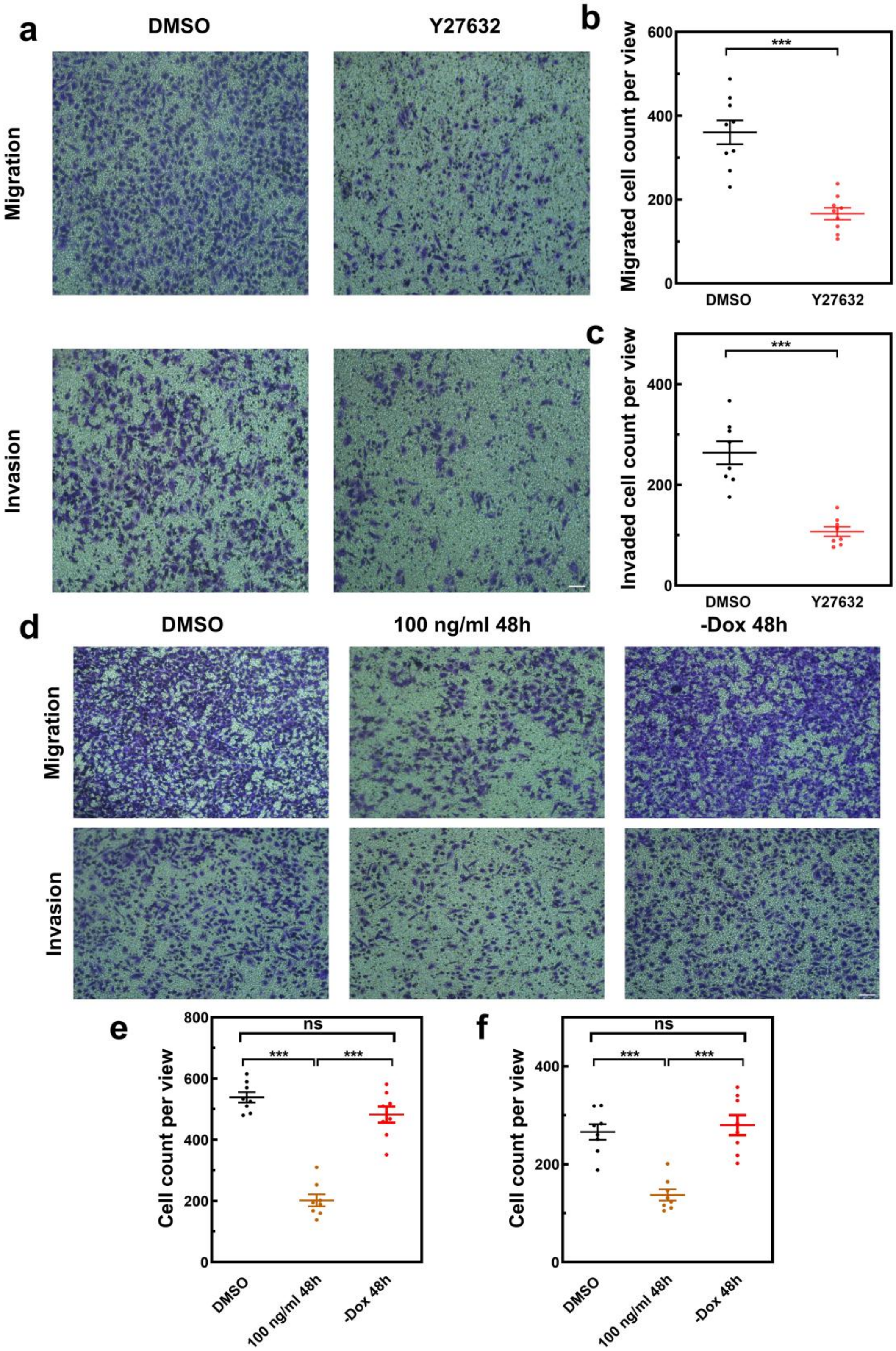
a
DMSO
Y27632
Migration
Invasion
b
Migrated cell count per view
600
400
200
0
***
DMSO
Y27632
c
Invaded cell count per view
400
200
0
***
DMSO
Y27632
d
DMSO
100 ng/ml 48h
-Dox 48h
Migration
Invasion
e
Cell count per view
800
600
400
200
0
ns
***
***
DMSO
100 ng/ml 48h
-Dox 48h
f
Cell count per view
400
200
0
ns
***
***
DMSO
100 ng/ml 48h
-Dox 48h

Fig S39

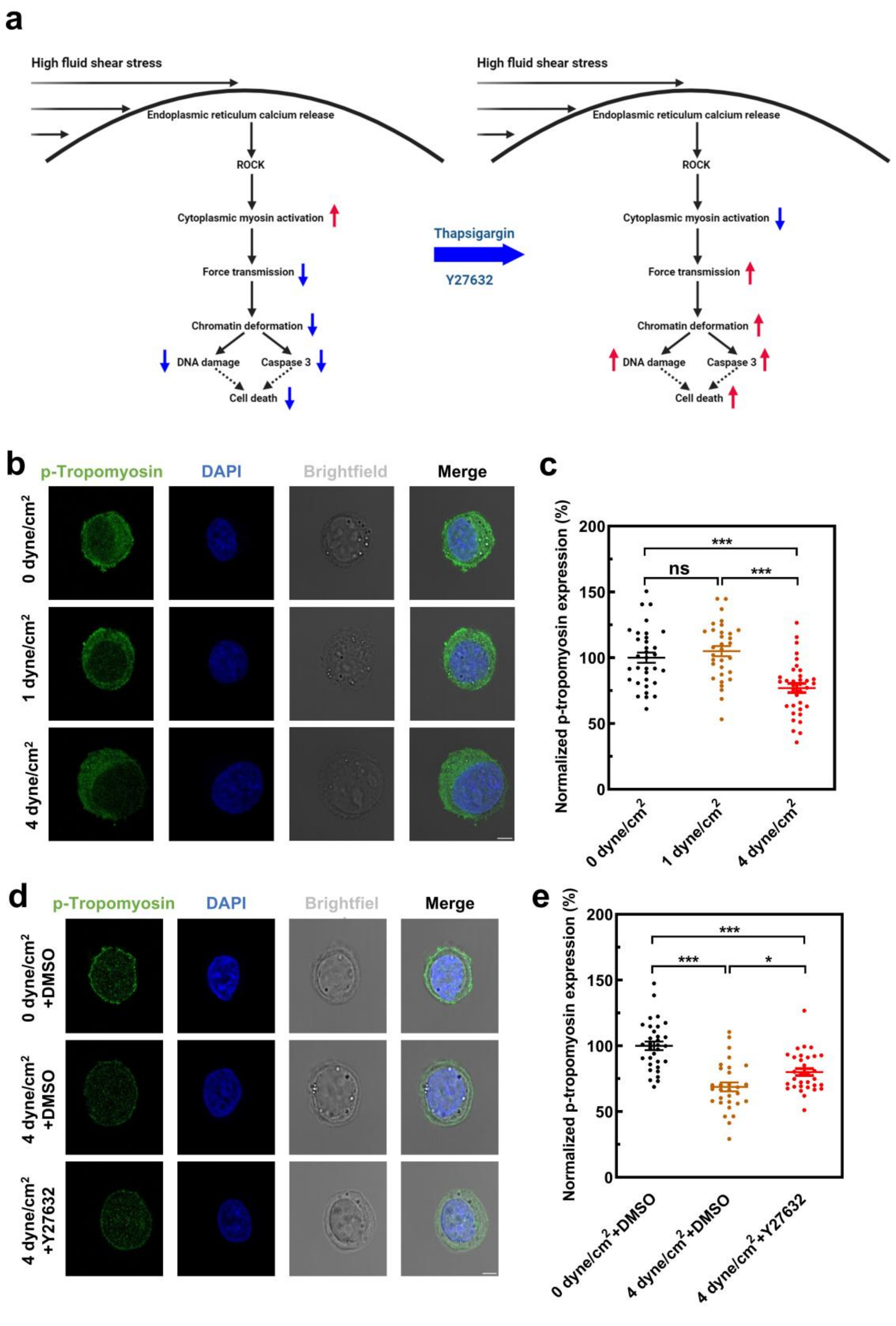

a
High fluid shear stress
Endoplasmic reticulum calcium release
ROCK
Cytoplasmic myosin activation
Force transmission
Chromatin deformation
DNA damage
Caspase 3
Cell death
Thapsigargin
Y27632
High fluid shear stress
Endoplasmic reticulum calcium release
ROCK
Cytoplasmic myosin activation
Force transmission
Chromatin deformation
DNA damage
Caspase 3
Cell death
b
p-Tropomyosin
DAPI
Brightfield
Merge
0 dyne/cm²
1 dyne/cm²
4 dyne/cm²
c
Normalized p-tropomyosin expression (%)
200
150
100
50
0
***
ns
***
0 dyne/cm²
1 dyne/cm²
4 dyne/cm²
d
p-Tropomyosin
DAPI
Brightfiel
Merge
0 dyne/cm² +DMSO
4 dyne/cm² +DMSO
4 dyne/cm² +Y27632
e
Normalized p-tropomyosin expression (%)
200
150
100
50
0
***
***
*
0 dyne/cm²+DMSO
4 dyne/cm²+DMSO
4 dyne/cm²+Y27632

Supplementary Figure Legend

Figure S1 Non-adherent tumor cells exhibit similar morphology and adhesion on poly-L-lysine (PLL) coated substrate compared with suspended status. (a) Schematic of non-adherent tumor cells attached to PLL-coated microfluidic chip. **(b)** Schematic of non-adherent tumor cells within the circulatory system. Representative immunofluorescence images **(c)** and quantification of cross section area **(d)**, shape factor **(e)** and vinculin **(f)** after the attachment to PLL-coated substrate. n=25, 20, 24, 30 and 22 cells for adherent, PLL 10 min, PLL 30 min, PLL 60 min and suspended, respectively. Scale bar, 5 μm for suspended and 10 μm for adherent. **(g)** Quantification of nuclear cleaved caspase 3 in PLL-attached cells after the treatment under 0, 0.25, 0.5, 1, 2, 4 and 8 dyne/cm^2 FSS for an hour. Non-adherent MCF-7 cells were attached to a PLL-coated microfluidic chip and treated under varying levels of FSS for one hour. The red and blue lines represent the linear regression of data within the ranges of low and high FSS. n=50, 26, 27, 24, 22, 26 and 22 cells for 0, 0.25, 0.5, 1, 2, 4 and 8 dyne/cm^2, respectively. **(h)** Quantification of γ-H2Ax in PLL-attached cells after the treatment under 0, 0.25, 0.5, 1, 2, 4 and 8 dyne/cm^2 FSS for an hour. Non-adherent MCF-7 cells were attached to a PLL-coated microfluidic chip and treated under varying levels of FSS for one hour. The red and blue lines represent the linear regression of data within the ranges of low and high FSS. n=37, 31, 31, 32, 31, 32 and 34 cells for 0, 0.25, 0.5, 1, 2, 4 and 8 dyne/cm^2, respectively. Representative immunofluorescence images **(i)** and quantification **(j)** of nuclear cleaved caspase 3 under the treatment of 1 or 4 dyne/cm^2 FSS for the indicated durations. Non-adherent tumor cells were attached to PLL-coated substrate and treated for varying levels of FSS. n=30, 30, 34, 37 and 34 cells for 0, 10, 20, 30 and 60 min under 1 dyne/cm^2 FSS; n=33, 31, 33, 31 and 31 cells for 0, 10, 20, 30 and 60 min under 4 dyne/cm^2 FSS. Scale bar, 5 μm. Data were summarized from 3 biological repeats and presented as mean ± SEM. Kruskal–Wallis one-way ANOVA followed by Mann–Whitney U-test with Bonferroni correction was adopted for comparison in **(d-f)**. ns, no significant difference; ***, $p < 0.001$.

Figure S2 Quantification of H2B displacement and force transmission efficiency under FSS. (a-b) Loss modulus **(a)** and dynamic viscosity **(b)** calculated from MSD of TERF1. **(c)** Quantification of histone 2B (H2B) displacement during cell detachment. n=9 cells. Representative H2B displacement map **(d)** and quantification **(e)** of H2B displacement under 0, 1, 2, 3, and 4 dyne/cm^2 FSS. n=11 cells for each condition. **(f)** Schematic of H2B displacement induced by FSS. **(g)** Normalized force transmission efficiency (FTE) of non-adherent MCF-7 cells under FSS without shear stress pretreatment. n=11 cells for each condition. **(h)** Normalized FTE of non-adherent MDA-MB-231 cells under FSS without shear stress pretreatment. n=14 cells for each condition. **(i)** Cortex thickness before and after shear treatment. n=20 cells for each condition. Data were summarized from 3 biological repeats and presented as mean ± SEM. The one-way ANOVA followed by Tukey's post-hoc test was adopted for comparison in **(c)**, **(e)** and **(g-h)**. Unpaired student *t*-test was adapted for comparison in **(i)**. ns, no significant difference; *, p <0.05; **, p < 0.01; and ***, p < 0.001.

Figure S3 Cell and nuclear size are not confounding factors in H2B displacement. (a-b) Quantification of H2B displacement divided by nuclear **(a)** and cell **(b)** radius of non-adherent tumor cells after the pre-treatment of 0, 0.5, 1, 2 and 4 dyne/cm^2 FSS for an hour. n=11, 13, 14, 9 and 14 cells for 0, 0.5, 1, 2 and 4 dyne/cm^2 FSS pre-treatment, respectively. **(c-d)** Normalized FTE to nuclear **(c)** and cell **(d)** radius of non-adherent tumor cells after the pre-treatment of 0, 0.5, 1, 2 and 4 dyne/cm^2 FSS for an hour. n=11, 13, 14, 9 and 14 cells for 0, 0.5, 1, 2 and 4 dyne/cm^2 FSS pre-treatment, respectively. Data were summarized from 3 biological repeats and presented as mean ± SEM. Kruskal–Wallis one-way ANOVA followed by Mann–Whitney U-test with Bonferroni correction was adopted for comparison in **(a-d)**. ns, no significant difference; *, p <0.05; **, p < 0.01; ***, p < 0.001.

Figure S4 PLL-attached tumor cells exhibit p-MLC subcellular redistribution and reduced FTE to increasing FSS. Representative immunofluorescence images **(a)** and quantification of phosphorylated myosin light chain (p-MLC) in whole cells **(b)**, cortex

(c) and cytoplasm **(d)**, and of p-MLC cortical/cytoplasmic ratio **(e)** after the treatment with 0, 0.5, 1, 2 and 4 dyne/cm^2 FSS for an hour. n=21, 25, 27, 21 and 23 cells for 0, 0.5, 1, 2 and 4 dyne/cm^2, respectively. Scale bar, 5 μm. Representative immunofluorescence images **(f)** and quantification of p-MLC in cortex **(g)** and cytoplasm **(h)** after the attachment to PLL-coated substrate for the indicated durations. n=42, 43 and 48 cells for PLL 0 min, PLL 30 min and PLL 60 min, respectively. Scale bar, 5 μm. **(i)** Normalized FTE and cortical p-MLC of PLL-attached tumor cells under various levels of FSS. n=21, 25, 27, 21 and 23 cells for cortical p-MLC under 0, 0.5, 1, 2 and 4 dyne/cm^2, respectively; n=12, 13, 14, 19 and 14 cells for FTE under 0, 0.5, 1, 2 and 4 dyne/cm^2, respectively. Representative immunofluorescence images **(j)** and quantification **(k)** of p-MLC in PLL-attached tumor cells after the treatment of 4 dyne/cm^2 FSS for 0, 10 and 20 minutes. n=20, 23 and 21 cells for 0, 10 and 20 minutes, respectively. Scale bar, 5 μm. **(l)** Correlation between cytoplasmic p-MLC and normalized FTE in PLL-attached tumor cells under 4 dyne/cm^2 FSS. n=49, 23, 21, 20 and 41 cells for cytoplasmic p-MLC under 0, 10, 20, 30 and 60 minutes, respectively; n=23, 12, 13, 12 and 10 cells for FTE under 0, 10, 20, 30 and 60 minutes, respectively. Data were summarized from 3 biological repeats and presented as mean ± SEM. The one-way ANOVA followed by Tukey's post-hoc test was adopted for comparison in **(b-c)**, **(e)**, **(g)** and **(l)**. Kruskal–Wallis one-way ANOVA followed by Mann–Whitney U-test with Bonferroni correction was adopted for comparison in **(d)**, **(h)** and **(k)**. ns, no significant difference; *, p <0.05 and ***, p < 0.001.

Figure S5 The increase of cytoplasmic p-MLC is correlated with the reduction of FTE in non-adherent tumor cells under high FSS. Representative immunofluorescence images **(a)** and quantification of p-MLC in whole cells **(b)**, cortex **(c)** and cytoplasm **(d)** in non-adherent tumor cells within the tubing after 10 dyne/cm^2 FSS treatment for 0, 1, 2, 4 and 8 hours. n=42, 41, 38, 41 and 40 cells for 0, 1, 2, 4 and 8 hours, respectively. Scale bar, 5 μm. **(e)** p-MLC subcellular distribution in **(a)**. **(f)** Correlation between cytoplasmic p-MLC and normalized FTE under 10 dyne/cm^2 FSS. n=42, 95, 30, 41 and 40 cells for cytoplasmic p-MLC after 0.5, 1, 2 and 4 hours,

respectively; n=23, 29, 30, 25 and 21 cells for FTE after 0.5, 1, 2 and 4 hours, respectively. Data were summarized from 3 biological repeats and presented as mean ± SEM. The one-way ANOVA followed by Tukey's post-hoc test was adopted for comparison in **(b-c)** and **(f)**. Kruskal–Wallis one-way ANOVA followed by Mann–Whitney U-test with Bonferroni correction was adopted for comparison in **(d)**. ns, no significant difference; **, p <0.01 and ***, p < 0.001.

Figure S6 Inhibition of cytoplasmic p-MLC rescues FTE of non-adherent tumor cells under high FSS. Representative immunofluorescence images **(a)** and quantification of p-MLC in whole cell **(b)** cortex **(c)** and cytoplasm **(d)**, and of p-MLC cortical/cytoplasmic ratio **(e)** after the shear and 4-HAP treatments. n=22, 19, 21 and 21 cells for 0 dyne/cm^2 with ethanol, 0 dyne/cm^2 with 4-HAP, 4 dyne/cm^2 ethanol, and 4 dyne/cm^2 with 4-HAP, respectively. Scale bar, 5 μm. **(f)** Quantification of γ-H2Ax after ethanol treatment. Hydrogen peroxide was used as positive control. Cells were treated with 0.01% ethanol for an hour or 100 μM H_2O_2 for thirty minutes. **(g)** Normalized FTE after shear and 4-HAP treatments. n=12, 14 and 13 cells for 0 dyne/cm^2 with ethanol, 4 dyne/cm^2 with ethanol, and 4 dyne/cm^2 with 4-HAP, respectively. Representative immunofluorescence images **(h)** and quantification of p-MLC in whole cell **(i)** and cytoplasm **(j)** after the shear and Y27632 treatments. n=41, 42 and 40 cells for 0 dyne/cm^2 with DMSO, 4 dyne/cm^2 with DMSO, and 4 dyne/cm^2 with Y27632, respectively. Non-adherent tumor cells were treated under 4 dyne/cm^2 FSS together with Y27632. Scale bar, 5 μm. **(k)** Normalized FTE after shear and Y27632 treatments. n=11, 12 and 13 cells for 0 dyne/cm^2 with DMSO, 4 dyne/cm^2 with DMSO, and 4 dyne/cm^2 with Y27632, respectively. Non-adherent tumor cells were treated under 4 dyne/cm^2 FSS together with Y27632. Data were summarized from 3 biological repeats and presented as mean ± SEM. The one-way ANOVA followed by Tukey's post-hoc test was adopted for comparison in **(b-d)**, **(f-g)** and **(i)**. Kruskal–Wallis one-way ANOVA followed by Mann–Whitney U-test with Bonferroni correction was adopted for comparison in **(e)** and **(j-k)**. ns, no significant difference; *, p <0.05, **, p <0.01 and ***, p < 0.001.

Figure S7 Knockdown *MYH9* or *MYH10* rescues FTE of non-adherent tumor cells under high FSS. Representative immunofluorescence images **(a)** and quantification of p-MLC in whole cell **(b),** cortex **(c),** and cytoplasm **(d)** after knocking down *MYH10*. n=44, 42, 48 and 51 cells from 3 biological repeats for Control, si-*MYH10*-1, si-*MYH10*-2 and si-*MYH10*-3, respectively. Scale bar, 5 μm. **(e)** mRNA expression of *MYH10* after knocking down *MYH10*. n=3 biological repeats for each condition. **(f)** Normalized FTE after knocking down of *MYH10* under 4 dyne/cm^2 FSS. *MYH10* was knocked down two days before FSS treatment. n=12 cells from 3 biological repeats for each condition. **(g)** Normalized FTE after knocking down of *MYH10*. n=12 and 16 cells from 3 biological repeats for Control and si-*MYH10*, respectively. Representative immunofluorescence images **(h)** and quantification of p-MLC in whole cell **(i)**, cortex **(j)**, and cytoplasm **(k)** after knocking down *MYH9*. n=30, 32, 33 and 31 cells from 3 biological repeats for Control, si-*MYH9*-1, si-*MYH9*-2 and si-*MYH9*-3, respectively. Scale bar, 5 μm. **(l)** mRNA expression of *MYH9* after knocking down *MYH9*. n=3 biological repeats for each condition. **(m)** Normalized FTE after knocking down of *MYH9* under 4 dyne/cm^2 FSS. *MYH9* was knocked down two days before FSS. n=13 cells from 3 biological repeats for each condition. **(n)** Normalized FTE after knocking down of *MYH9*. n=14 cells from 3 biological repeats for each condition. Data were presented as mean ± SEM. The one-way ANOVA followed by Tukey's post-hoc test was adopted for comparison in **(b)**, **(d-e)** and **(l)**. Kruskal–Wallis one-way ANOVA followed by Mann–Whitney U-test with Bonferroni correction was adopted for comparison in **(c)**, **(f)**, **(i-k)** and **(m)**. Mann–Whitney U-test was adapted for comparison in **(g)** and **(n)**. ns, no significant difference; *, p <0.05, **, p <0.01 and ***, p < 0.001.

Figure S8 Cortical myosin II is dispensable for force transmission of non-adherent tumor cells. Representative immunofluorescence images **(a)** and quantification of p-MLC in cortex **(b)** and cytoplasm **(c)** after shear and Blebbistatin (Bleb) treatment. PLL-attached tumor cells were treated under FSS together with Bleb. n=21, 22, 22 and 20 cells for 0 dyne/cm^2 with DMSO, 0 dyne/cm^2 with Bleb, 4 dyne/cm^2 with DMSO and 4 dyne/cm^2 with Bleb, respectively. Scale bar, 5 μm. **(d)** Normalized FTE after

shear and Bleb treatments. PLL-attached tumor cells were treated under FSS together with Bleb. n=6, 12 and 12 cells for 0 dyne/cm^2 with DMSO, 4 dyne/cm^2 with DMSO and 4 dyne/cm^2 with Bleb, respectively. Representative immunofluorescence images **(e)** and quantification of p-MLC in cortex **(f)** and cytoplasm **(g)** after shear and ML-7 treatment. PLL-attached tumor cells were treated under FSS together with ML-7. n=44, 51, 47 and 47 cells for 0 dyne/cm^2 with DMSO, 0 dyne/cm^2 with ML-7, 4 dyne/cm^2 with DMSO and 4 dyne/cm^2 with ML-7, respectively. Scale bar, 5 μm. **(h)** Normalized FTE after shear and ML-7 treatments. n=13 cells for each condition. PLL-attached tumor cells were treated under FSS together with ML-7. Quantification of p-MLC in cortex **(i)** and cytoplasm **(j)** after knocking down *MLCK* under 4 dyne/cm^2 FSS. *MLCK* was knocked down two days before shear treatment. n=29, 25, 41 and 33 cells for 0 dyne/cm^2 with Control, 0 dyne/cm^2 with si-*MLCK*, 4 dyne/cm^2 with Control and 4 dyne/cm^2 with si-*MLCK*, respectively. **(k)** Normalized FTE after knocking down *MLCK* under 4 dyne/cm^2 FSS. *MLCK* was knocked down two days before shear treatment. n=8 cells for each condition. **(l)** Calcein AM/PI assay after pharmacologic treatments. Data were summarized from 3 biological repeats and presented as mean ± SEM. The one-way ANOVA followed by Tukey's post-hoc test was adopted for comparison in **(b)**, **(d)**, and **(h).** Kruskal–Wallis one-way ANOVA followed by Mann–Whitney U-test with Bonferroni correction was adopted for comparison in **(c)**, **(f-g)** and **(i-k)**. ns, no significant difference; *, p <0.05, **, p <0.01 and ***, p < 0.001.

Figure S9 The regulation of force transmission by cytoplasmic myosin is independent of FSS. Representative immunofluorescence images **(a)** and quantification of p-MLC in whole cells, cortex and cytoplasm **(b)** after the treatment of Y27632 for 10 min. n=57 and 67 cells for DMSO and Y27632 group, respectively. Scale bar, 5 μm. **(c)** Normalized FTE of non-adherent tumor cells after the pre-treatment in **(b)**. n=11 and 12 cells for DMSO and Y27632 group, respectively. Representative immunofluorescence images **(d)** and quantification of p-MLC in whole cells, cortex and cytoplasm **(e)** after calyculin A treatment for an hour. n=23 and 27 cells for DMSO and Calyculin A group, respectively. Scale bar, 5 μm. **(f)** Normalized FTE of non-

adherent tumor cells after the pre-treatment in **(e)**. n=13 cells for each condition. Data were summarized from 3 biological repeats and presented as mean ± SEM. Unpaired student *t*-test was adopted for comparison in **(b)**. Mann–Whitney U-test was adopted for comparison in **(c)**, **(e)** and **(f)**. ns, no significant difference; **, $p < 0.01$ and ***, $p < 0.001$.

Figure S10 The influence of cytoplasmic myosin on force transmission using Nesprin-based tension sensor. Representative FRET images **(a)** and quantification **(b)** of FRET index of Nesprin-based tension senor after Bleb treatment in adherent MCF-7 cells. n=19 and 21 cells for DMSO and Bleb treatment, respectively. Scale bar, 5 μm. **(c)** Dynamic changes of FRET index of Nesprin-based tension senor under shear treatment. MCF-7 cells were transfected with Nesprin-based FRET biosensor, attached to PLL-coated microfluidic chip, and then treated under 0, 0.5 or 1 dyne/cm^2 FSS. n=3, 7 and 7 cells for 0 dyne/cm^2, 0.5 dyne/cm^2 and 1 dyne/cm^2, respectively. Representative FRET images **(d)** and quantification **(e)** of FRET index of Nesprin-based tension senor after knocking down *MY*H10 in non-adherent tumor cells without shear treatment. n=43 and 45 cells for Control and si-*MYH10,* respectively. Scale bar, 5 μm. Representative FRET images **(f)** and quantification **(g)** of FRET index of Nesprin-based tension senor after knocking down *MYH10* in PLL-attached tumor cells under 0.5 dyne/cm^2 FSS. *MYH10* was knocked down two days before experiment. n=6 and 12 cells for Control and si-*MYH10*, respectively. Scale bar, 5 μm. Data were summarized from 3 biological repeats and presented as mean ± SEM. Unpaired student *t*-test was adopted for comparison in **(b)**, **(c)**, **(e)** and **(g)**. ns, no significant difference; *, $p <0.05$, **, $p <0.01$ and ***, $p < 0.001$.

Figure S11 F-actin and microtubule are not responsible for the reduction of force transmission in non-adherent tumor cells under high FSS. Representative immunofluorescence images **(a)** and quantification F-actin in whole cell **(b)**, cortex and cytoplasm **(c)** after shear treatment. n=64 and 68 cells for 0 dyne/cm^2 and 4 dyne/cm^2, respectively. Scale bar, 5 μm. **(d)** Normalized FTE after 100 nM Cytochalasin D (Cyto

D) treatment. Non-adherent tumor cells were treated with Cyto D. n=7 cells for each condition. **(e)** Normalized FTE after shear and CytoD treatment. Non-adherent tumor cells were treated under FSS together with CytoD. n=14 cells for each condition. Representative immunofluorescence images **(f)** and quantification **(g)** of F-actin after shear and Y27632 treatment. PLL-attached tumor cells were treated under FSS together with Y27632. n=30, 33, 29 and 31 cells for 0 dyne/cm^2 with DMSO, 0 dyne/cm^2 with Y27632, 4 dyne/cm^2 with DMSO, and 4 dyne/cm^2 with Y27632, respectively. Scale bar, 5 μm. Representative immunofluorescence images **(h)** and quantification **(i)** of α-tubulin after shear treatment. n=60, 60 and 64 cells for 0 dyne/cm^2, 1 dyne/cm^2 and 4 dyne/cm^2, respectively. Scale bar, 5 μm. **(j)** Normalized FTE after shear and nocodazole treatment. PLL-attached tumor cells were treated under FSS together with 1 μM nocodazole. n=14 cells for each condition. Data were summarized from 3 biological repeats and presented as mean ± SEM. Kruskal–Wallis one-way ANOVA followed by Mann–Whitney U-test with Bonferroni correction was adopted for comparison in **(e)**, **(g)** and **(i-j)**. Mann–Whitney U-test was adopted for comparison in **(b-d)**. ns, no significant difference; *, p <0.05, **, p <0.01 and ***, p < 0.001.

Figure S12 DNA damage and cell cycle do not influence myosin II subcellular localization and force transmission of non-adherent tumor cells. Representative immunofluorescence images **(a)** and quantification **(b)** of the percentage of γ-H2AX+ cells after Doxorubicin treatment. Non-adherent MCF-7 cells were treated with 2 μM Doxorubicin for 30 or 60 minutes. n=3 biological repeats for each condition. Scale bar, 5 μm. Representative immunofluorescence images **(c)** and quantification **(d)** of PI after Doxorubicin treatment. n=19, 19, 20 and 21 cells from 3 biological repeats for DMSO, Doxorubicin 30 min, Doxorubicin 60 min and permeablized, respectively. Scale bar, 5 μm. Representative immunofluorescence images **(e)** and quantification of p-MLC in cortex **(f)** and cytoplasm **(g)** after Doxorubicin treatment. n=41, 42 and 45 cells from 3 biological repeats for DMSO, Doxorubicin 30 min and Doxorubicin 60 min, respectively. Scale bar, 5 μm. **(h)** Normalized FTE after Doxorubicin treatment. n=12, 12 and 10 cells from 3 biological repeats for DMSO, Doxorubicin 30 min and

Doxorubicin 60 min, respectively. Representative immunofluorescence images **(i)** and quantification of p-MLC in cortex **(j)** and cytoplasm **(k)** after low serum treatment. n=30, 35, 38 and 35 cells from 3 biological repeats for 0 dyne/cm^2 with 10% FBS, 0 dyne/cm^2 with 1% FBS, 4 dyne/cm^2 with 10% FBS, and 4 dyne/cm^2 with 1% FBS, respectively. 1% FBS were added two days before FSS to induce cell cycle arrest. Scale bar, 5 μm. **(l)** Normalized FTE after cell cycle arrest. n=7 cells from 3 biological repeats for each condition. Data were presented as mean ± SEM. The one-way ANOVA followed by Tukey's post-hoc test was adopted for comparison in **(b)**, **(h)** and **(l)**. Kruskal–Wallis one-way ANOVA followed by Mann–Whitney U-test with Bonferroni correction was adopted for comparison in **(d)**, **(f-g)** and **(j-k)**. ns, no significant difference; *, p <0.05, **, p <0.01 and ***, p < 0.001.

Figure S13 Cell-cell adhesion does not influence myosin II subcellular localization, force transmission or cell viability of non-adherent tumor cells under FSS. Representative immunofluorescence images **(a)** and the quantification of p-MLC in whole cells **(b)**, cortex **(c)**, cytoplasm **(d)**, and the cortical/cytoplasmic ratio **(e)** after BAS00602705 and FSS treatment for an hour. 10 μM BAS00602705 was used to treat cells 24 hours before FSS. n=31, 26 and 33 cells for 0 dyne/cm^2 with DMSO, 4 dyne/cm^2 with DMSO and 4 dyne/cm^2 with BAS00602705, respectively. Scale bar, 5 μm. **(f)** Normalized FTE after shear and BAS00602705 treatment. n-10 cells for each condition. **(g)** Quantification of E-cadherin after Y27632 treatment. n= 31 and 35 cells for DMSO and Y27632, respectively. Representative images **(h)** and quantification **(i)** of the percentage of PI$^+$ cells after BAS00602705 and shear treatment. n=9 views for each condition. Scale bar, 100 μm. Data were summarized from 3 biological repeats and presented as mean ± SEM. The one-way ANOVA followed by Tukey's post-hoc test was adopted for comparison in **(b)**. Kruskal–Wallis one-way ANOVA followed by Mann–Whitney U-test with Bonferroni correction was adopted for comparison in **(c-f)** and **(i)**. Mann–Whitney U-test was adopted for comparison in **(g)**. ns, no significant difference; *, p < 0.05; **, p < 0.01; ***, p < 0.001.

Figure S14 Cytoplasmic but not cortical myosin decreases the interaction of myosin with actin under high FSS. Representative immunofluorescence images **(a)** and quantification **(b)** of Pearson correlation coefficient of p-MLC and F-actin in cytoplasm. PLL-attached tumor cells were treated under 0, 1 and 4 dyne/cm^2 FSS for an hour. n=93, 90 and 95 cells for 0 dyne/cm^2, 1 dyne/cm^2, and 4 dyne/cm^2, respectively. Scale bar, 5 μm. Representative immunofluorescence images **(c)** and quantification **(d)** of Pearson correlation coefficient of p-MLC and F-actin in cytoplasm. PLL-attached tumor cells were treated under 4 dyne/cm^2 FSS, which was followed by the treatment with Y27632. n=51, 59 and 58 cells for 0 dyne/cm^2 with DMSO, 4 dyne/cm^2 with DMSO and 4 dyne/cm^2 with Y27632, respectively. Scale bar, 5 μm. Representative PLA images **(e)** and the quantification **(f)** of PLA dots in cytoplasm after shear and pharmacologic treatments. The interaction between myosin IIA and β-actin was detected. White arrows indicated typical PLA dots. n=23 cells for each condition. Scale bar, 5 μm. Data were summarized from 3 biological repeats and presented as mean ± SEM. The one-way ANOVA followed by Tukey's post-hoc test was adopted for comparison in **(b)** and **(f)**. Kruskal–Wallis one-way ANOVA followed by Mann–Whitney U-test with Bonferroni correction was adopted for comparison in **(d)**. ns, no significant difference; **, $p < 0.01$; ***, $p < 0.001$.

Figure S15 Lamina-chromatin linkage does not contribute to the reduction of force transmission. Representative immunofluorescence images **(a)** and quantification **(b)** of BAF under FSS. Non-adherent tumor cells were treated by FSS for an hour. n=34, 33 and 35 cells for 0 dyne/cm^2, 1 dyne/cm^2 and 4 dyne/cm^2, respectively. Scale bar, 5 μm. Representative immunofluorescence images **(c)** and quantification **(d)** of HP1 under FSS. Non-adherent tumor cells were treated by FSS for an hour. n=31, 33 and 40 cellsfor 0 dyne/cm^2, 1 dyne/cm^2 and 4 dyne/cm^2, respectively. Scale bar, 5 μm. Data were summarized from 3 biological repeats and presented as mean ± SEM. The one-way ANOVA followed by Tukey's post-hoc test was adopted for comparison in **(b)** and **(d)**. ns, no significant difference

Figure S16 Lamin A/C dephosphorylation responds rapidly to FSS in non-adherent tumor cells. Representative western blot images **(a)** and quantification **(b)** of p-MLC under 10 dyne/cm^2 FSS.Non-adherent tumor cells were treated by FSS for an hour. n=4 biological repeats. Representative immunofluorescence images **(c)** and quantification **(d)** of Lamin A/C under 4 dyne/cm^2 FSS. n= 30, 27 and 26 cells for 0, 2 and 5 min, respectively. Scale bar, 5 μm. Representative immunofluorescence images **(e)** and quantification **(f)** of phosphorylated Lamin A/C under 4 dyne/cm^2 FSS. n= 30, 31 and 43 cells for 0, 1 and 2 min, respectively. Scale bar, 5 μm. Representative immunofluorescence images **(g)** and quantification **(h)** of the normalized ratio of phosphorylated Lamin A/C and total Lamin A/C under 4 dyne/cm^2 FSS. n= 29, 29 and 32 cells for 0, 2 and 5 min, respectively. Scale bar, 5 μm. Representative immunofluorescence images **(i)** and quantification **(j)** of phosphorylated Lamin A/C under 4 dyne/cm^2 FSS. n= 30, 29, 29 and 29 cells for 0, 10, 30 and 60 min, respectively. Scale bar, 5 μm. Representative FLIM images **(k)** and quantification **(l)** of ER-Flipper in PLL-attached tumor cells under various levels of FSS. n=30, 29 and 30 cells for 0, 1 and 4 dyne/cm^2, respectively. Scale bar, 5 μm. Data were summarized from 3 biological repeats and presented as mean ± SEM. The one-way ANOVA followed by Tukey's post-hoc test was adopted for comparison in **(d)**, **(f)** and **(l)**. Kruskal–Wallis one-way ANOVA followed by Mann–Whitney U-test with Bonferroni correction was adopted for comparison in **(h)** and **(j)**. ns, no significant difference; *, p <0.05, **, p <0.01 and ***, p < 0.001.

Figure S17 The dynamics of Lamin A/C under FSS. Representative fluorescence images **(a)** and quantification **(b)** of Lamin A/C under FSS. n=9 and 17 cells from 3 biological repeats for 0 dyne/cm^2 and 4 dyne/cm^2, respectively. Scale bar, 5 μm. **(c)** Western blot of Lamin A/C under the treatment of 10 dyne/cm^2 FSS for the indicated durations. Data were presented as mean ± SEM. Unpaired student *t*-test was adopted for comparison in **(b)**.

Figure S18 Lamin A/C degradation, rather than translation, regulates its expression level under FSS. (a) Western blot of Lamin A/C after shear and puromycin treatment. 1 µg/ml puromycin was used to treat cells for 30 minutes prior to FSS. **(b)** Western blot of Lamin A/C after shear and Wortmannin treatment. 1 µM AKT inhibitor Wortmannin was used to treat cells for 2 hours prior to FSS. **(c)** Western blot of Lamin A/C after gene silencing or overexpression. Knockdown and overexpression efficiency were quantified at 72 hours after transfection.

Figure S19 The influence of Lamin A/C overexpression on cytoplasmic myosin activity and force transmission in non-adherent tumor cells. Representative immunofluorescence images **(a)** and quantification of p-MLC in whole cell **(b)**, cortex **(c)** and cytoplasm **(d)** after shear and *LMNA* overexpression. n=44, 47, 47 and 56 cells for 0 dyne/cm^2 with empty vectors, 0 dyne/cm^2 with *LMNA* overexpression, 4 dyne/cm^2 with empty vectors, and 4 dyne/cm^2 with *LMNA* overexpression, respectively. MCF-7 cells were transfected with empty vector or *LMNA* plasmids two days before experiment. Scale bar, 5 µm. Normalized FTE after *LMNA* overexpression **(e)** and knockdown **(f)**. MCF-7 cells were transfected with empty vector, *LMNA* plasmids, or *LMNA* siRNA two days before experiment. n=12 cells for each condition in **(e)** and 13 for each condition in **(f)**. Representative immunofluorescence images **(g)** and quantification p-MLC in cortex **(h)** and cytoplasm **(i)** after *LMNA* knockdown under 1 dyne/cm^2 FSS. *LMNA* was knocked down two days before experiment. n=42, 42 and 43 cells for 0 dyne/cm^2 with Control, 1 dyne/cm^2 with Control and 1 dyne/cm^2 with si-*LMNA* respectively. Scale bar, 5 µm. **(j)** Normalized FTE after *LMNA* knockdown under 1 dyne/cm^2 FSS. n=12, 12 and 14 cells for 0 dyne/cm^2 with Control, 1 dyne/cm^2 with Control and 1 dyne/cm^2 with si-*LMNA* respectively. Data were summarized from 3 biological repeats and presented as mean ± SEM. The one-way ANOVA followed by Tukey's post-hoc test was adopted for comparison in **(b)** and **(d)**. Kruskal–Wallis one-way ANOVA followed by Mann–Whitney U-test with Bonferroni correction was adopted for comparison in **(c)** and **(h-j)**. Mann–Whitney U-test was adopted for comparison in **(e-f)**. ns, no significant difference; *, p <0.05 and ***, p < 0.001.

Figure S20 Extracellular calcium is dispensable for myosin II activation and force transmission of non-adherent tumor cells under FSS. Representative immunofluorescence images **(a)** and quantification **(b)** of intracellular calcium level measured by GCaMP6f calcium senor after Yoda1 treatment. PLL-attached tumor cells were treated with 2 μM Yoda1 for an hour. n=20 and 22 cells for DMSO and Yoda1, respectively. Scale bar, 5 μm. Representative immunofluorescence images **(c)** and quantification **(d)** of intracellular calcium level measured by GCaMP6f calcium senor after 4 dyne/cm^2 FSS treatment for an hour. n=43 and 39 cells for 0 dyne/cm^2 and 4 dyne/cm^2, respectively. Scale bar, 5 μm. **(e)** Intracellular calcium level measured by GCaMP6f calcium sensor under 1 dyne/cm^2 FSS. Non-adherent tumor cells were transfected with GCaMP6f calcium sensor, treated with DMSO, si-*LMNA* or Thapsigargin (Tg), and then subjected to 1 dyne/cm^2 FSS. Medium was pretreated with 1 mM EGTA to chelate extracellular calcium before FSS. n=12, 11, 9 and 14 cells for DMSO, si-*LMNA*, EGTA and Thapsigargin, respectively. Representative immunofluorescence images **(f)** and quantification of p-MLC in whole cells **(g)** after the treatment of FSS and Tg. Non-adherent tumor cells were pre-treated with Tg for two days and then subjected to 0 or 4 dyne/cm^2 FSS for an hour. n=47, 50, 48 and 46 cells for 0 dyne/cm^2 with DMSO, 0 dyne/cm^2 with Tg, 4 dyne/cm^2 with DMSO and 4 dyne/cm^2 with Tg, respectively. Scale bar, 5 μm. Representative immunofluorescence images **(h)** and quantification of p-MLC in whole cell **(i)** and cytoplasm **(j)** after shear and EGTA treatment. EGTA was added into the medium before FSS. n=42, 47, 52 and 53 cells for 0 dyne/cm^2 with DMSO, 0 dyne/cm^2 with EGTA, 4 dyne/cm^2 with DMSO, and 4 dyne/cm^2 with EGTA, respectively. Scale bar, 5 μm. **(k)** Normalized FTE after EGTA treatment under 4 dyne/cm^2 FSS. n=14, 12 and 13 cells for 0 dyne/cm^2 with DMSO, 4 dyne/cm^2 with DMSO, and 4 dyne/cm^2 with EGTA, respectively. Data were summarized from 3 biological repeats and presented as mean ± SEM. The one-way ANOVA followed by Tukey's post-hoc test was adopted for comparison in **(e)** and **(i-j)**. Kruskal–Wallis one-way ANOVA followed by Mann–Whitney U-test with Bonferroni correction was adopted for comparison in **(g)** and **(k)**. Mann–Whitney U-

test was adopted for comparison in **(b)** and **(d)**. ns, no significant difference; *, p <0.05, **, p <0.01 and ***, p < 0.001.

Figure S21 Calcium release from ER does not affect Lamin A/C activity. Quantification of p-MLC in cell cortex **(a)** and cytoplasm **(b)** after shear and Thapsigargin (Tg) treatment. MCF-7 cells were treated with Tg two days before shear treatment. n= 31, 34, 33 and 33 cells for 0 dyne/cm^2 with DMSO, 0 dyne/cm^2 with Tg, 1 dyne/cm^2 with DMSO, and 1 dyne/cm^2 with Tg, respectively. Representative immunofluorescence images **(c)** and quantification **(d)** of Lamin A/C after shear and Tg treatment. MCF-7 cells were treated with Tg two days before FSS. n=33, 29, 31 and 30 cells for 0 dyne/cm^2 with DMSO, 0 dyne/cm^2 with Tg, 4 dyne/cm^2 with DMSO, and 4 dyne/cm^2 with Tg, respectively. Scale bar, 5 μm. **(e)** Quantification of excess of perimeter of nuclear envelope after shear and Tg treatment. MCF-7 cells were treated with Tg two days before shear treatment. n=31, 29, 31 and 30 cells for 0 dyne/cm^2 with DMSO, 0 dyne/cm^2 with Tg, 4 dyne/cm^2 with DMSO, and 4 dyne/cm^2 with Tg, respectively. **(f)** Quantification of phosphorylated Lamin A/C after shear and Tg treatment. n=31, 30, 32 and 31 cells for 0 dyne/cm^2 with DMSO, 0 dyne/cm^2 with Tg, 4 dyne/cm^2 with DMSO and 4 dyne/cm^2 with Tg, respectively. Representative immunofluorescence images **(g)** and quantification of p-MLC in whole cell **(h)**, cortex **(i),** and cytoplasm **(j)** after *LMNA* overexpression in Tg-treated non-adherent tumor cells. MCF-7 cells were treated with Tg two days before *LMNA* overexpression. n=45, 44 and 49 cells for empty vectors, *LMNA* overexpression and *LMNA* overexpression with Tg, respectively. Scale bar, 5 μm. **(k)** Normalized FTE after Tg treatment in *LMNA* overexpressed non-adherent tumor cells. MCF-7 cells were treated with Tg two days before *LMNA* overexpression. n=13, 12 and 12 cells for empty vectors, *LMNA* overexpression and *LMNA* overexpression with Tg, respectively. Data were summarized from 3 biological repeats and presented as mean ± SEM. The one-way ANOVA followed by Tukey's post-hoc test was adopted for comparison in **(a)** and **(i-j)**. Kruskal–Wallis one-way ANOVA followed by Mann–Whitney U-test with

Bonferroni correction was adopted for comparison in **(b)**, **(d-f)**, **(h)** and **(k)**. ns, no significant difference; *, p <0.05, **, p <0.01 and ***, p < 0.001.

Figure S22 Nesprin is indispensable in mechanical responses of non-adherent tumor cells to FSS. (a) Normalized FTE after DN-KASH treatment in non-adherent tumor cells. PLL-attached tumor cells were transfected with ΔPPPL or DN-KASH prior to the treatment under FSS. n=14 and 15 cells for ΔPPPL and DN-KASH, respectively. Representative immunofluorescence images **(b)** and quantification **(c)** of Lamin A/C, excess of perimeter of nuclear envelope **(d),** phosphorylated Lamin A/C **(e),** and intracellular calcium level **(f)** after shear and DN-KASH treatment. PLL-attached tumor cells were transfected with ΔPPPL or DN-KASH prior to the treatment with FSS. n=25, 24, 25 and 31 cells in (**c**), 25, 25, 25 and 26 cells in **(d)**, and 30, 30, 30 and 29 cells in **(e)** for 0 dyne/cm^2 with ΔPPPL, 0 dyne/cm^2 with DN-KASH, 4 dyne/cm^2 with ΔPPPL and 4 dyne/cm^2 with DN-KASH, respectively. n=9 cells for each condition in **(f)**. Scale bar, 5 μm. Representative immunofluorescence images **(g)** and quantification of p-MLC in whole cell **(h)**, cortex **(i),** and cytoplasm **(j)** after shear and DN-KASH treatment. PLL-attached tumor cells were transfected with ΔPPPL or DN-KASH prior to the treatment under FSS. n=28, 30, 27 and 31 cells for 0 dyne/cm^2 with ΔPPPL, 0 dyne/cm^2 with DN-KASH, 4 dyne/cm^2 with ΔPPPL and 4 dyne/cm^2 with DN-KASH, respectively. Scale bar, 5 μm. Data were summarized from 3 biological repeats and presented as mean ± SEM. Mann–Whitney U-test was adopted for comparison in **(a)**. Kruskal–Wallis one-way ANOVA followed by Mann–Whitney U-test with Bonferroni correction was adopted for comparison in **(c-e)** and **(h-j)**. Unpaired student *t*-test was adopted for comparison in **(f)**. ns, no significant difference; *, p <0.05, **, p <0.01 and ***, p < 0.001.

Figure S23 MDA-MB-231 cells show more activated myosin in cytoplasm and lower FTE compared with MCF-7 cells. Representative immunofluorescence images **(a)** and quantification of p-MLC in whole cell **(b)**, cortex **(c),** and cytoplasm **(d)** in non-adherent MCF-7 and MDA-MB-231 cells. n=37, 35, 36 and 39 cells for MCF-7 cells

under 0 dyne/cm^2 FSS, MDA-MB-231 cells under 0 dyne/cm^2 FSS, MCF-7 cells under 4 dyne/cm^2 FSS and MDA-MB-231 cells under 4 dyne/cm^2 FSS, respectively. Scale bar, 5 μm. **(e)** Normalized FTE of non-adherent MCF-7 and MDA-MB-231 cells. n=7 cells for each type of cell. **(f)** Normalized FTE of non-adherent MDA-MB-231 cells after the pre-treatment of 0, 1 and 4 dyne/cm^2 FSS for an hour. n=15, 14 and 15 cells for 0, 1 and 4 dyne/cm^2 FSS, respectively. Representative immunofluorescence images **(g)** and quantification **(h)** of Lamin A/C in non-adherent MDA-MB-231 cells under various levels of FSS. n=35, 32 and 41 cells for 0, 1 and 4 dyne/cm^2, respectively. Scale bar, 5 μm. **(i)** Quantification of excess of perimeter of nuclear envelope in non-adherent MDA-MB-231 cells under various levels of FSS. n=35, 32 and 50 cells for 0, 1 and 4 dyne/cm^2, respectively. Data were summarized from 3 biological repeats and presented as mean ± SEM. Kruskal–Wallis one-way ANOVA followed by Mann–Whitney U-test with Bonferroni correction was adopted for comparison in **(b-d)**, **(f)** and **(h-i)**. Mann–Whitney U-test was adopted for comparison in **(e)**. ns, no significant difference; *, p <0.05, **, p <0.01 and ***, p < 0.001.

Figure S24 MDA-MB-231 cells exhibit similar nuclear mechanosensing and calcium release from ER under FSS. Representative immunofluorescence images **(a)** and quantification **(b)** of p-MLC of non-adherent MDA-MB-231 cells after shear and si-*LMNA* treatment. *LMNA* was knocked down two days before FSS treatment. n=32, 34, 31 and 35 cells for 0 dyne/cm^2 with Control, 0 dyne/cm^2 with si-*LMNA*, 4 dyne/cm^2 with Control and 4 dyne/cm^2 with si-*LMNA*, respectively. Scale bar, 5 μm. **(c)** Normalized FTE of non-adherent MDA-MB-231 cells after shear and si-*LMNA* treatment. n=14 cells for each condition. **(d)** Intracellular calcium level measured by GCaMP6f calcium sensor under 4 dyne/cm^2 FSS. Non-adherent MDA-MB-231 were transfected with GCaMP6f calcium sensor and then treated with DMSO, si-*LMNA* or Thapsigargin before the treatment with 4 dyne/cm^2 FSS. 1 mM EGTA was used to chelate extracellular calcium before FSS. n=27, 9, 9 and 12 cells for DMSO, Thapsigargin (Tg), EGTA and si-*LMNA*, respectively. Representative immunofluorescence images **(e)** and quantification **(f)** of p-MLC in non-adherent

MDA-MB-231 cells after shear and Tg treatment. MBA-MB-231 cells were treated with Tg two days before FSS. n=34, 28, 30 and 31 cells for 0 dyne/cm^2 with DMSO, 0 dyne/cm^2 with Tg, 4 dyne/cm^2 with DMSO and 4 dyne/cm^2 with Tg, respectively. Scale bar, 5 μm. Representative immunofluorescence images **(g)** and quantification **(h)** of p-MLC in non-adherent MDA-MB-231 cells after shear and EGTA treatment. EGTA was added into the medium for an hour before FSS. n=31, 30, 28 and 31 cells for 0 dyne/cm^2 with DMSO, 0 dyne/cm^2 with EGTA, 4 dyne/cm^2 with DMSO and 4 dyne/cm^2 with EGTA, respectively. Scale bar, 5 μm. Data were summarized from 3 biological repeats and presented as mean ± SEM. The one-way ANOVA followed by Tukey's post-hoc test was adopted for comparison in **(d)**. Kruskal–Wallis one-way ANOVA followed by Mann–Whitney U-test with Bonferroni correction was adopted for comparison in **(b-c)**, **(f)** and **(h)**. ns, no significant difference; **, p <0.01 and ***, p < 0.001.

Figure S25 PLL-attached MCF-7 cells exhibit distinct responses of MLCK and ROCK to FSS. Representative immunofluorescence images **(a)** and quantification of MLCK in whole cell **(b)**, cortex **(c)**, cytoplasm **(d)**, and of MLCK cortex/cytoplasm ratio **(e)** in non-adherent tumor cells after various levels of FSS treatment. Non-adherent tumor cells were attached to PLL-coated microfluidic chips and treated under varying levels of FSS for one hour. n=50, 47 and 47 cells for 0, 1 and 4 dyne/cm^2 FSS, respectively. Scale bar, 5 μm. Representative immunofluorescence images **(f)** and quantification of phosphorylated ROCK in whole cell **(g)**, cortex **(h)**, cytoplasm **(i)**, and of phosphorylated ROCK cortex/cytoplasm ratio **(j)** in non-adherent tumor cells after various levels of FSS treatment. Non-adherent tumor cells were attached to PLL-coated microfluidic chips and treated under varying levels of FSS for one hour. n=32, 28 and 28 cells for 0, 1 and 4 dyne/cm^2 FSS, respectively. Scale bar, 5 μm. Data were summarized from 3 biological repeats and presented as mean ± SEM. The one-way ANOVA followed by Tukey's post-hoc test was adopted for comparison in **(g-i)**. Kruskal–Wallis one-way ANOVA followed by Mann–Whitney U-test with Bonferroni correction was adopted for comparison in **(b-e)** and **(j)**. ns, no significant difference; *, p <0.05, **, p <0.01 and ***, p < 0.001.

Figure S26 Non-adherent MCF-7 cells in tubing exhibit distinct responses of MLCK and ROCK to FSS. Representative immunofluorescence images **(a)** and quantification of MLCK in whole cell **(b)**, cortex **(c)**, cytoplasm **(d)**, and of MLCK cortex/cytoplasm ratio **(e)** in non-adherent tumor cells after various levels of FSS treatment. Non-adherent tumor cells were circulated under 0, 2 and 10 dyne/cm^2 FSS for an hour in the *in vitro* microfluidic system. n=29, 29 and 33 cells for 0, 2 and 10 dyne/cm^2 FSS, respectively. Scale bar, 5 μm. Representative immunofluorescence images **(f)** and quantification of phosphorylated ROCK in whole cell **(g)**, cortex **(h)**, cytoplasm **(i)**, and of phosphorylated ROCK cortex/cytoplasm ratio **(j)** in non-adherent tumor cells after various levels of FSS treatment. Non-adherent tumor cells were circulated under 0, 2 and 10 dyne/cm^2 FSS for an hour in the *in vitro* microfluidic system. n=28, 32 and 31 cells for 0, 2 and 10 dyne/cm^2 FSS, respectively. Scale bar, 5 μm. Data were summarized from 3 biological repeats and presented as mean ± SEM. The one-way ANOVA followed by Tukey's post-hoc test was adopted for comparison in **(e)** and **(j)**. Kruskal–Wallis one-way ANOVA followed by Mann–Whitney U-test with Bonferroni correction was adopted for comparison in **(b-d)** and **(g-i)**. ns, no significant difference; *, p <0.05, **, p <0.01 and ***, p < 0.001.

Figure S27 Nuclear mechanosensing mediates the effects of FSS on MLCK and ROCK in non-adherent tumor cells. Representative immunofluorescence images **(a)** and quantification of MLCK in whole cell **(b)**, cortex **(c),** and cytoplasm **(d)** in non-adherent tumor cells after shear treatment and *LMNA* knockdown. *LMNA* was knocked down two days before FSS. n=23, 23, 22 and 25 cells for 0 dyne/cm^2 with Control, 0 dyne/cm^2 with si-*LMNA*, 4 dyne/cm^2 with Control and 4 dyne/cm^2 with si-*LMNA*, respectively. Scale bar, 5 μm. Representative immunofluorescence images **(e)** and quantification of phosphorylated ROCK in whole cell **(f)**, cortex **(g)**, and cytoplasm **(h)** in non-adherent tumor cells after shear treatment and *LMNA* knockdown. *LMNA* was knocked down two days before FSS. n=31, 33, 31 and 30 cells for 0 dyne/cm^2 with Control, 0 dyne/cm^2 with si-*LMNA*, 4 dyne/cm^2 with Control and 4 dyne/cm^2 with si-*LMNA*, respectively. Scale bar, 5 μm. Representative immunofluorescence images **(i)**

and quantification of MLCK in whole cell **(j)**, cortex **(k)**, and cytoplasm **(l)** in non-adherent tumor cells after shear treatment and Tg treatment. MCF-7 cells were treated with Tg two days before FSS. n=21, 25, 25 and 22 cells for 0 dyne/cm^2 with DMSO, 0 dyne/cm^2 with Tg, 4 dyne/cm^2 with DMSO and 4 dyne/cm^2 with Tg, respectively. Scale bar, 5 μm. Representative immunofluorescence images **(m)** and quantification of phosphorylated ROCK in whole cell **(n)**, cortex **(o)**, and cytoplasm **(p)** in non-adherent tumor cells after shear and Tg treatment. MCF-7 cells were treated with Tg two days before FSS. n=23, 27, 33 and 30 cells for 0 dyne/cm^2 with DMSO, 0 dyne/cm^2 with Tg, 4 dyne/cm^2 with DMSO and 4 dyne/cm^2 with Tg, respectively. Scale bar, 5 μm. Data were summarized from 3 biological repeats and presented as mean ± SEM. The one-way ANOVA followed by Tukey's post-hoc test was adopted for comparison in **(k)**. Kruskal–Wallis one-way ANOVA followed by Mann–Whitney U-test with Bonferroni correction was adopted for comparison in **(b-d)**, **(f-h)**, **(j)**, **(l)** and **(n-p)**. ns, no significant difference; *, p <0.05, **, p <0.01 and ***, p < 0.001.

Figure S28 Knockdown of *MYH9* or *MYH10* increases DNA damage and cell apoptosis of non-adherent tumor cells under FSS. Representative immunofluorescence images **(a)** and quantification **(b)** of the percentage of γ-H2AX+ cells after shear treatment and *MYH9* knockdown. *MYH9* was knocked down two days before FSS. n=3 biological repeats for each condition. Scale bar, 5 μm. Representative immunofluorescence images **(c)** and quantification **(d)** of the percentage of γ-H2AX+ cells after shear treatment and *MYH10* knockdown. *MYH10* was knocked down two days before FSS. n=3 biological repeats for each condition. Scale bar, 5 μm. **(e)** The percentage of apoptotic cells after shear and Y27632 treatments. Cell survival was analyzed by PI apoptosis assay. n=3 biological repeats for each condition. **(f)** The percentage of apoptotic cells after shear and genetic treatments. *MYH9* and *MYH10* were knocked down two days before FSS. Cell survival was analyzed by PI apoptosis assay. n=3 biological repeats. Data were presented as mean ± SEM. The one-way ANOVA followed by Tukey's post-hoc test was adopted for comparison in **(b)** and **(d-f)**. ns, no significant difference; *, p <0.05, **, p <0.01 and ***, p < 0.001.

Figure S29 Verification of the stemness in fibrin gel-selected cancer stem cells. Representative immunofluorescence images **(a)** and quantification **(b)** of CD24 before and after fibrin gel selection. MCF-7 cells were selected by fibrin gels for five days. n=31 cells for each condition. Scale bar, 5 μm. Representative immunofluorescence images **(c)** and quantification **(d)** of CD44 before and after fibrin gel selection. MCF-7 cells were selected by fibrin gel for five days. n=31 cells for each condition. Scale bar, 5 μm. Representative immunofluorescence images **(e)** and quantification of Sox2 **(f)** and Oct4 **(g)** before and after fibrin gel selection. MCF-7 cells were selected by fibrin gel for five days. n=32 and 41 cells for non-CSC and CSC in **(f)**, respectively. n=39 and 34 cells for non-CSC and CSC in **(g)**, respectively. Scale bar, 5 μm. Representative immunofluorescence images **(h)** and quantification **(i)** of Nanog before and after fibrin gel selection. MCF-7 cells were selected by fibrin gel for five days. n=36 and 35 cells for non-CSC and CSC, respectively. Scale bar, 5 μm. Data were summarized from 3 biological repeats and presented as mean ± SEM. Unpaired student *t*-test was adopted for comparison in **(b)**. Mann–Whitney U-test was adopted for comparison in **(d)**, **(f-g)** and **(i)**. ***, $p < 0.001$.

Figure S30 The resistance of cancer stem cells to high FSS depends on cytoplasmic myosin-mediated mechanoadaptation. Representative immunofluorescence images **(a)** and quantification of p-MLC in whole cells **(b)** and cytoplasm **(c)** of non-CSC and CSC after the treatment under 0 or 2 dyne/cm^2 FSS for an hour. n=19, 19, 21 and 23 cells from 3 biological repeat for 0 dyne/cm^2 in non-CSC, 2 dyne/cm^2 in non-CSC, 0 dyne/cm^2 in CSC and 2 dyne/cm^2 in CSC, respectively. Scale bar, 5 μm. **(d)** Normalized FTE of non-CSC and CSC after Y27632 treatment. n=6, 7 and 7 cells from 3 biological repeats for non-CSC, CSC and CSC treated with Y27632, respectively. **(e)** The percentage of γ-H2AX+ cells after shear and Y27632 treatment in CSCs. CSC were treated with Y27632 under 4 dyne/cm^2 FSS. n=3 biological repeats. Data were presented as mean ± SEM. The one-way ANOVA followed by Tukey's post-hoc test was adopted for comparison in **(b-c)** and **(e)**. Kruskal–Wallis one-way ANOVA followed by Mann–Whitney U-test with Bonferroni correction was adopted for

comparison in **(d)**. ns, no significant difference; *, p <0.05, **, p <0.01 and ***, p < 0.001.

Figure S31 The resistance of jurkat cells to high FSS depends on cytoplasmic myosin-mediated mechanoadaptation. Representative immunofluorescence images **(a)** and quantification of p-MLC in whole cell **(b)**, cortex and cytoplasm **(c)** in non-adherent MCF-7 and jurkat cells. n=25 and 30 cells for MCF-7 and jurkat clls, respectively. Scale bar, 5 μm. Representative immunofluorescence images **(d)** and quantification **(e)** of cytoplasmic p-MLC after Y27632 treatment. Jurkat cells were treated with Y27632 for 10 minutes. n=33, 32 and 27 cells for MCF-7 with DMSO, jurkat with DMSO and jurkat with Y27632, respectively. Scale bar, 5 μm. **(f)** Normalized FTE of MCF-7 and jurkat cells after Y27632 treatment. Jurkat cells were treated with Y27632 for 10 minutes. n=14, 14 and 7 cells for MCF-7, jurkat with DMSO and jurkat with Y27632, respectively. Representative images **(g)** and quantification **(h)** of PI^+ cells/total after shear and Y27632 treatment for 6 hours in jurkat cells. Jurkat cells were treated with Y27632 during FSS treatment. n=7, 7, 14 and 14 cells for 0 dyne/cm^2 with DMSO, 0 dyne/cm^2 with Y27632, 4 dyne/cm^2 with DMSO and 4 dyne/cm^2 with Y27632, respectively. Scale bar, 100 μm. Data were summarized from 3 biological repeats and presented as mean ± SEM. Unpaired student *t*-test was adopted for comparison in **(b)**. Mann–Whitney U-test was adopted for comparison in **(c)**. Kruskal–Wallis one-way ANOVA followed by Mann–Whitney U-test with Bonferroni correction was adopted for comparison in **(e-f)**. The one-way ANOVA followed by Tukey's post-hoc test was adopted for comparison in **(h)**. ns, no significant difference; *, p <0.05, **, p <0.01 and ***, p < 0.001.

Figure S32 The resistance of BxPC3 cells to high FSS depends on cytoplasmic myosin-mediated mechanoadaptation. Representative immunofluorescence images **(a)** and quantification **(b)** of nuclear cleaved caspase 3 after the treatment under varying levels of FSS. Non-adherent BxPC3 cells were circulated under 0, 0.5, 1, 2, 5, 10 and 20 dyne/cm^2 FSS for an hour in the *in vitro* microfluidic system. The red and blue lines

represent the linear regression of data within the ranges of low and high FSS. n=38, 40, 47, 47, 51, 49 and 49 cells for 0, 0.5, 1, 2, 5, 10 and 20 dyne/cm^2, respectively. Scale bar, 5 μm. Representative immunofluorescence images **(c)** and quantification of p-MLC in whole cell **(d)**, cortex **(e)** and cytoplasm **(f),** and of p-MLC cortical/cytoplasmic ratio **(g)** after the treatment of 0, 1 and 4 dyne/cm^2 FSS for an hour in non-adherent BxPC3 cells. n=29, 32 and 36 cells for 0, 1 and 4 dyne/cm^2 FSS, respectively. Scale bar, 5 μm. **(h)** Normalized FTE of non-adherent BxPC3 cells after the pre-treatment of 0, 1 and 4 dyne/cm^2 FSS for an hour. n=12 cells for each condition. Data were summarized from 3 biological repeats and presented as mean ± SEM. The one-way ANOVA followed by Tukey's post-hoc test was adopted for comparison in **(d-e)**. Kruskal–Wallis one-way ANOVA followed by Mann–Whitney U-test with Bonferroni correction was adopted for comparison in **(f-h)**. ns, no significant difference; **, p <0.01 and ***, p < 0.001.

Figure S33 The resistance of A549 cells to high FSS depends on cytoplasmic myosin-mediated mechanoadaptation. Representative immunofluorescence images **(a)** and quantification **(b)** of nuclear cleaved caspase 3 after the treatment under varying levels of FSS. Non-adherent A549 cells were circulated under 0, 0.5, 1, 2, 5, 10 and 20 dyne/cm^2 FSS for an hour in the *in vitro* microfluidic system. The red and blue lines represent the linear regression of data within the ranges of low and high FSS. n=33, 30, 34, 38, 30, 32 and 42 cells for 0, 0.5, 1, 2, 5, 10 and 20 dyne/cm^2, respectively. Scale bar, 5 μm. Representative immunofluorescence images **(c)** and quantification of p-MLC in whole cell **(d)**, cortex **(e)** and cytoplasm **(f),** and of p-MLC cortical/cytoplasmic ratio **(g)** after the treatment of 0, 1 and 4 dyne/cm^2 FSS for an hour in non-adherent A549 cells. n=33, 35 and 33 cells for 0, 1 and 4 dyne/cm^2 FSS, respectively. Scale bar, 5 μm. **(h)** Normalized FTE of non-adherent A549 cells after the pre-treatment of 0, 1 and 4 dyne/cm^2 FSS for an hour. n=10 cells for each condition. Data were summarized from 3 biological repeats and presented as mean ± SEM. The one-way ANOVA followed by Tukey's post-hoc test was adopted for comparison in **(e)** and **(g)**. Kruskal–Wallis one-way ANOVA followed by Mann–Whitney U-test with Bonferroni correction was

adopted for comparison in **(d)**, **(f)** and **(h)**. ns, no significant difference; *, p <0.05, **, p <0.01 and ***, p < 0.001.

Figure S34 Cortical myosin II is dispensable for myosin-actin binding and FTE in patient-derived primary tumor cells under high FSS. Representative immunofluorescence images **(a)**, the quantification of p-MLC in whole cell **(b)**, cortex **(c)** and cytoplasm **(d)** after shear and ML-7 treatment. Primary tumor cells were attached to a PLL-coated microfluidic chip and treated under 0 and 4 dyne/cm^2 FSS for an hour with or without ML-7. n=47, 50 and 42 cells for 0 dyne/cm^2 with DMSO, 4 dyne/cm^2 with DMSO and 4 dyne/cm^2 with ML-7, respectively. Scale bar, 5 μm. **(e)** Co-localization of p-MLC with F-actin after shear and ML-7 treatment. Primary tumor cells were treated similarly as in **(a)**. The co-localization of p-MLC with F-actin in cytoplasm was analyzed by calculating the Pearson correlation coefficient. n=39, 42 and 47 cells for 0 dyne/cm^2 with DMSO, 4 dyne/cm^2 with DMSO and 4 dyne/cm^2 with ML-7, respectively. Representative PLA images of myosin IIA and β-actin **(f)** and the quantification **(g)** of PLA dots in cytoplasm after shear and ML-7 treatment. Primary tumor cells were treated similarly as in **(e)**. The interaction between myosin IIA and β-actin was detected using PLA assay. White arrows indicated typical PLA dots. n=27 cells for each condition. Scale bar, 5 μm. **(h)** Normalized FTE of primary tumor cells after the shear and ML-7 treatment. Primary tumor cells were treated similarly as in **(e)**. n=10 cells for each condition. Data were summarized from 3 biological repeats and presented as mean ± SEM. The one-way ANOVA followed by Tukey's post-hoc test was adopted for comparison in **(b)** and **(g)**. Kruskal–Wallis one-way ANOVA followed by Mann–Whitney U-test with Bonferroni correction was adopted for comparison in **(c-e)** and **(h)**. ns, no significant difference; *, p <0.05; **, p < 0.01 and ***, p < 0.001.

Figure S35 Inhibition of cytoplasmic myosin re-sensitives CTCs to shear-induced destruction *in vivo*. Representative image **(a)** and quantification **(b)** of bioluminescence signal after Triton X-100 treatment in MDA-MB-231 cells that stably expressed pNLuc (231-pNLuc). Representative images **(c)** and quantification **(d)** of

radiant efficient of the blood retrieved from mice. 231-pNLuc cells were treated with DMSO or Y27632 for an hour before the inoculation into mice through tail vein injection. After 12 hours, the blood of mice was collected for bioluminescence imaging after the reaction with coelenterazine. n=5 mice for each condition. Mann–Whitney U-test was adopted for comparison in **(d)**. *, *p* <0.05.

Figure S36 Transient inhibition of myosin II activity via doxycycline-inducible shROCK2. Representative immunofluorescence images **(a)** and quantification **(b)** of phosphorylated ROCK after doxycycline induction for 48 hours. MDA-MB-231 cells were stably transfected with doxycycline-inducible shROCK2 (Doxy-shROCK2). n=25, 30 and 31 cells for DMSO, 50 ng/mL doxycycline induction and 100 ng/mL doxycycline induction, respectively. Scale bar, 5 μm. Representative immunofluorescence images **(c)** and quantification of p-MLC in whole cell **(d)** and cytoplasm **(e)** after doxycycline induction for 48 hours. n=31, 30 and 35 cells for DMSO, 50 ng/mL doxycycline induction and 100 ng/mL doxycycline induction, respectively. Scale bar, 5 μm. **(f)** Normalized FTE of non-adherent MDA-MB-231 cells after doxycycline induction. n=7 cells for each condition.Data were summarized from 3 biological repeats and presented as mean ± SEM. Kruskal–Wallis one-way ANOVA followed by Mann–Whitney U-test with Bonferroni correction was adopted for comparison in **(b)** and **(d-f)**. ns, no significant difference; *, *p* <0.05, **, *p* <0.01 and ***, *p* < 0.001.

Figure S37 Phosphorylated ROCK and myosin activity are restored after the withdrawal of doxycycline in Doxy-shROCK2 cells. Representative immunofluorescence images **(a)** and quantification **(b)** of phosphorylated ROCK after doxycycline withdrawal. MDA-MB-231 cells were stably transfected with Doxy-shROCK2 and then treated with doxycycline for 48 hours (+Doxy 48h). After that, doxycycline was removed for 24 (-Doxy 24h) and 48 hours (-Doxy 48h), respectively. n=31, 30, 33 and 33 cells for DMSO, +Doxy 48h, -Doxy 24h and -Doxy 48h, respectively. Scale bar, 5 μm. Representative immunofluorescence images **(c)** and

quantification of p-MLC in whole cell **(d)** and cytoplasm **(e)** after doxycycline withdrawal. n=31, 32, 30 and 40 cells for DMSO, +Doxy 48h, -Doxy 24h and -Doxy 48h , respectively. Scale bar, 5 μm. **(f)** Normalized FTE after doxycycline withdrawal. Dox-shROCK cells were treated with Doxycycline for 48 hours followed by the withdrawal of Doxycycline for 48 hours. n=7 cells for each condition. Representative immunofluorescence images **(g)** and quantification **(h)** of p-MLC after doxycycline treatment in MDA-MB-231 wild type cells. n=35, 30 and 31 cells for DMSO, 100 ng/mL doxycycline treatment for 24 hours and 100 ng/mL doxycycline treatment for 48 hours, respectively. Scale bar, 5 μm. **(i)** Normalized FTE after 100 ng/mL doxycycline treatment in MDA-MB-231 wild type cells. n=15 and 14 cells for DMSO and +Doxy 48h, respectively. Data were summarized from 3 biological repeats and presented as mean ± SEM. The one-way ANOVA followed by Tukey's post-hoc test was adopted for comparison in **(b)** and **(h)**. Kruskal–Wallis one-way ANOVA followed by Mann–Whitney U-test with Bonferroni correction was adopted for comparison in **(d-e)** and **(f)**. Mann–Whitney U-test was adopted for comparison in **(i)**. ns, no significant difference; *, p <0.05, **, p <0.01 and ***, p < 0.001.

Figure S38 Cell migration and invasion abilities are restored after the withdrawal of doxycycline in Doxy-shROCK2 cells. (a) Representative images of transwell migration (top) and invasion (bottom) assay after Y27632 treatment. Scale bar, 50 μm. **(b-c)** Quantification of migrated **(b)** and invaded cells **(c)** per view after Y27632 treatment. 20 μM Y27632 was used to treat cells for 24 hours before transwell assay. n=9 views from 3 biological repeats in **(b)** and n=8 views from 3 biological repeats in **(c)**. **(d)** Representative images of transwell migration (top) and invasion (bottom) assay after doxycycline withdrawal. Scale bar, 50 μm. **(e-f)** Quantification of migrated **(e)** and invaded cells **(f)** per view after doxycycline withdrawal. n=8 views from 3 biological repeats. Data were presented as mean ± SEM. Unpaired student *t*-test was adopted for comparison in **(b-c)**. The one-way ANOVA followed by Tukey's post-hoc test was adopted for comparison in **(e-f)**.

Figure S39 Tropomyosin activity of non-adherent tumor cells is altered under high FSS. (a) Schematic of signaling pathways in mechanoadapatation of non-adherent tumor cells to high FSS. **(b-c)** Representative immunofluorescence images **(b)** and quantification **(c)** of the p-tropomyosin after shear treatment. n=33, 32 and 35 cells for 0 dyne/cm^2, 1 dyne/cm^2 and 4 dyne/cm^2, respectively. Scale bar, 5 μm. **(d-e)** Representative immunofluorescence images **(d)** and quantification **(e)** of p-tropomyosin s after shear and Y27632 treatment. Y27632 was used to treat cells during FSS. n=32, 30 and 32 cells for 0 dyne/cm^2 with DMSO, 4 dyne/cm^2 with DMSO and 4 dyne/cm^2 with Y27632, respectively. Scale bar, 5 μm. Data were summarized from 3 biological repeats and presented as mean ± SEM. The one-way ANOVA followed by Tukey's post-hoc test was adopted for comparison in **(c)**. Kruskal–Wallis one-way ANOVA followed by Mann–Whitney U-test with Bonferroni correction was adopted for comparison in **(e)**. ns, no significant difference; *, p <0.05 and ***, p < 0.001.

Table 1. Sequences of siRNA and primers used in this study

Gene Name	Forward sequence	Reverse sequence
si*MLCK*	CAAUUCAGAUGUCAGGAAATT	UUUCCUGACAUCUGAAUUGTT
si*LMNA*	GCGCAAACUGGAGUCCACUGA	AGUGGACUCCAGUUUGCGCUU
si*MYH9*#1	GGAGCCAACAUUGAGACUUTT	AAGUCUCAAUGUUGGCUCCTT
si*MYH9*#2	CCAAGCAGCUGAAGGACAATT	UUGUCCUUCAGCUUCUUGGTT
si*MYH9*#3	GCAAACCUCGAGAAGGCAATT	UUGCCUUCUCGAGGUUUGCTT
si*MYH10*#1	GAGAAGAAGCUGAAAGAAATT	UUUCUUUCAGCUUCUUCUCTT
si*MYH10*#2	CCAAAGAUGAUGUGGGAAATT	UUUCCCACAUCAUCUUUGGTT
si*MYH10*#3	UGGAAGAAGCAGAGAAGAATT	UUCUUCUCUGCUUCUUCCATT
GADPH	GUAUGACAACAGCCUCAAGTT	CUUGAGGCUGUUGUCAUACTT
MYH9	ATCCTGGAGGACCAGAACTGCA	GGCGAGGCTCTTAGATTTCTCC
MYH10	GCTGATGGCAACTCTCCGAAAC	CTTCCAGGACACCATTACAGCG

Table 2. Parameters used in DPD simulations

Symbol	Description	DPD unit	Physical unit	Reference
**Principal properties**				
$[L]$	Length scale	1	$0.5\ \mu m$	Estimated
$[F]$	Force scale	1	$0.043\ pN$	Estimated
$[t]$	Time scale	1	$4.42\ ms$	Estimated
**Cortex properties**				
D_c	Diameter	32	$16\ \mu m$	This work
K_c	Spring stiffness of network	100	$8.66\ \mu N/m$	Estimated[1-3]
$r_{0,c}$	Equilibrium bond length	0.5	$0.25\ \mu m$	4
**Nuclear lamina properties**				
D_n	Diameter	20	$10\ \mu m$	This work
K_n	Spring stiffness of network	20	$1.73\ \mu N/m$	$(= K_c/5)$
$r_{0,n}$	Equilibrium bond length	0.5	$0.25\ \mu m$	$(= r_{0,c})$
**Actin filament properties**				
K_p	Spring stiffness	200	$17.32\ \mu N/m$	$(= 2K_c)$
$r_{0,p}$	Equilibrium bond length	0.5	$0.25\ \mu m$	$(= r_{0,c})$
K_θ	Angle stiffness	10	$2.17 \times 10^{-19}\ J$	Assumed
θ_0	Equilibrium angle	$180°$	$180°$	Assumed
**Myosin II properties**				
K_m	Spring stiffness	100	$8.66\ \mu N/m$	$(= K_c)$
$r_{0,m}$	Equilibrium bond length	0.5	$0.25\ \mu m$	$(= r_{0,c})$
k_{on}^0	Force-free binding rate	0.1	$22.64\ s^{-1}$	5,6
k_{off}^0	Force-free unbinding rate	0.0005	$0.11\ s^{-1}$	5,6
F_{off}	Characteristic unbinding force	5	$0.22\ pN$	Assumed
k_{fw}^0	Force-free forward rate	0.01	$2.26\ s^{-1}$	5,6
F_{fw}	Characteristic forward force	5	$0.22\ pN$	Assumed
k_{bw}^0	Force-free backward rate	0.001	$0.23\ s^{-1}$	5,6
F_{bw}	Characteristic backward force	10	$0.43\ pN$	Assumed
**Fluid properties**				
η	Dynamic viscosity of fluid	1.31	$1.002\ mPa \cdot s$	7
τ	Fluid shear stress	$0{\sim}5.86$	$0{\sim}1.01\ Pa$	This work

Table 3. DPD potential parameter a_{ij} for different bead types

a_{ij}	nucleus	cortex	nucleoplasm	cytoplasm	actomyosin	fluid	plate
nucleus	25	100	30	30	50	30	100
cortex		25	30	30	50	30	100
nucleoplasm			25	100	100	100	50
cytoplasm				25	27	100	50
actomyosin		Symmetry			25	100	100
fluid						25	50
plate							25

Reference

1. Seung, H. S. & Nelson, D. R. Defects in flexible membranes with crystalline order. *Phys. Rev. A* **38**, 1005–1018 (1988).

2. Omori, T. *et al.* Comparison between spring network models and continuum constitutive laws: Application to the large deformation of a capsule in shear flow. *Phys. Rev. E* **83**, 041918 (2011).

3. Massey, A. *et al.* Mechanical properties of human tumour tissues and their implications for cancer development. *Nat. Rev. Phys.* **6**, 269–282 (2024).

4. Svitkina, T. M. Actin Cell Cortex: Structure and Molecular Organization. *Trends Cell Biol.* **30**, 556–565 (2020).

5. Erdmann, T., Albert, P. J. & Schwarz, U. S. Stochastic dynamics of small ensembles of non-processive molecular motors: The parallel cluster model. *J. Chem. Phys.* **139**, 175104 (2013).

6. Woody, M. S., Winkelmann, D. A., Capitanio, M., Ostap, E. M. & Goldman, Y. E. Single molecule mechanics resolves the earliest events in force generation by cardiac myosin. *eLife* **8**, e49266 (2019).

7. Kestin, J., Sokolov, M. & Wakeham, W. A. Viscosity of liquid water in the range −8 °C to 150 °C. *J. Phys. Chem. Ref. Data* **7**, 941–948 (1978).
